# The global prevalence of headache: an update, with analysis of the influences of methodological factors on prevalence estimates

**DOI:** 10.1186/s10194-022-01402-2

**Published:** 2022-04-12

**Authors:** Lars Jacob Stovner, Knut Hagen, Mattias Linde, Timothy J. Steiner

**Affiliations:** 1 Department of Neuromedicine and Movement Science, NTNU Norwegian University of Science and Technology, Trondheim, Norway; 2 Department of Neurology and Clinical Neurophysiology, Norwegian Advisory Unit on Headache, St Olavs University Hospital, Trondheim, Norway; 3 NTNU Norwegian University of Science and Technology, Trondheim, Norway; 4 Tjörns Headache Clinic, Rönnäng, Sweden; 5 Division of Brain Sciences, Imperial College London, London, UK

**Keywords:** Headache, Methodology, Migraine, Prevalence, Narrative review, Tension-type headache, Global campaign against headache

## Abstract

**Background:**

According to the Global Burden of Disease (GBD) study, headache disorders are among the most prevalent and disabling conditions worldwide. GBD builds on epidemiological studies (published and unpublished) which are notable for wide variations in both their methodologies and their prevalence estimates.

Our first aim was to update the documentation of headache epidemiological studies, summarizing global prevalence estimates for all headache, migraine, tension-type headache (TTH) and headache on ≥15 days/month (H15+), comparing these with GBD estimates and exploring time trends and geographical variations. Our second aim was to analyse how methodological factors influenced prevalence estimates.

**Methods:**

In a narrative review, all prevalence studies published until 2020, excluding those of clinic populations, were identified through a literature search. Prevalence data were extracted, along with those related to methodology, world region and publication year. Bivariate analyses (correlations or comparisons of means) and multiple linear regression (MLR) analyses were performed.

**Results:**

From 357 publications, the vast majority from high-income countries, the estimated global prevalence of active headache disorder was 52.0% (95%CI 48.9–55.4), of migraine 14.0% (12.9–15.2), of TTH 26.0% (22.7–29.5) and of H15+ 4.6% (3.9–5.5). These estimates were comparable with those of migraine and TTH in GBD2019, the most recent iteration, but higher for headache overall. Each day, 15.8% of the world’s population had headache. MLR analyses explained less than 30% of the variation. Methodological factors contributing to variation, were publication year, sample size, inclusion of probable diagnoses, sub-population sampling (e.g., of health-care personnel), sampling method (random or not), screening question (neutral, or qualified in severity or presumed cause) and scope of enquiry (headache disorders only or multiple other conditions). With these taken into account, migraine prevalence estimates increased over the years, while estimates for all headache types varied between world regions.

**Conclusion:**

The review confirms GBD in finding that headache disorders remain highly prevalent worldwide, and it identifies methodological factors explaining some of the large variation between study findings. These variations render uncertain both the increase in migraine prevalence estimates over time, and the geographical differences. More and better studies are needed in low- and middle-income countries.

**Supplementary Information:**

The online version contains supplementary material available at 10.1186/s10194-022-01402-2.

## Introduction

Documenting the burden of headache disorders has become an important task, brought to attention by the Global Campaign against Headache [1]. Through the Global Burden of Disease (GBD) study, headache disorders are revealed as one of the major public-health concerns globally and in all countries and world regions [2]. In the 2019 iteration (GBD2019), migraine alone was second among the causes of disability, and first among women under 50 years of age [3]. GBD estimates are now updated annually to monitor changes in disease burden around the globe, and thereby forecast future needs in health services.

For the various disorders it considers, GBD uses multiple data sources (epidemiological studies, health registers, official statistics, hospital data etc) to make best-informed estimates of prevalence and burden. For headache disorders, however, epidemiological studies are the only source, but these are now available from many countries, including several yet unpublished from the Global Campaign. While GBD handles data for all diseases in a relatively uniform way, the quality of its estimates for each disease depends on a good understanding of the particular methodological issues that may influence them. Headache epidemiology is a relatively young and immature discipline [4]. Studies follow general epidemiological principles, but the field has its peculiar methodological problems [4]. It is believed that the large variations in reported prevalences from country to country, and sometimes within countries, are to a large extent caused by methodological differences between studies, but this has not been explored empirically.

Guidelines on performing and reporting headache epidemiological studies were published in 2014 [4]. They included criteria for judging the quality of studies from their reported methodology, and some adjustments to prevalence estimates were based upon these in the most detailed analysis of headache data, from GBD 2016 [2]. Nevertheless, these criteria were mostly theoretical, derived from general principles and expert opinion (see [2] Appendix). A better understanding of which methodological factors influence findings, and how, may be gained by reviewing metadata from all studies regarding how they collected and processed prevalence data, and analyzing findings across these studies accordingly.

In 2007 we reviewed all published studies of the prevalence and burden of headache [5]. This review, highly cited, constituted the basis for collaboration between GBD and the Global Campaign against Headache [1] from GBD2010 onwards. Since the review, many new studies have provided more insight into the importance and influences of methodological issues, and this additional insight informed the methodological guidelines [4]. Here we update that review, and the documentation of headache epidemiological studies, summarizing global prevalence estimates for headache, migraine, tension-type headache (TTH) and headache on ≥15 days/month (H15+), comparing these with GBD estimates and exploring time trends and geographical variations. We also use the data and material from all the studies to analyse empirically how methodological factors might influence prevalence estimates.

## Methods

### Literature search and data extraction

A new literature search through PubMed, using the search terms “headache epidemiology”, “migraine epidemiology”, “headache prevalence” or “migraine prevalence”, identified studies published till the end of 2020. The search had been performed several times by LJS, KH and ML since our 2007 publication [5], and was concluded by LJS during the first month of 2021.

This proved to be a time-consuming task. The search terms “headache” and “prevalence” returned more than 18,000 articles in PubMed, and “migraine” and “epidemiology” more than 6000, the great majority irrelevant for our purpose, but we found no way to restrict the search: additional terms such as “population-based” excluded many relevant papers. To include all parts of the world and all settings that were possible, we accepted data from all non-clinical samples as well as those that were strictly population-based, which entailed browsing all the pages in PubMed returned by these search terms. While most irrelevant papers could be dismissed from the title alone and some from the abstract, with only a few requiring perusal of their full texts. This introduced a subjective element in the search that was not in conformity with accepted methodology for systematic review. But by not restricting the literature search to strictly population-based samples we created a database similar to (although larger than) the one used in GBD studies of headache. This made our investigation of methodological factors more relevant to future evaluations of GBD estimates.

Many additional studies were detected from the reference lists in relevant reviews (e.g. [6–8]). All articles were screened for population of interest, and excluded if reporting only samples derived from clinical sources (hospitals, medical practices, etc) or those described as patients or identified through patient groups.

We extracted prevalence data for all headache, migraine, TTH and H15+, overall and for each gender. We also extracted data on the various aspects of methodology and design described below. When studies failed to provide value for a variable of interest, we stipulated a value, assigned a separate value, or registered it as ‘unknown’ (specific examples are given below: Age of population, Timeframe of headache, Application of ICHD criteria).

### Age of population sample

Far from all studies reported either mean or median age of the population of interest or of the sample, but almost all indicated the age range (youngest to oldest). Therefore, we could not extract data for each age group but, instead, used the mid-range value as a uniform, albeit imprecise, indicator of population age. In the relatively few studies not even specifying this, for example only stating “adults”, we stipulated the range to be 20–85 years.

### Timeframe of headache

The International Classification of Headache Disorders (ICHD-3) specifies that “For most purposes, patients receive a diagnosis according to the headache phenotype *currently present or that has presented within the last year*” [9] (our emphasis). Nevertheless, to capture all studies that might be relevant for our purposes, we included those reporting 1-year, 6-month or 3-month timeframes, “current headache” or no specific timeframe, subsuming their findings into prevalence of “active headache disorder”. We separately considered lifetime prevalence and very short timeframes (headache now, or headache yesterday).

### Application of ICHD criteria with regard to definite and probable diagnoses

In studies using ICHD criteria or modifications of them (almost all those published after 1988), we expected prevalence estimates of migraine and TTH to be highly dependent on how these criteria were applied when diagnoses were uncertain because one of the criteria was not fulfilled. ICHD-I used the terms “migrainous disorder not fulfilling above criteria” (coded 1.7) and “headache of tension-type not fulfilling above criteria” (coded 2.3) [10], whereas later editions referred to “probable migraine” (coded 1.5) and “probable TTH” (coded 2.4) [9, 11]. We extracted prevalence data for each of these diagnostic categories when they were provided, and, when summarizing data, summed the estimates for definite and probable migraine (dMig and pMig) and for definite and probable TTH (dTTH and pTTH). Otherwise, when these were not separately provided, we used prevalences as reported.

### Screening question

As far as we could, we distinguished between studies with a neutral question (“have you had a headache?”) from those using questions qualified in some manner in terms of severity, frequency or presumed but unverified cause (“do you suffer from headache?”, “have you had severe/recurrent headaches?”, “have you had a headache not caused by hangover, common cold, flu or head trauma?”, etc) [12].

### Other study properties and quality measures

In addition to geographical origin and publication year, we extracted data related to the quality criteria [4]: those describing the population of interest (the general population or a specified sub-population), sampling method (randomness and representativeness), size of sample, participating proportion, methods of data collection (access to and engagement with participants) and validation of diagnostic questions. These, and whether ICHD criteria or reasonable modifications of them, and whether a suitable timeframe had been indicated (see above for each), were each scored 1–4, with a penalty, punitively set at − 4 [4], applied whenever a study provided no information on any criterion except timeframe (no information on this was almost invariably understood to mean “current headache”). A summed score for all eight criteria, theoretically ranging from − 28 to 32, was calculated for each study.

For MLR analyses we dichotomized the quality measures [4] that were not interval or ordinal variables:

- population of interest (unselected [general] population of a country, community or tribe, or pupils of obligatory schools, versus selected subpopulations [e.g., university students, factory/workplace employees, minorities, etc], or unstipulated [additionally, we registered whether selected subpopulations were health-care personnel such as medical students, hospital employees, neurologists, etc]);
- sample representativeness of the population of interest (random sampling versus non-random sampling or failed attempt to secure randomness);
- access to and engagement with participants (face-to-face or telephone interview versus unsupervised questionnaire completion or unstipulated);
- validation of diagnostic questions (effort at validating versus none or unstipulated);
- application of ICHD criteria and distinction between definite and probable diagnoses versus not or unstipulated.

We rated participating proportion in 10 categories (0: unknown; 1–8 for the deciles 0–9 to 70–79, and 9 for ≥80%). Studies in obligatory schools performed in class during school hours were accorded the value 9 even when the exact proportion was not given, or uncertain because of unrecorded absences, since such studies strongly encourage participation. Studies using convenience sampling (encountering people on the streets, or mailing or emailing questionnaires), which were generally unable to specify the denominator, and studies not stating the number addressed to achieve the desired number of participants, were rated − 4.

Additionally, although not a quality criterion, we distinguished between studies according to scope (restricted, with headache and/or one or more specified headache types the only diagnoses of interest, versus broader scope, headache being only one among many [often a range of neurological disorders or pain conditions]).

### Data handling and statistics

Many studies reported imbalance in the proportions of male and female participants, and not all adjusted the overall prevalence estimates accordingly. Since our interest was in estimates for the general population, with a gender distribution of 50/50, we calculated overall prevalence in all sampled populations as the mean of the estimates for males and females, even when, in sub-populations, distributions might be different. For this reason, we present some estimates that diverge somewhat from those published.

To summarize prevalence estimates from multiple studies (Tables 1, 2, 3, 4 and 5), we took the mean with 95% confidence intervals (CIs), also presenting median values for comparisons of the main prevalence estimates. CIs were calculated by bootstrapping (drawing 1000 random samples), a robust method when the underlying distribution is not normal or unknown. SPSS did not calculate CIs when there were ≤ 5 observations. In bivariate analyses (Table 6), correlations were measured with Pearson’s r, and means compared with Student’s t-test for independent samples or one-way ANOVA for several groups. Since testing was for exploratory purposes, we did not correct for multiple tests.

**Table 1:** Prevalence (%) of active headache disorder by type, age (mid-range values of studies) and gender

Gender/age (years)	Headache			Migraine			TTH			H15+		
	N	Mean (median)	95% CI	N	Mean (median)	95% CI	N	Mean (median)	95% CI	N	Mean (median)	95% CI
Both genders												
0–9	10	42.7	30.6–55.4	12	5.0	3.6–6.5	4	5.9	1.3–12.8	0	–	–
10–19	60	57.9	52.0–63.9	61	13.4	11.4–15.6	40	25.1	18.8–31.6	14	3.3	2.3–4.0
20–64	107	50.2	46.0–54.8	165	15.2	13.8–16.6	65	27.6	24.0–31.3	43	5.1	4.2–6.2
≥ 65	6	40.2	30.0–50.6	6	7.9	4.9–10.3	1	32.2	–	2	4.2	
All ages	183	52.0 (53.5)	48.9–55.4	244	14.0 (11.6)	12.9–15.2	110	26.0 (21.4)	22.7–29.5	59	4.6 (3.6)	3.9–5.5
Males												
0–9	2	40.4	–	11	4.7	3.3–6.1	4	5.4	–	0	–	–
10–19	39	51.4	43.8–58.6	36	10.2	8.4–12.2	16	25.5	16.2–35.5	6	1.4	0.7–2.2
20–64	71	41.8	36.5–47.6	128	8.7	7.6–9.8	43	24.2	18. 7–30.0	33	3.2	2.4–4.3
≥ 65	5	27.8	–	5	4.5	–	1	28.1	–	2	2.2	–
All ages	117	44.4 (46.2)	40.3–48.3	180	8.6 (7.4)	7.8–9.5	64	23.4 (17.9)	19.4–28.0	41	2.9 (1.9)	2.2–3.8
Females												
0–9	2	41.5	–	11	5.6	3.9–7.3	4	6.4	–	0	–	–
10–19	38	62.3	55.0–69.0	37	15.3	12.7–18.1	17	27.1	17.7–37.2	6	3.1	1.4–4.7
20–64	74	56.5	51.4–61.4	131	18.6	17.0–20.5	44	28.8	23.2–35.4	33	6.6	5.2–8.0
≥ 65	5	50.4	–	5	10.6	–	1	36.3	–	2	5.8	–
All ages	119	57.8 (60.0)	53.9–61.7	184	17.0 (15.5)	15.6–18.5	66	27.1 (21.0)	22.7–32.3	41	6.0 (5.2)	4.9–7.4

*N* number of studies, *TTH* tension-type headache, *H15+* headache on ≥15 days/month, *CI* confidence interval (not given when *N* ≤ 5), *SD* standard deviation

**Table 2:** Prevalence (%) of active headache disorder by type and geographical area (estimated in this study and, for migraine and TTH, in GBD2019)

		Headache			Migraine			TTH			H15+	
GBD superregion	%^1^	N^2^	Mean (95%CI)	GBD2019	N^1^	Mean (95% CI)	GBD2019	N^1^	Mean (95% CI)	GBD2019	N^1^	Mean (95% CI)
Global	100	183	52.0 (48.9–55.4)	35 (32.3–37.7)	244	14.0 (12.9–15.2)	15.2 (13.2–17.4)	110	26.0 (22.7–29.5)	26.8 (23.5–30.1)	59	4.6 (3.8–5.4)
CEECA	5.3	14	53.6 (41.8–65.2)	41.8 (38.6–44.9)	18	16.6 (12.6–21.3)	15.6 (13.6–17.9)	15	31.1 (22.8–39.2)	35.2 (31.3–39.0)	8	5.2 (3.3–7.2)
HI	14.7	88	51.9 (47.5–56.6)	43.4 (40.2–46.5)	117	13.7 (12.3–15.5)	17.2 (15.1–19.9)	41	30.4 (24.4–36.5)	36.4 (32.4–40.4)	24	3.4 (2.9–4.1)
LAC	7.4	24	55.0 (44.9–65.4)	36.7 (33.8–39.7)	33	17.0 (12.9–22.0)	16.3 (14.1–19.1)	11	26.8 (16.1–39.8)	27.9 (24.4–31.4)	6	6.4 (5.0–7.5)
SEEAO	27.6	10	38.7 (20.4–56.5)	37.3 (34.3–40.4)	16	9.0 (6.0–13.1)	17.5 (15.1–20.2)	8	11.1 (3.5–19.5)	27.7 (24.2–31.2)	5	2.8
NAME	7.7	20	60.5 (50.9–68.5)	35.4 (32.3–38.6)	30	11.8 (9.4–14.4)	16.7 (14.4–19.4)	19	20.5 (14.4–27.8)	25.7 (22.1–29.4)	7	4.9 (3.0–6.9)
SA	23.4	7	53.2 (29.0–74.1)	35.6 (32.7–38.6)	8	22.2 (16.1–27.8)	15.5 (13.4–17.9)	5	33.1 (20.3–42.9)	27.4 (24.1–31.0)	4	6.9
SSA	14.0	19	43.8 (32.4–55.6)	27.4 (24.6–30.2)	21	12.4 (9.1–16.8)	11.3 (9.5–13.3)	10	16.6 (12.4–21.0)	21.0 (17.9–24.4)	4	6.7

1% of the world’s population living in the superregion

2 The global total of studies is higher than the sum of studies per superregion because some studies give data from more than one superregion, i.e. it cannot be assigned to a single superregion

*H15+* headache on ≥15 days/month, %: percent of world population, N: number of studies, TTH: tension-type headache, CI: confidence interval (not given when *N* ≤ 5)

*CEECA* Central Europe, Eastern Europe and Central Asia, *HI* High-income countries, *LAC* Latin America and Caribbean, *SEEAO* Southeast Asia, East Asia and Oceania, *NAME* North Africa and Middle East, *SA* South Asia, *SSA* Sub-Saharan Africa

**Table 3:** Prevalence (%) of active headache disorder by type, gender and timeframe

Timeframe	Headache			Migraine			TTH			H15+		
	N	Mean	95% CI	N	Mean	95% CI	N	Mean	95% CI/	N	Mean	95% CI
Lifetime												
Both genders	42	66.6	58.8–73.9	49	17.5	14.6–21.0	18	40.6	30.0–51.4	9	4.5	1.8–7.7
Males	28	65.0	53.8–74.4	39	11.6	9.1–14.8	9	35.7	19.6–52.6	6	1.9	0.9–3.1
Females	28	73.3	63.3–83.2	41	21.0	17.3–25.4	9	43.8	26.5–61.8	6	4.5	2.2–7.5
1-year												
Both genders	110	53.5	49.1–57.7	150	15.3	13.7–16.9	77	26.4	22.9–30.3	50	4.8	4.0–5.7
Males	73	46.4	41.1–51.7	112	9.1	7.9–10.3	47	24.2	19.2–29.6	39	2.9	2.2–3.8
Females	72	60.5	55.1–65.8	113	18.1	16.3–29.9	48	28.5	22.7–34.3	39	6.2	4.9–7.6
6-month, both genders	15	60.9	50.6–71.3	12	11.1	6.7–16.9	9	32.2	15.1–50.8	2	6.0	4.8–7.2
3-month, both genders	12	52.2	36.5–66.6	10	13.4	9.1–17.9	5	20.2	2.1–38.6	3	2.7	2.2–3.3
“Current”, both genders	3	37.2	–	10	12.9	7.7–18.3	0	–	–	0	–	–
Unknown, both genders	43	45.9	38.5–53.1	62	11.9	10.0–14.0	19	22.9	17.2–29.2	4	3.4	1.5–5.4
1-day												
Both genders	12	15.8	10.0–22.0	5	7.0	–	4	8.7	–	3	2.5	–
Males	8	8.0	4.0–12.8	2	2.4	–	1	5.1	–	1	1.8	–
Females	8	14.9	8.9–21.7	2	5.5	–	1	10.1	–	1	4.4	–
All timeframes, both genders	237	52.7	49.7–55.8	298	14.5	13.4–15.5	132	27.4	24.2–30.9	71	4.5	3.9–5.2

*N* number of studies, *TTH* tension-type headache, *H15+* headache on ≥15 days/month, *CI* confidence interval (not given when N ≤ 5)

**Table 4:** Prevalence (%) of active headache disorder by type, gender and diagnostic certainty (definite or probable)

	Studies specifying both									Studies not making or not specifying the distinction			Definite+probable (where given) or unspecified		
	Definite			Probable			All (definite + probable)								
	N	Mean	95% CI	N	Mean	95% CI	N	Mean	95% CI	N	Mean	95% CI	N	Mean	95% CI
Migraine															
Both genders	59	10.5	9.0–12.0	59	8.7	7.1–10.3	59	19.2	16.7–21.7	185	12.4	11.2–13.8	244	14.0	12.8–15.2
Males	27	6.2	5.1–8.3	27	6.3	4.7–8.0	27	12.9	10.5–15.3	153	7.9	7.0–8.8	180	8.6	7.8–9.6
Females	28	14.5	11.9–17.2	28	9.7	7.5–12.0	28	24.2	21.2–27.5	156	15.7	14.2–17.4	184	17.0	15.5–18.4
TTH															
Both genders	33	19.4	15.3–24.1	33	10.0	7.9–12.6	33	29.4	24.4–34.3	77	24.5	20.7–29.0	110	26.0	22.9–29.6
Males	12	18.1	11.4–25.4	12	8.6	4.6–13.3	12	26.8	20.6–33.5	52	22.6	17.7–28.4	64	23.4	19.1–28.0
Females	12	22.4	13.2–32.9	12	9.4	5.3–14.6	12	31.8	23.6–40.4	54	26.1	21.0–31.7	66	27.1	22.7–32.2

*N* number of studies, *TTH* tension-type headache, *CI* confidence interval

**Table 5:** Prevalences (%) of probable migraine and probable TTH by age and gender, and as proportions (%) of all (probable + definite) migraine and TTH

Gender/age (years)	Probable migraine			Probable TTH			Total migraine (pMig+dMig)			Total TTH (pTTH+dTTH)			pMig as % of total migraine	pTTH as % of total TTH
	N	Mean	95% CI	N	Mean	95% CI	N	Mean	95% CI	N	Mean	95% CI		
Both genders														
0–9	0	–	–	0	–	–	–	–	–	0	–	–		
10–19	13	10.5	7.0–14.4	8	14.0	9.5–19.8	13	20.3	14.3–26.4	8	29.2	19.3–45.1	52	48
20–64	44	8.4	6.6–10.2	24	9.0	6.5–11.9	44	19.4	16.5–22.5	24	29.4	24.6–34.4	43	31
≥65	2	3.1	–	2	6.6	4.3–8.9	2	6.8	–	2	20.6	–	46	32
All ages	59	8.7	7.1–10.4	34	10.0	7.7–12.6	59	19.2	16.7–21.7	34	28.9	24.4–34.2	45	35
Males														
0–9	0	–	–	0	–	–	0	–	–	0	–	–		
10–19	5	9.1	5.4–12.9	3	9.8	–	5	18.0	12.6–24.0	3	18.9	–	51	52
20–64	21	5.7	4.0–7.7	8	8.8	2.9–15.6	21	12.1	9.6–14.6	8	29.6	19.7–38.0	47	30
≥65	1	3.7	–	1	4.3	–	1	5.1	–	1	28.1	–	73	15
All ages	27	6.3	4.7–8.0	12	8.6	4.6–13.3	27	12.9	10.5–15.3	12	26.8	20.6–33.5	49	32
Females														
0–9	0	–	–	0	–	–	0	–	–	0	–	–		
10–19	5	13.4	7.8–18.5	3	11.3	7.2–16.9	5	26.0	20.0–34.2	3	21.7	21.2–22.4	52	52
20–64	22	8.9	6.6–11.2	8	9.4	3.8–16.6	22	24.2	20.7–27.9	8	35.1	22.8–47.8	37	27
≥65	1	8.0	–	1	4.3	–	1	16.0	–	1	36.3	–	50	12
All ages	28	9.7	7.6–11.7	12	9.4	5.3–14.6	28	24.2	21.2–27.4	12	31.8	23.6–40.4	40	30

*N* number of studies, *TTH* tension-type headache, *CI* confidence interval (not given when N ≤ 5), *pMig* probable migraine, *dMig* definite migraine, *pTTH* probable TTH, *dTTH* definite TTH

**Table 6:** Bivariate analyses of active headache disorders according to methodological aspects of studies

		Headache (183 studies)		Migraine (244 studies)		TTH (110 studies)		H15+ (59 studies)	
**Correlations**		**r**	**p**	**r**	**p**	**r**	**p**	**r**	**p**
Year of publication		0.09	0.22	0.30	< 0.0005	−0.03	0.8	0.20	0.14
Number (ln transformed) of participants		−0.27	< 0.001	−0.26	< 0.001	−0.17	0.07	−0.33	0.01
Participating proportion (9 categories)		0.11	0.15	0.02	0.7	−0.11	0.2	−0.02	0.9
Age (mid-range value) (years)		−0.14	0.05	0.03	0.6	0.13	0.2	0.14	0.3
Summed quality score		0.03	0.7	−0.02	0.8	− 0.02	0.8	0.00	1.0
**Means of reported prevalences (%)**		**Mean (SD, N)**		**Mean (SD, N)**	**p**	**Mean (SD, N)**	**p**	**Mean (SD, N)**	**p**
Population^a^	General/schools	51.9 (22.7, 142)	1.0	13.5 (8.4, 177)	0.2	26.5 (17.0, 85)	0.6	4.8 (3.1, 54)	0.2
	Selected	52.2 (22.7, 41)		15.5 (11.1, 66)		24.4 (21.1, 25)		3.0 (2.1, 5)	
Health-care personnel^b^	No	50.2 (25.4, 33)	0.3	13.4 (9.1, 52)	0.03	23.3 (22.9, 19)	0.7	3.0 (2.1, 5)	–
	Yes	60.0 (22.9, 8)		23.0 (14.7, 15)		27.8 (15.2, 6)		–	
Sampling method^c^	Random	54.7 (23.2, 44)	0.4	12.8 (8.1, 184)	0.002	25.7 (18.6, 83)	0.8	4.5 (3.2, 51)	0.4
	Not random	51.1 (23.1, 139)		18.0 (11.4, 60)		26.8 (16.0, 27)		5.5 (2.1, 8)	
Mode of engagement^d^	Telephone/face-to face	50.8 (23.6, 95)	0.5	13.1 (8.8, 136)	0.07	26.3 (18.7, 66)	0.8	5.5 (3.5, 32)	0.02
	Questionnaire	53.2 (22.6, 88)		15.3 (9.8, 108)		25.5 (17.0, 44)		3.6 (2.0, 27)	
Validation of diagnostic method^e^	Yes	51.2 (24.3, 93)	0.7	14.3 (10.3, 107)	0.7	25.6 (18.0, 56)	0.8	4.6 (3.2, 41)	0.9
	No	52.8 (22.1, 90)		13.8 (8.5, 137)		26.(18.0, 54)		4.7 (2.6, 18)	
dMig and pMig^f^	Yes	55.4 (20.8, 46)	0.3	19.2 (9.9, 59)	< 0.001	27.6 (15.9, 41)	0.5	5.6 (3.9, 22)	0.1
	No	50.8 (23.9, 137)		12.4 (8.5, 185)		25.0 (19.1, 69)		4.1 (2.2, 37)	
dTTH and pTTH^g^	Yes	61.5 (17.8, 29)	0.02	20.0 (10.3, 32)	< 0.001	29.5 (13.7, 33)	0.2	6.2 (4.1, 18)	0.04
	No	50.2 (23.7, 154)		13.1 (8.8, 212)		24.5 (19.4, 77)		3.9 (2.2,41)	
Number of conditions^h^	Only headaches	52.9 (23.0, 173)	0.02	14.3 (9.3, 235)	0.04	26.0 (18.0, 110)	–	4.6 (3.0, 59)	–
	Including other conditions	35.2 (20.4, 10)		7.8 (5.6, 9)		–			
Type of screening question	Neutral	61.8 (22.0, 58)	< 0.0005	17.1 (9.3, 65)	0.006	31.9 (22.5, 42)	0.02	5.0 (2.1, 24)	0.8
	Unknown	50.8 (23.7, 71)		12.9 (10.0, 111)		23.0 (14.2, 41)		4.3 (2.4, 16)	
	Severity qualification	41.8 (19.1, 52)		12.7 (6.6, 66)		20.9 (12.1, 26)		4.4 (4.5, 18)	

*TTH* tension-type headache, *H15+* headache on ≥15 days/month, *r* Pearson’s r, *p* two-tailed *p*-value (Pearson’s r, independent-samples Student’s t-test, or one-way ANOVA for screening question), *SD* standard deviation, *N* number of studies

^a^General population, community-based sample or obligatory schools versus selected populations (among workplace employees, college students, health-plan members etc)

^b^Among selected populations: medical students, hospital staff or neurologists versus other selected populations

^c^Random sampling versus non-random sampling or failure to obtain random sample

^d^Telephone or face-to face interview versus self-administered questionnaire or not specified

^e^Effort at validating versus none or unstipulated

^f^Studies distinguishing between dMig and pMig and reporting both versus those not

^g^Studies distinguishing between dTTH and pTTH and reporting both versus those not

^h^Studies restricted to headache (or migraine, TTH etc) *versus* studies investigating multiple other disorders (neurological, other pain conditions etc)

We used multiple linear regression (MLR) analyses to explore associations between prevalence (dependent variable) and study variables (geography, publication year, and the variables related to method and quality described above, reporting R^2^ (“Goodness-of-fit”, the proportion of the variation explained by the whole model) and, for each individual variable of significance, β-standardised (the direction and strength of its correlation with the dependent variable [prevalence]) and the square of the semipartial correlation (SPC^sqr^: the proportion of the variability explained by that variable alone). While all variables used in the bivariate analyses (listed in Table 6) were entered into the MLR models for all headache disorders, this was an exploratory exercise; therefore, a stepwise method excluded variables when F > 0.06 and included those when F ≤ 0.05. Although MLR is quite robust with regard to non-normal distributions, the variable “number of participants” was very skewed, most samples being in the hundreds or low thousands but some very large (tens or hundreds of thousands). Therefore, this variable was logarithmically transformed (ln). For the seven GBD superregions (High Income countries [HI], Central and Eastern Europe and Central Asia [CEEAO], Latin America and Caribbean [LAC], North Africa and Middle East [NAME], South Asia [SA], SouthEast and East Asia and Oceania [SEEAO], and Sub-Saharan Africa [SSA]), we created dichotomized dummy variables using HI superregion, which had more studies than all others combined, as the reference.

Since many variables were not normally distributed, we undertook a sensitivity analysis using non-parametric methods (correlations with Spearman’s rho and comparisons of group differences with Mann-Whithney U-test for two groups or Kruskall Wallis Test for several groups). No good non-parametric method exists for MLR-analysis. All dependent variables were checked for normality (Shapiro-Wilk [SW] test), both before and after attempting to normalize them by ln-transformation and removal of outliers (outside 1.5 *interquartile range). In the MLR analysis, multicollinearity was evaluated by Variance Inflation Factor (VIF, < 4.0), and residuals in all models were checked for normal (histograms and P-P plots) and equal distribution (homoscedasticity with scatter plots) (see Additional file 2).

All analyses were performed with IBM SPSS (version 27). We considered *p* < 0.05 (two-sided) to indicate significance. No adjustments for multiple testing was performed since testing was done only for exploratory purposes.

## Results

### Studies

A total of 357 published papers were identified that were judged relevant to this review (Additional file 1). The table has more than 357 rows because some papers were referred to multiple times, reporting data from two or more studies performed in different years, in different countries or places, with different methods in the same place, or with different timeframes. Some rows have more than one reference because data from the same study but on different headache types (e.g. migraine and TTH) were reported in multiple publications.

The number of publications from each GBD superregion varied considerably: 183 from HI, 25 from CEEAO, 44 from LAC, 42 from NAME, 11 from SA, 24 from SEEAO and 27 from SSA. It should be noted that all GBD superregions are continuous geographical areas except HI, which includes countries in Australasia, Asia Pacific, Western Europe, North America and Southern Latin America.

### Descriptive overview

Many studies were deficient in their reporting, hindering our analyses.

For age, many gave data for different groups (e.g., 5-, 10- or 20-year categories), some but not all did so for each gender, some did so for only one of the headache types and some did so for each type. All studies on very specific age groups (e.g., children, adolescents, elderly) gave age ranges, these being the source of all mid-range values below 10, from 10 to 19 or above 65 years. Most of the many studies with mid-range values between 20 and 64 years were conducted in adults (young and middle-aged), but some included all ages (5–99 years). Some, however, were restricted to the actively working population (18–65 or 20–65 years), and some to narrow age ranges (e.g., a single-year cohort of 40 years, or 20–35, or 45–64 years). Hence, our study was not well able to show age-specific effects: we could relate prevalences only to four ranges of mid-range values (< 10, 10–19, 20–64, ≥65 years).

With regard to timeframe, many studies reported 1-year prevalence defined appropriately (a positive response to “have you had headache during the last year?”), but a number used shorter timeframes, often with the assumption that questions about headache during the last year were sufficiently answered by considering only the last 3–6 months. Others asked whether participants had headache “currently”, or specified no timeframe (e.g., “do you suffer from headaches?”), but clearly implied the present rather than throughout the lifetime. Studies with even shorter timeframes (usually 24 h, “headache yesterday”, or “now”) always defined the timeframe when questioning participants.

Many studies did not describe the screening question. As a group, they probably included a mixture of screening questions (neutral or qualified).

Many studies were silent on the distinction between definite and probable diagnoses, and may or may not have included the latter.

### Prevalence of an active headache disorder

Globally, an active headache disorder of any type was present in 52.0% of the populations studied (males 44.4%, females 57.8%), migraine in 14.0% (males 8.6%, females 17.0%) and TTH in 26.0% (males 23.4%, females 27.1%) (Table 1). The overall value is not always the mean of the values for males and females because some studies were restricted to one gender while others gave only the overall value. H15+ was reported by 4.6% (males 2.9%, females 6.0%). All these data are detailed in Table 1 by gender and age. Headache prevalences were reportedly similar in all age groups in both genders (overlapping CIs). On the other hand, studies with mid-range age values below 10 or above 65 years reported lower migraine prevalences in both males and females, and studies with values below 10 years reported lower TTH prevalences in both genders. H15+ estimates were lower among females with mid-range age values of 10–19 years and among males. Mean values were approximately 20% higher than the medians, for migraine (mean 14.0% versus median 11.6%), TTH (26.0% versus 21.4%) and H15+ (4.6% versus 3.6%), but not for all headache (52% versus 53.5%) (Table 1).

Table 2 shows prevalences by geographical area (GBD superregions). All headache reportedly varied between 38.7% (SEEAO) and 60.5% (NAME), migraine between 9.0% (SEEAO) and 22.2% (SA), TTH between 11.1% (SEEAO) and 33.1% (SA), and H15+ between 2.8% (SEEAO) and 6.9% (SA).

For comparison, Table 2 also provides the latest available estimates from GBD [13]. For all headache, these were generally somewhat lower than our findings, globally and in several of the GBD superregions (with non-overlapping 95% CIs globally and in HI, LAC, NAME and SSA). This was despite that GBD estimates for migraine and TTH were generally higher. GBD does not give separate data on H15+, although more recent iterations subsume medication-overuse headache within migraine or TTH according to the proportions expected to arise from each.

Table 2 also shows the percentages of the global population living in each GBD superregion. Adjusting for these (not shown in the table), the global prevalence of headache was 45.6%, of migraine 13.6%, of TTH 21.1%, and of H15+ 4.7%. This simple adjustment does not allow CIs to be calculated, and is valid only if estimates per superregion are correctly representative for the whole of each superregion. The adjusted values are more similar to those of GBD for all headache, but not for migraine or TTH.

In studies estimating prevalences of an active headache disorder and of specific headache types, there were clear positive correlations between them: for headache with migraine (r = 0.46, *p* < 0.01, 142 studies), with TTH (r = 0.48, *p* < 0.01, 84 studies) and with H15+ (r = 0.45, *p* < 0.01, 42 studies), for migraine with TTH (0.36, *p* < 0.0000, 105 studies) and with H15+ (0.45, *p* < 0.01, 43 studies), and for TTH with H15+ (0.37, *p* = 0.2, 43 studies).

### Prevalence related to methodological and other study variables

#### Timeframe

Table 3 shows that reported prevalences varied with timeframe. Excepting 1-day estimates, this was mostly with widely overlapping 95% CIs: of all headache from 37.2% (“current”) to 66.6% (lifetime), of migraine from 11.1% (6-month) to 17.5% (lifetime), of TTH from 20.2% (3-month) to 40.6% (lifetime), and of H15+ from 2.7% (3-month) to 6.0% (6-month).

As to 1-day prevalence, 15.8% of the global population reportedly had headache on any particular day (7.0% migraine, 8.7% TTH and 2.5% H15+). Many of the studies reporting 1-day prevalence (as headache yesterday) did not attempt to diagnose headache type.

#### Definite and probable (or unspecified) diagnoses

Table 4 makes the distinction between definite and probable diagnoses of migraine (reported in 59 studies) or of TTH (33 studies). Mean reported prevalences for migraine were 10.5% for dMig and 8.7% for pMig, the sum being 19.2%. In studies providing gender-specific estimates, these were 6.2 and 6.3% for males, 14.5% and 9.7% for females. For TTH they were 19.4% for dTTH and 10.0% for pTTH (sum 29.4%; males 18.1% and 8.6%, females 22.4% and 9.4%).

Table 4 also provides mean estimates from the 185 studies not making or not specifying the distinction. These studies yielded a prevalence of migraine (12.4%) that was somewhat higher than that of dMig (10.5%) from the 59 studies that did distinguish, but lower than their sum of dMig and pMig (19.2%). The combination of all studies gave the 14.0% global estimate reported above. Similarly for TTH, the mean reported prevalence of unspecified TTH (24.5%) was between that of dTTH (19.4%) and sum of dTTH and pTTH (29.4%), with 26.0% from the combination of all studies.

Table 5 relates definite and probable diagnoses to gender and age. In the contributory studies, pMig constituted 45% (8.7/19.2*100) of all migraine (pMig+dMig), somewhat more among males (49%) than females (40%), and among adolescents 10–19 years of age (52%) than adults aged 20–64 years (43%) or ≥ 65 years (46%). For TTH, proportions of pTTH were somewhat lower (overall 35%, males 32%, females 30%), but markedly higher among adolescents (48%) than among the two adult groups (31% and 32%).

#### Bivariate analyses

Table 6 shows bivariate correlations of active headache disorders with continuous and ordinal variables (publication year, number of participants [ln-transformed], participating proportion and summed quality score), as well as comparing means of reported prevalences according to various categorical variables. Migraine prevalence was positively associated with publication year (r = 0.30). Headache (r = − 0.27), migraine (r = − 0.26) and H15+ (r = − 0.33) were negatively associated with number of participants, TTH showing a trend in the same direction (r = − 0.17). There were no significant associations with summed quality score (*p* ≥ 0.7). As to the population of interest, estimates for migraine from general populations, defined regions or schools were not significantly different from those from selected sub-populations, although, among the latter, estimates were higher in health-care personnel than others (23.0% versus 13.4%, *p* = 0.030). With regard to sampling method (random versus not or failed attempt to secure randomness), estimates for migraine were significantly lower in studies with random sampling (12.8% versus 18.0%, *p* = 0.002), but this was not so for all headache or the other headache types (*p* ≥ 0.4). Mode of engagement (telephone or face-to-face interview by untrained, trained or specialized interviewer versus self-administered questionnaire or unknown) influenced estimates for H15+ (5.5% versus 3.6%, *p* = 0.02), but not those for the other headache types. There was no evident influence of validation of the diagnostic method (*p* ≥ 0.7). Distinction or not between pMig and dMig made a difference for migraine (19.2% versus 12.4%), and distinction between dTTH and pTTH did so for all headache (61.5% versus 50.2%), migraine (20.0% versus 13.1%), and H15+ (6.2% versus 3.9%). Studies restricted to headache and not including other conditions reported higher estimates for all headache (52.9% versus 35.2%, p = 0.02) and for migraine (14.3% versus 7.8%, *p* = 0.04). Neutral screening questions were associated with higher estimates for all headache, migraine and TTH than were unspecified questions (intermediate) and those implying some level of severity, frequency or presumed causation.

#### Multiple linear regression (MLR)

None of the dependent variables (headache, migraine, TTH and HA15+, in both genders combined) were normally distributed (*p* ≤ 0.01, SW). Normalizing the dependent variables (by ln- transformation and removal of outliers, see Statistics) was successful for three (migraine, TTH and HA15+; *p* > 0.14), but not for headache (*p* < 0.01). No significant collinearity was found in any of the models (VIF < 1.40). For distribution of residuals, see Additional file 2 (Histograms, P-P plots and Scatterplots).

Table 7 shows the MLR results for original (Model 1) and normalized (Model 2) dependent variables. Model 2 should be the more robust. For TTH, the two models are identical with regard to the variables included and directions of the associations, and for migraine, the principal difference is that one superregion (NAME) is not included in Model 2. For H15+, the two models are very different, probably showing that the robustness of MLR models declines with smaller numbers of observations, particularly when the dependent variable is not normally distributed. For headache, two methodological variables and one superregion occur in both models.

**Table 7:** Multiple linear regression with stepwise inclusion of variables (HI superregion serves as reference for other superregions)

**Model 1: Original data set**	**Headache,** ***N*** **= 181** **(5 variables)**		**Migraine,** ***N*** **= 242** **(8 variables)**		**TTH,** ***N*** **= 109** **(4 variables)**		**H15+,** ***N*** **= 58** **(2 variables)**	
Whole model	F = 10.3, *p* < 0.001, R^2^ adj: 0.208		F = 13.9, *p* < 0.001, R^2^ adj: 0.299		F = 6.9, *p* < 0.001, R^2^ adj: 0.181		F = 6.6, *p* = 0.003, R^2^ adj: 0.165	
**Variables with significance**	**β standardised**	**SPC**^**sqr**^	**β standardised**	**SPC**^**sqr**^	**β standardised**	**SPC**^**sqr**^	**β standardised**	**SPC**^**sqr**^
Year of publication			0.258	0.060				
Number (ln transformed) of participants	−0.207	0.040	− 0.146	0.018				
dMig and pMig			0.269	0.064				
dTTH and pTTH							0.321	0.102
Health-care personnel			0.173	0.022				
Sampling method			−0.169	0.022				
Type of screening question	−0.307	0.090			−0.285	0.080		
Number of conditions	−0.162	0.026						
Mode of engagement							0.281	0.078
SSA superregion	−0.148	0.021	−0.173	0.028	−0.232	0.052		
SEEAO superregion	−0.171	0.029	−0.136	0.018	−0.294	0.084		
NAME superregion			−0.113	0.011	−0.231	0.051		
**Model 2: ln transformed dependent variable and outliers (±1.5 interquartile range) removed**	**Headache,** ***N*** **= 176** **(4 variables)**		**Migraine,** ***N*** **= 238** **(7 variables)**		**TTH,** ***N*** **= 103** **(4 variables)**		**H15+,** ***N*** **= 57** **(2 variables)**	
Whole model	F = 11.3 *p* < 0.001, R^2^ adj: 0.191		F = 13.6, *p* < 0.001, R^2^ adj: 0.271		F = 5.5, *p* < 0.001, R^2^adj:0.150		F = 6.5, *p* = 0.003, R^2^ adj: 0.164	
**Variables with significance**	**β standardised**	**SPC**^**sqr**^	**β standardised**	**SPC**^**sqr**^	**β standardised**	**SPC**^**sqr**^	**β standardised**	**SPC**^**sqr**^
Year of publication			0.259	0.064				
Number (ln transformed) of participants	−0.186	0.032	−0.137	0.016			−0.354	0.125
dMig and pMig			0.291	0.078				
dTTH and pTTH	0.166	0.027						
Health-care personnel			0.133	0.013				
Sampling method			−0.169	0.022				
Type of screening question	−0.277	0.072			−0.233	0.054		
SEEAO superregion			−0.110	0.012	−0.206	0.058	−0.250	0.062
SSA superregion	−0.207	0.042	−0.180	0.031	−0.245	0.042		
NAME superregion					−0.264	0.068		

R^2^ adj (Adjusted R^2^): The proportion of variability explained by the whole model (“Goodness of fit”), adjusted for overestimation caused by the number of effects

β-standardised: The direction and strength of its correlation with the dependent prevalence variable

SPC^sqr^: the proportion of the variability explained by that variable alone

Overall, a relatively small proportion of the variations in reported headache prevalence was explained by these variables, ranging from 29.9% (whole model R^2^) in migraine to 16.5% in H15+ (Model 1), and from 27.1% (migraine) to 15.0% (H15+, Model 2).

For all headache the methodological variables included in both models were the type of screening question (SPC^sqr^, explaining 9.0% in Model 1 and 7.2% in Model 2), and the number of participants in the study (4.0% and 3.2%, negatively correlated). Whether the study investigated only headache or included other disorders was included in Model 1(2.6%, lower estimates in the latter), and whether it included both dTTH and pTTH in Model 2 (2.7% more when it did). For migraine, the most important factor was distinction between dMig and pMig (6.4% and 7.8% in the two models), followed by publication year (6.0% and 6.4%, positively correlated), sampling method (2.2% in both models lower estimates when random), selection of health-care personnel (2.2% and 1.3% higher estimates) and number of participants (1.8% and 1.6%, negatively correlated). For TTH, the only methodological factor was the type of screening question (8.0% and 5.4%). For both migraine and TTH, certain world superregions (SSA, SEEAO, and NAME for TTH) had lower estimates than the reference (HI superregion). For H15+, the only methodological variable included in Model 2 was number of participants (12.5%, negatively correlated). SEEAO superregion had lower prevalence (6.2%, Model 2) than HI. In Model 1 were also included whether the study distinguished between dTTH and pTTH (10.2%, higher when they did) and mode of engagement (7.8%, higher with telephone/face-to-face than with unsupervised questionnaire).

#### Sensitivity analyses

Correlations between the different headache types were very similar using a non-parametric method (Spearman’s rho, 0.36–0.49, *p* < 0.01 for all). In the bivariate analyses (Table 6), two of the 56 *p*-values became non-significant with non-parametric methods (headache correlated with age, and TTH related to dTTH or pTTH) while four became significant (migraine related to mode of engagement, TTH to participating proportion, age and screening question). Thirteen analyses remained significant and 37 nonsignificant with both methods.

In addition, a second MLR analysis (not shown), with GBD superregions omitted from the modelling, found exactly the same methodological variables to be significant.

## Discussion

### General comments

This review has many of the properties of a systematic review (e.g., specification of search terms and databases, evaluation of heterogeneity), and even of a meta-analysis (summary measures), without conforming fully with accepted methodology for the former (see Methods: Literature search and data extraction). Such reviews, following published guidelines, are usually made to evaluate effects of interventions. While many of the same principles remain relevant to other types of data, guidance and reporting standards for systematic reviews of prevalences are lacking [14]. Hence, we conceived this as a narrative rather than a systematic review of publications reporting headache prevalences, updating our previous review of 2007 [5].

In comparison with our previous review, we found an apparent increase in prevalence of migraine but not those of other headache types. We also found wide geographical variations, as did the 2007 review. Estimates for migraine were very high in populations with very good knowledge and interest in the disorder (e.g., neurologists, and other health personnel). While estimates were influenced to varying degrees by several methodological factors (nature of the screening question, number of conditions investigated, sampling method, number of participants, how patients were engaged and not least – for migraine and TTH – how ICHD criteria were applied with regard to definite and/or probable diagnoses), many of these influences were not significant. Since the review included studies of all populations of interest other than clinical populations, and extracted many data on methodological and other aspects of each study, we believe it provides a strong basis for examination of these influences, which we discuss in the following sections. Meanwhile we note that our prevalence estimates in this review derive from studies varying with regard to these factors, so that it remains in doubt whether the observed differences over time and between geographical regions are real.

Estimated medians for the different headache types were approximately 20% lower than the means, whereas, for headache overall, the median estimate was 3% higher. It could be argued that medians, uninfluenced by outliers with very high prevalences, were more correct and conservative summary measures. On the other hand, we had no a priori reason to believe that studies with high estimates were less correct or representative for the population at large than those with low estimates. In many ways, studies finding high prevalences might have been methodologically better (neutral screening question, considering both probable and definite diagnoses, face-to face interviews, etc). Therefore, we based estimates on the means.

The sensitivity analysis showed that the bivariate analyses were little influenced by statistical method (parametric or non-parametric).

### Relation to case definition: “active headache disorder”

Case definition is of over-riding importance, and likely to be the single most influential factor in any enquiry dependent on prevalence estimation (including burden estimation) [4, 15]. For headache disorders, case definition revolves around timeframe, diagnostic criteria and how the latter are applied. Since 1988, use of ICHD criteria has become mandatory in the absence of acceptable alternatives [4], but, from a pragmatic perspective, some flexibility in their application to epidemiological enquiry is invariably necessary. Empirically, we found different approaches towards this flexibility among the reviewed studies, and these were not always described.

As to timeframe, we might have restricted this review to studies reporting 1-year prevalence, but we believe the ICHD definition of “active headache disorder” [9] does not exclude studies reporting “current”, 6- or 3-month prevalences (see Methods: Timeframe of headache). These latter timeframes were more restrictive, and therefore given to underestimation, as appears from Tables 1 and 3 (although only of episodic headache, by exclusion of those with low-frequency episodes). On the other hand, those reporting lifetime prevalences would tend to give higher estimates through inclusion of “non-active” headache. Those in which timeframe was unknown (not specified) might go either way. Overall, our estimates may be somewhat conservative because we subsumed shorter timeframes into our case definition of “active headache disorder”, but this approach is validated by the fact that, for all timeframes subsumed (1-year, 6-month, 3-month, “current” and unknown), 95% CIs were widely overlapping (except for H15+, with only 3 studies).

As to ICHD criteria and their application, we might also have restricted our case definitions for migraine and TTH to definite diagnoses (meeting all ICHD criteria). This would have had a large influence: probable diagnoses accounted for > 40% of total migraine and > 30% of total TTH. The persuasive arguments for including probable diagnoses have been set out before [4]. In clinic, probable diagnoses are, or should be, confirmed or refuted during follow-up, and have utility in allowing management plans to be set meanwhile. This utility is lacking in epidemiological studies, with subsequent enquiry rarely possible, but on the one hand excluding probable cases would clearly provide a partial account of headache prevalence while, on the other, probable cases are probably what they appear to be [4]. Most studies did not explicitly state how ICHD criteria were applied, and, while some gave estimates for both definite and probable cases, a few reported only the sum of these. We would, therefore, have excluded the majority of studies had we limited the review in this way, while grossly underestimating true prevalences, as evidenced for migraine in particular in Tables 4 and 6. In the MLR analyses (Table 7), this was the factor with the highest SPC^sqr^, alone explaining 6.4% (Model 1) to 7.8% (Model 2) of the variation in migraine prevalence.

In summary, with regard to case definition, we believe that best estimates from available data are made by including studies with various timeframes, and including definite and probable (or unspecified) diagnoses. These inclusions allow many more studies to be taken into account, providing data from countries and regions that otherwise would have few or none. This is not to say that future studies should not give due regard to these very important methodological issues.

### Relation to publication year and geography

Unlike case definition, there were no obvious a priori reasons for expecting these factors to be significantly influential. Although geographical variation of course implies genetic, environmental, cultural, economic, lifestyle and general health variations, among these only dwelling altitude has clearly demonstrated impact, and this only in Nepal [16]. Urban/rural divide and relative wealth or poverty are weakly related to headache prevalence in some studies, not always in the same direction. Population age may be a factor: age itself is strongly associated with prevalence of all headache types, and some national populations are relatively young in comparison with the global mean (but, it should be noted, this factor is not independent of population poverty or general health).

Compared to our previous summary of prevalence data from 2007 [5], this review has found increased prevalences of active headache (from 46% to 52%), of migraine (from 11% to 14%) and of H15+ (from 3% to 4.6%) but decreased prevalence of TTH (from 42% to 26%). However, in both bivariate and MLR analyses, association between prevalence and publication year was significant only for migraine. An increase in prevalence was also found in some [17, 18] but not all [19, 20] studies performed in the same populations at different time points. As to geography, mean reported prevalences of all headache types varied considerably, albeit with wide and mostly overlapping CIs. Compared with HI superregion, lower prevalences were estimated for all headache and migraine in SSA and SEEAO, and for TTH in NAME.

However, it is uncertain whether or to what extent these differences over time and place are real: overall, the MLR analyses show that the present models explain relatively little of the large variations in prevalence estimates between studies (for migraine less than 30%, and even less for other headache types, possibly because of fewer studies). There are many other aspects of methodology (see below), some that are difficult to identify and impossible to quantify but which are likely or certain to have varied between studies in different times and places, and perhaps been influential. Standardisation according to accepted guidelines will help in future studies [4], but these factors, and the uncertainties they generate, will persist in the large corpus of historical studies from mostly HI countries.

The relatively few studies from the other GBD superregions are vulnerable to incidental findings. Therefore, with the large and unexplained variations, it may be risky (less accurate) to adjust the estimate of global prevalence for proportions of the global population living in each region. For example, SA and SEEAO have half the world’s population but only 9% (17/180) of headache prevalence studies. Better estimates of global prevalences may be obtained by considering all studies as a way of cluster sampling the world, although adjustment could and should still be made for the huge oversampling from HI countries. Arguably, the rich variety of methods used make the overall estimates more resistant to yet unknown sources of variation.

### Relation to population of interest

This review included studies of all populations of interest other than clinical (patient) populations. In contrast, the review from 2007 [5] included only studies with > 500 participants on whole populations or representative samples of these within specified age ranges in communities, towns or countries, together with studies performed in schools. This means we took account here of studies on smaller and more selected populations (e.g., among employees of a company or factory, hospital staff, university students, neurologists, ethnic minorities, inhabitants of a slum area, a tribe, a monastery, etc). Such populations were likely to differ from the general population in the country in question (i.e., more or less healthy, poorer or richer, more or less educated, etc) but, by including these, we gathered data from more diverse settings and many more countries. Again, we believe this approach results in a more representative sample of the world’s population, while, with one exception (see below), neither bivariate nor MLR analyses found significant differences between estimates from selected or more general populations.

The exception was studies on health-care personnel (medical students, hospital staff, neurologists). These yielded significantly higher prevalence estimates for migraine in both bivariate and MLR analyses. The studies on neurologists are an interesting example: three studies from USA [21], France [22] and Norway [23], considering either 1-year prevalence or “current” headache (unknown timeframe), found a mean prevalence of 42.2%. It has been argued that this very high prevalence is a true finding because neurologists are expected to diagnose themselves with very high sensitivity and specificity [23]. This is not to say it reflects the true population prevalence: neurologists are in many ways a highly selected and unrepresentative group.

### Relation to age and gender

This review mostly confirmed earlier studies on age and gender distributions (Tables 1, 4 and 5). Headache in general was most prevalent in the group with mid-range age values between 10 and 19 years. Overall, and in females, migraine was most prevalent between 20 and 64 years, whereas in males it was somewhat higher from 10 to 19, although with overlapping 95% CIs. TTH was most prevalent in the 20–64 years age group for both genders (except for the single study in those above 64 years), as was H15+. The age group 20–64 years is, of course, a very broad category, and this grouping only (and imprecisely) distinguishes adults from children and adolescents on the one hand and from the elderly on the other. However, the data did not allow a more refined analysis.

All headache types were more common in females, most markedly for migraine (17.0% versus 8.6%) and H15+ (6.0% versus 2.9%), the more disabling types, while the difference was small and not significant in TTH. Gender differences were least in the youngest age groups (Table 1).

The relationships between age and prevalences of dMig and pMig, and of dTTH and pTTH (Table 5), are worthy of comment. The proportion of pMig to total migraine was higher in the age group 10–19 years than in those who were older, and also tended to be higher among males. This suggests that ICHD migraine criteria are somewhat less fitting for children and adolescents than for adults, and perhaps for males than for females. The proportion of pTTH to total TTH was equal between genders, but again much higher in young people. In the latter group, a high prevalence has been noted in several studies of headaches unclassifiable within ICHD, meeting the criteria for neither definite nor probable migraine or TTH, and described as “undifferentiated headache” [24].

### Comparisons with GBD2019

For migraine and TTH, our estimates of global prevalence and those of GBD2019 had widely overlapping CIs (Table 2), but for all headache our estimate (52.0% [48.9–55.4]) was markedly higher than that of GBD2019 (35.0% [32.3–37.7]). Possibly, this is because, in GBD, ‘“all headache’” is the sum of migraine and TTH, whereas our estimates are based on studies among which the majority asked about headache in general, therefore also including other headache types. However, it is puzzling that in GBD2019 [13] (and earlier versions [25, 26]) total headache prevalence is markedly lower than the sum of migraine and TTH prevalences. The explanation does not, apparently, lie in corrections for comorbidity of the two headache types.

GBD estimates are based on sophisticated mathematical modelling [2], which combines methods of meta-analysis with regression techniques (“meta-regression”). It not only adjusts for gender and age composition of populations, for comorbidity and for some methodological variables, but also takes account of multiple geographical levels (country, region, superregion, global) and year of study (“borrowing strength over space and time”) [27]. These are smoothing methods, whereby GBD is able to make estimates for all gender- and age-groups, for smaller regions, individual countries and even parts of countries. GBD estimates are more even across regions and time periods than ours: we can make reasonable estimates for global prevalences over a long time span but, for countries, smaller regions and defined time periods, in the absence of data specific to these, GBD methods achieve what we cannot. This is not a comment on which estimates might be truer in populations from whom data have been directly derived in one or more methodologically sound studies.

### Influences of methodological factors

In the MLR analyses, publication year appeared important as a factor explaining variation in migraine prevalence estimates (6.4% of variation in Model 2, higher estimates associated with more recent publication), but it played no role in other headache types. The apparent increase in migraine prevalence over time may be real, perhaps related to environmental, physical, behavioural or psychological changes, but more probably it has to do with methodological developments over the years, leading to better techniques of access and engagement and improved diagnostic instruments, both likely to enhance case ascertainment. The positive correlations between prevalence estimates of all diagnoses indicate that case ascertainment is an important factor also for the other headache types, and for headache in general. Certainly they speak against a hypothesis that, if one diagnosis is made more often, it is at the expense of another. It may also be that better knowledge and greater awareness of a condition lower the thresholds for reporting relevant symptoms. That knowledge of migraine and its diagnosis can affect estimates is supported by the findings of higher migraine prevalence among medical personnel, although these are, largely, findings specific to the high end of the spectrum of knowledge. There has been no similar focus on other common headache types.

The negative association of prevalence estimates of all headache, migraine and H15+ with number of study participants (Model 2: 3.2%, 1.6% and 12.5% of variations respectively) may indicate that smaller studies can afford more sensitive methods (personal interview, face to face or by phone) to detect cases. Alternatively, it may be caused by greater selection bias in small studies. This would explain the absence of similar effect on estimates for TTH, since people whose lives are less impacted by a disease have less interest in surveys enquiring into it. That more random sampling – generally more methodologically rigorous – was negatively associated with migraine prevalence estimates may similarly be explained by reduced selection bias.

It seems obvious that studies including pMig would report markedly higher estimates of migraine prevalence, since > 40% of total migraine prevalence in these studies was attributed to pMig. Including pTTH did not significantly affect estimates of TTH, perhaps because cases that might have been classified as TTH met criteria for pMig, counterbalancing the otherwise expected increase. That inclusion of pTTH was associated with a higher prevalence of all headache (Model 2) suggests this captured cases, probably of mild headache, that were otherwise overlooked.

It also seems obvious that a screening question setting some threshold of severity, and therefore excluding milder cases, would lead to lower prevalence estimates of all headache (9.0% and 7.2% of variation) and of TTH, a less severe headache type (8.0% and 5.4% of variation), but not of migraine or H15 + .

### Lifetime and 1-day prevalences

Lifetime prevalences are higher than those of active headache disorders, except for H15+, but information about headache earlier in life is not relevant to estimations of population burden deriving from active headache. In addition, recall error is probably a bigger problem when asking people about their whole lifetimes rather than recent time periods.

The opposites apply to 1-day prevalence. Enquiry into headache yesterday may almost eliminate recall error. Although 1-day prevalence is substantially lower than 1-year prevalence, at least for episodic headache, it offers a very sound basis for measuring population burden, provided that samples are large enough. However, questions about headache yesterday provide reliable data only in “on-the-spot” interviews (face-to-face or by telephone): in internet surveys or those relying on mailed questionnaires, participants with headache on the day they receive it may postpone answering it until the first day without, then truthfully but spuriously report headache yesterday. The relatively few studies reporting 1-day prevalence suggest that 15.8% of the world’s population have headache on any day, and almost half of them migraine (7.0%).

### Study quality

We found no significant association between study quality score [4] and prevalence. This does not mean that the quality criteria are meaningless: higher quality lends credibility to findings. While the criteria may need refinement, they are based on sound general epidemiological principles, to which all future studies should endeavour to adhere. However, higher scores on one criterion may tend to increase prevalence estimates (e.g., distinction between definite and probable migraine), and higher scores on another have the opposite effect (e.g., random sampling), with the net result being very small. This probably explains why quality score explains so little of the variability in our models (Table 7).

### Burden estimates

While prevalence is a determining component of disease-attributed burden, it provides no estimate of it in the population. Headache-attributed burden not only has multiple and diverse components [15] but also is very unevenly distributed in populations with current headache, being quite low in the relatively large proportions who have infrequent attacks of mild or moderate intensity [28]. Of the two further factors that must be known to estimate burden – the level of burden associated with the ictal (symptomatic) state and the proportion of time spent in this state – both can be assessed as population averages. The former may be a constant at population level for each headache type (the view taken in GBD studies, with “disability weights” assessing lost health), so that a sound estimate of population burden for all headache disorders may be based on the mean Time in Ictal State (TIS) (or Time in Symptomatic State in the GBD studies) [29]. Estimates of disease-attributed burden based on mean TIS may be relatively insensitive to estimated prevalence if higher estimates of the latter are generated by more searching enquiry, identifying participants with very low TIS. But all studies are likely to capture those with high burden, so that burden estimates are highly sensitive to participation bias.

It is worth noting that most cases of pMig fail the attack duration criterion (< 4 h for adults, < 2 h for children) [30–32]. Since duration is a factor in TIS, inclusion or not of probable diagnoses has rather less impact on burden estimates than on prevalence estimates.

### Study limitations

Including not only strictly population-based prevalence studies but also studies on all other non-clinical samples made a rules-based literature search strategy difficult. This might have introduced some subjectivity and unknown study-selection biases.

The fact that dependent variables were not normally distributed might have rendered the MLR analysis less reliable. However, we believe that, by also presenting a model with normalized variables, we have generated results that for explorative purposes are robust, although the exact size of the contribution of each variable remains uncertain.

The fact that < 30% of the variability could be explained by the variables included in this analysis (time and place of studies, age and gender of populations, and methodological variables) indicates that there are other influential variables that are not yet understood or measured. We can only speculate as to what these may be: population differences in genes, general health, quality of health services for headache, exposure to climatic factors, light or altitude, nutritional factors, chemicals and air pollution, level of stress, attitudes towards pain and knowledge about headache in the population. Even differences in connotations in the words used for headache or migraine may play a role.

## Conclusions

While this review updates our earlier documentation of headache epidemiological studies [5], it also highlights the dependence of prevalence estimates on a small number of methodological factors (and relative independence of others that might be expected to be influential). All future studies should use validated questionnaires, describe the screening question *verbatim* and explain how they deal with definite and probable diagnoses. Ideally, future prevalence estimates from all parts of the world will be derived from studies performed in a relatively standardized way, in accordance with published recommendations.

Also, future studies should not assess prevalence alone but include data allowing TIS to be estimated, preferably among the various age and gender subgroups.

## Supplementary Information

**Additional file 1.** Studies included in the analysis.

**Additional file 2.**

## Data Availability

All data are available in the published studies referred to in the supplementary material (Additional file 1).
