# Supplementary material for: The global prevalence of headache: an update, with analysis of the influences of methodological factors on prevalence estimates
Source: J Headache Pain. 2022 Apr 12;23(1):34. doi: 10.1186/s10194-022-01402-2 (PMC9004186; doi:10.1186/s10194-022-01402-2)
Supplement: Supplementary file 1 — Additional file 1. Studies included in the analysis. [file 10194_2022_1402_MOESM1_ESM.docx]

| Appendix 1 Studies included in the analysis | | | | | | | | |
| --- | --- | --- | --- | --- | --- | --- | --- | --- |
|  |  |  |  |  | Prevalences (% of population) for both sexes* | | | |
| Country | Year | Time-frame | N | Age | Headache | Migraine | TTH | H15+ |
| Benin [1] | 2009 | N.s. | 336 | 31.0 |  | 12.60 |  |  |
| Benin [2] | 2010 | L.t. | 1113 | 47.5 |  | 3.30 |  |  |
| Egypt [3] | 2010 | N.s. | 33285 | 46.5 |  | 2.80 |  |  |
| Egypt [4] | 2015 | 1-y | 2375 | 50.0 | 51.40 | 17.30 | 24.50 | 6.6 |
| Egypt [5] | 2016 | 1-y | 4700 | 45.0 |  | 10.50 | 13.60 |  |
| Ethiopia [6] | 1995 | 1-y | 15000 | 52.5 |  | 3.00 |  | 1.7 |
| Ethiopia [7] | 2008 | 1-y | 1105 | 51.0 | 74.40 | 6.90 | 11.00 |  |
| Ethiopia [8] | 2013 | N.s. | 2151 | 42.5 |  | 9.80 |  |  |
| Ethiopia [9] | 2013 | N.s. | 231 | 50.5 | 67.50 | 10.90 | 10.40 |  |
| Ethiopia [10] | 2016 | 1-y | 2385 | 41.5 | 44.90 | 19.00 | 20.70 | 4.6 |
| Ethiopia [10] | 2016 | 24 h | 2385 | 41.5 |  | 7.10 |  |  |
| Ethiopia [11] | 2020 | 1-y | 2344 | 12.0 | 73.60 | 38.40 | 20.30 | 1.3 |
| Ethiopia [11] | 2020 | Point | 2344 | 12.0 |  | 15.60 | 6.70 | 0.8 |
| Mali [12] | 2017 | N.s. | 733 | 18.5 |  | 18.90 |  |  |
| Nigeria [13] | 1984 | N.s. | 1756 | 24.0 | 60.00 | 17.70 |  |  |
| Nigeria [14] | 1994 | N.s. | 1540 | 40.0 | 25.70 | 8.80 |  |  |
| Nigeria [15] | 2009 | 1-y | 376 | 24.0 | 50.00 | 15.60 | 28.60 |  |
| Nigeria [16] | 2009 | L.t. | 1513 | 22.0 |  | 9.60 |  |  |
| Nigeria [17] | 2010 | 1-y | 1679 | 14.5 | 19.50 | 13.50 |  |  |
| Nigeria [18] | 2012 | 1-y | 171 | 28.5 | 88.60 | 18.40 |  |  |
| Nigeria [19] | 2012 | N.s. | 239 | 46.5 | 39.30 |  |  |  |
| Nigeria [20] | 2014 | N.s. | 1410 | 44.5 | 66.70 | 6.40 | 13.80 |  |
| Nigeria [21] | 2018 | 1-y | 1500 | 28.0 | 23.80 | 2.40 | 12.40 |  |
| Nigeria [22] | 1989 | N.s. | 2925 | 52.0 |  | 6.30 |  |  |
| Nigeria [23] | 1992 | L.t. | 18954 | 37.5 | 51.00 | 5.30 |  |  |
| Nigeria [24] | 1997 | N.s. | 4398 | 9.5 |  | 6.80 |  |  |
| Tanzania [25] | 2004 | 1-y | 3351 | 48.0 | 23.10 | 5.00 |  |  |
| Tanzania [26,27] | 2009 | 1-y | 7412 | 42.5 | 12.10 | 3.90 | 7.00 |  |
| Tunisia [28] | 1993 | N.s. | 34874 | 52.5 |  | 3.40 |  |  |
| Uganda [29] | 2016 | N.s. | 3000 | 36.5 | 14.50 |  |  |  |
| Zambia [30] | 2015 | 1-y | 1085 | 41.5 | 72.00 | 23.30 | 24.20 | 19.1 |
| Zambia [30] | 2015 | 24 h | 1085 | 41.5 | 28.30 |  |  |  |
| Zimbabwe [31] | 2006 | 1-y | 175 | 34.0 | 37.10 | 13.10 | 17.70 |  |
| Zimbabwe [32] | 1983 | 1-y | 5028 | 37.5 | 20.00 |  |  |  |
| Zimbabwe [32] | 1983 | N.s. | 5028 | 37.5 | 20.00 |  |  |  |
| China [33] | 1988 | N.s. | 246812 | 45.0 |  | .70 |  |  |
| China [34] | 2012 | 1-y | 5041 | 41.5 | 28.50 | 9.30 | 10.80 | 1.0 |
| China [35] | 2013 | 1-y | 5519 | 50.0 |  | 10.00 |  |  |
| China [36] (Hong Kong) | 1995 | N.s. | 7356 | 50.0 | 5.00 | 1.60 | 3.40 |  |
| China [37] (Hong Kong) | 2000 | N.s. | 1436 | 50.0 | 37.10 | 4.70 | 26.90 |  |
| China [38] | 2001 | 1-y | 2120 | 10.5 | 2.80 | .50 | 1.20 |  |
| China [39] | 2013 | 3-m | 4812 | 10.5 | 9.70 | 4.40 | 2.80 |  |
| China [40] | 2013 | 24 h | 5041 | 41.5 | 4.80 |  |  |  |
| China [41] | 2016 | 3-m | 1143 | 51.5 |  | 8.20 |  |  |
| China [42] | 2016 | 1-y | 5038 | 72.5 |  |  |  |  |
| China [43] | 1997 | 1-y | 1533 | 75.0 | 38.00 | 3.10 |  | 3.9 |
| India [44] | 2012 | N.s. | 5000 | 13.0 | 65.10 | 26.10 | 33.90 | 5.4 |
| India [45] | 2013 | N.s. | 422 | 24.5 | 68.00 | 13.90 |  |  |
| India [46] | 2003 | N.s. | 1305 | 13.0 | 19.50 | 11.00 |  |  |
| India [47] | 2004 | N.s. | 102557 | 45.0 | 1.10 |  |  |  |
| India [48] | 2009 | 1-y | 2235 | 15.0 | 57.50 | 29.80 | 11.00 |  |
| India [49] | 2015 | 1-y | 2329 | 41.5 |  | 25.60 | 34.80 | 4.2 |
| India [50] | 2016 | 24 h | 2329 | 41.5 | 5.90 |  |  |  |
| India [51] | 2017 | 1-y | 2421 | 35.0 |  | 14.10 |  |  |
| Iran [52] | 1998 | N.s. | 1029 | 35.5 | 64.00 | 10.40 |  |  |
| Iran [53] | 2013 | N.s. | 480 | 24.5 |  | 14.20 | 44.60 |  |
| Iran [54] | 2002 | N.s. | 1868 | 14.5 |  |  |  |  |
| Iran [55] | 2006 | 1-y | 2226 | 8.0 |  | 1.70 | 5.50 |  |
| Iran [56] | 2011 | 1-y | 930 | 13.5 |  | 12.30 | 4.20 |  |
| Iran [57] | 2013 | 1-y | 3655 | 48.0 |  | 16.30 | 48.40 | 6.9 |
| Iran [58] | 2016 | 1-y | 2076 | 38.5 | 27.60 |  |  |  |
| Israel [59] | 1980 | N.s. | 4899 | 50.0 | 75.80 | 10.10 |  |  |
| Japan [60] | 2007 | N.s. | 6472 | 13.5 | 59.80 | 4.90 |  |  |
| Japan [61] | 1997 | L.t. | 4029 | 50.0 | 68.00 |  |  |  |
| Japan [62] | 2004 | 1-y | 4795 | 50.0 | 28.50 | 6.00 | 21.70 | 2.1 |
| Japan [63] | 2017 | L.t. | 3285 | 10.5 | 49.40 | 4.90 | 16.40 |  |
| Japan [61] | 1997 | 1-y | 4029 | 50.0 | 55.60 | 8.40 | 22.40 |  |
| Jordan [64] | 2017 | N.s. | 866 | 17.0 | 67.20 | 8.80 | 19.00 |  |
| Jordan [65] | 2009 | N.s. | 4836 | 51.5 | 82.30 | 7.70 | 36.90 |  |
| Kuwait [66] | 2014 | 3-m | 621 | 21.5 |  | 27.90 |  |  |
| Malaysia [67] | 1996 | 1-y | 561 | 45.0 |  | 9.00 | 26.50 |  |
| Malaysia [67] | 1996 | L.t. | 561 | 45.0 | 79.90 |  |  |  |
| Mongolia [68] | 2020 | 1-y | 4266 | 12.0 | 61.30 | 28.00 | 16.80 | 4.4 |
| Mongolia [68] | 2020 | Point | 4266 | 12.0 | 15.90 | 7.50 | 16.90 | 3.5 |
| Nepal [69] | 2015 | 1-y | 2100 | 41.5 | 85.40 | 34.70 | 41.10 | 9.9 |
| Oman [70] | 2001 | 1-y | 403 | 22.0 | 96.80 | 12.20 | 12.20 |  |
| Oman [71] | 2002 | 1-y | 1158 | 47.5 | 78.80 | 10.10 | 11.20 |  |
| Oman [71] | 2002 | L.t. | 1158 | 47.5 |  |  |  |  |
| Pakistan [72] | 2006 | L.t. | 1211 | 16.0 | 85.50 |  |  |  |
| Pakistan [73] | 2017 | 1-y | 4223 | 42.0 | 76.1 | 22.5 | 44.6 | 8.2 |
| Qatar [74] | 2006 | 1-y | 913 | 50.5 | 72.50 | 7.90 |  |  |
| Qatar [75] | 2005 | 1-y | 851 | 11.5 | 85.00 |  |  |  |
| South Korea [76] | 2019 | N.s. | 2695 | 44.0 |  | 19.50 |  |  |
| Saudi Arabia [77] | 2009 | N.s. | 1750 | 18.5 | 33.10 | 7.70 |  |  |
| Saudi Arabia [78] | 2018 | N.s. | 4943 | 30.5 | 83.10 | 25.40 |  |  |
| Saudi Arabia [79] | 2019 | 3-m | 1340 | 24.0 |  |  |  |  |
| Saudi Arabia [80] | 2020 | 1-y | 2316 | 41.5 | 65.80 | 28.70 | 42.90 | 4.3 |
| Saudi Arabia [81] | 2002 | 1-y | 1181 | 11.0 |  | 6.20 |  |  |
| Saudi Arabia [82] | 1993 | Point | 23227 | 45.0 | 13.10 |  |  |  |
| Saudi Arabia [83] | 1996 | L.t. | 5891 | 50.0 | 8.00 |  |  |  |
| Singapore [84] | 1997 | N.s. | 1208 | 22.0 | 98.10 | 12.00 | 29.80 |  |
| Singapore [85] | 2016 | L.t. | 6126 | 51.5 |  | 8.20 |  |  |
| Singapore [86] | 2003 | Point | 2096 | 48.5 | 5.20 |  |  |  |
| Singapore [86] | 2003 | L.t. | 2096 | 48.5 | 82.70 | 9.30 | 42.30 | 1.4 |
| Singapore [87] | 2010 | L.t. | 2873 | 11.0 | 80.60 | 21.20 | 29.60 | 7.1 |
| South Korea [88] | 2013 | 1-y | 1507 | 44.0 | 61.40 | 17.60 |  |  |
| South Korea [89] | 2013 | 1-y | 1507 | 44.0 |  |  | 30.80 |  |
| South Korea [90] | 1998 | 1-y | 5556 | 50.0 | 68.00 | 22.30 |  |  |
| Taiwan [91] | 2005 | 1-y | 13426 | 14.0 |  | 6.80 |  |  |
| Taiwan [92] | 2006 | 1-y | 7900 | 13.0 |  |  |  | 1.5 |
| Taiwan [93] | 2006 | L.t. | 107 | 45.0 |  |  |  |  |
| Taiwan [94] | 2010 | L.t. | 3963 | 14.0 | 86.60 | 23.40 | 27.60 |  |
| Taiwan [95] | 2000 | 1-y | 3377 | 50.0 | 62.00 | 8.70 |  |  |
| Taiwan [96] | 2000 | L.t. | 4064 | 14.0 | 84.60 | 6.80 |  |  |
| Taiwan [97] | 2001 | 1-y | 3377 | 50.0 |  |  |  | 3.2 |
| Thailand [98] | 2007 | N.s. | 1789 | 13.5 |  | 13.80 |  |  |
| Thailand [99] | 2010 | 3-m | 953 | 13.0 | 87.40 | 12.20 | .70 |  |
| Thailand [100] | 1989 | 1-y | 540 | 47.5 |  | 22.90 |  |  |
| Thailand [101] | 1991 | 1-y | 241 | 73.0 | 54.80 |  |  |  |
| UAE [102] | 1998 | 1-y | 1159 | 10.0 | 36.90 | 3.80 |  |  |
| Yemen [103] | 2015 | 1-y | 12640 | 51.5 | 76.60 | 16.30 | 31.50 | 8.6 |
| Australia [104] | 1990 | L.t | 900 | 14.0 | 63.20 |  |  |  |
| Australia [105] | 1994 | 1-y | 851 | 8.5 | 22.00 |  |  |  |
| Australia [106] | 1998 | L.t. | 3654 | 67.0 |  | 17.00 |  |  |
| New Zealand [107] | 2014 | 1-y | 617 | 11.0 | 42.80 | 10.50 | 18.60 |  |
| New Zealand [108] | 2002 | 1-y | 980 | 26.0 | 24.50 | 11.60 | 11.10 |  |
| New Zealand [109] | 1985 | 1-y | 1139 | 50.5 | 50.00 |  |  |  |
| New Zealand [110] | 1993 | N.s. | 2725 | 43.5 |  |  |  |  |
| 9 European countries [111] | 2014 | 1-y | 8271 | 41.5 | 79.60 | 36.50 | 37.60 | 7.6 |
| Albania [112] | 2012 | Current | 9869 | 50.5 | 21.20 |  |  |  |
| Austria [113] | 2019 | 1-y | 3470 | 14.0 | 75.70 | 24.30 | 21.60 | 3.0 |
| Austria [114] | 2003 | 1-y | 997 | 50.0 | 49.40 | 10.20 |  |  |
| Belgium [115] | 2007 | N.s. | 1660 | 42.5 | 50.70 | 20.20 |  |  |
| Belgium [116] | 2008 | 1-y | 1467 | 43.0 | 14.50 | 10.70 |  |  |
| Bulgaria [117] | 2012 | N.s. | 1029 | 12.0 | 40.00 | 12.80 | 17.00 |  |
| Croatia [118] | 2009 | L.t. | 314 | 22.5 |  | 9.90 | 58.90 |  |
| Croatia [119] | 2001 | 1-y | 3794 | 40.0 |  | 16.70 |  |  |
| Croatia [119,120] | 2003 | L.t. | 3794 | 40.0 | 65.20 | 19.00 | 34.80 |  |
| Croatia [121,122] | 2010 | 1-y | 1542 | 51.5 | 39.90 | 15.00 | 41.40 |  |
| Croatia [121,122] | 2013 | 1-y | 1542 | 51.5 |  |  |  | 2.4 |
| Croatia [123] | 2014 | 1-y | 2057 | 16.0 | 30.10 | 14.20 | 15.90 |  |
| Croatia [124] | 2016 | N.s. | 1876 | 16.5 |  | 15.70 | 41.20 |  |
| Denmark [125] | 1995 | L.t. | 3471 | 40.0 |  | 18.70 |  |  |
| Denmark [126] | 2002 | L.t. | 4660 | 43.5 |  | 20.50 |  |  |
| Denmark [127] | 2012 | 1-y | 31865 | 45.5 |  | 12.30 |  |  |
| Denmark [127] | 2012 | L.t. | 31865 | 45.5 |  | 25.20 |  |  |
| Denmark [128] | 2015 | 1-y | 797 | 50.5 |  | 23.00 | 30.20 |  |
| Denmark [129,130] | 2020 | 3-m | 55185 | 50.5 |  |  |  | 2.7 |
| Denmark [130] | 2005 | 1-y | 207 | 30.5 |  | 15.50 | 86.50 |  |
| Denmark [130] | 2005 | L.t. | 207 | 30.5 |  | 18.40 | 89.40 |  |
| Denmark [130] | 2005 | Point | 207 | 30.5 | 16.40 |  |  |  |
| Denmark [131] | 2014 | 3-m | 68518 | 50.5 |  |  |  | 3.3 |
| Denmark [132] | 1991 | 1-y | 740 | 44.5 |  | 10.00 | 74.00 | 3.0 |
| Denmark [132] | 1991 | L.t. | 740 | 44.5 | 96.00 | 17.90 | 86.90 |  |
| Estonia [133] | 2019 | 1-y | 1215 | 41.0 | 41.00 | 17.70 | 18.10 | 2.7 |
| Finland [134] | 1976 | current | 4235 | 7.0 | 37.70 | 3.20 |  |  |
| Finland [134] | 1984 | 1-y | 3863 | 14.0 | 68.00 | 10.30 |  |  |
| Finland [135] | 1983 | N.s. | 2921 | 14.0 | 69.00 | 10.60 |  |  |
| Finland [136] | 1993 | N.s. | 24682 | 44.5 |  | 6.25 |  |  |
| Finland [137] | 1996 | 6-m | 1436 | 7.0 | 71.10 | 5.70 |  |  |
| Finland [138] | 2009 | N.s. | 4236 | 14.0 | 63.00 |  |  |  |
| Finland [139] | 1981 | 1-y | 200 | 50.5 | 77.00 |  |  |  |
| Finland [139] | 1981 | L.t. | 200 | 50.5 | 91.00 |  |  |  |
| Finland [140] | 1983 | 1-y | 3784 | 13.0 | 82.00 |  |  |  |
| Finland [141] | 1991 | 1-y | 4405 | 5.0 | 19.50 |  |  |  |
| Finland [142] | 1994 | 6-m | 3580 | 8.5 | 36.50 | 2.70 |  |  |
| France [143] | 1993 | N.s. | 1563 | 46.0 |  | 3.90 |  |  |
| France [144] | 2003 | 1-y | 1000 | 44.0 |  | 5.00 |  |  |
| France [145] | 2010 | N.s. | 368 | 45.0 |  | 51.30 |  |  |
| France [146] | 2011 | L.t. | 780 | 71.0 |  | 14.90 |  |  |
| France [147] | 2002 | N.s. | 10585 | 50.0 | 29.20 | 17.30 |  | 3.0 |
| France [148] | 1996 | 3-m | 9411 | 52.0 | 49.00 | 13.00 |  |  |
| France [149] | 2003 | L.t. | 10585 | 50.0 |  |  |  | 3.0 |
| France [150] | 2005 | N.s. | 10532 | 51.5 |  | 21.30 |  |  |
| France [151] | 1992 | Current | 4204 | 35.0 | 35.00 | 12.10 |  |  |
| Georgia [152,153] | 2007 | 1-y | 93 | 41.5 | 63.10 | 6.70 | 19.40 | 3.6 |
| Georgia [153] | 2009 | 1-y | 1145 | 50.5 | 74.50 | 15.70 | 37.30 | 7.6 |
| Germany [154] | 1992 | Current | 825 | 40.5 |  | 22.10 |  |  |
| Germany [155] | 1994 | N.s. | 6895 | 12.0 | 89.20 | 11.00 | 48.70 |  |
| Germany [144] | 2003 | 1-y | 1000 | 44.0 |  | 11.00 |  |  |
| Germany [156] | 2007 | N.s. | 3336 | 50.0 |  | 13.90 |  |  |
| Germany [157] | 2008 | 6-m | 3945 | 11.5 |  | 5.60 | 27.10 |  |
| Germany [158] | 2009 | 6-m | 7417 | 55.0 |  | 11.20 | 31.50 |  |
| Germany [159] | 2009 | L.t. | 7124 | 48.5 |  | 11.70 |  |  |
| Germany [160] | 2010 | 6-m | 1136 | 15.0 | 47.80 | 4.10 | 21.10 |  |
| Germany [161] | 2014 | 6-m | 1445 | 15.5 |  | 9.30 | 83.00 |  |
| Germany [162] | 2015 | 6-m | 1674 | 15.5 | 83.60 | 15.70 | 6.90 | 7.2 |
| Germany [163] | 2019 | 3-m | 2706 | 13.0 | 68.10 |  |  | 2.2 |
| Germany [164] | 2004 | 3-m | 735 | 14.0 | 66.00 |  |  |  |
| Germany [165] | 2007 | 3-m | 3324 | 13.5 | 69.40 | 15.30 | 20.20 |  |
| Germany [166] | 2007 | 6-m | 5586 | 10.5 | 53.50 | 7.50 | 18.50 |  |
| Germany [167] | 2009 | 1-y | 7341 | 51.5 | 60.20 | 10.60 |  |  |
| Germany [168] | 2009 | 6-m | 3833 | 11.5 | 66.30 | 19.70 | 17.60 |  |
| Germany [169] | 2010 | 3-m | 6536 | 55.0 |  |  |  |  |
| Germany [170] | 2011 | 6-m | 1260 | 15.0 | 83.10 | 10.30 | 76.60 |  |
| Germany [171] | 2011 | 3-m | 14836 | 10.0 | 44.00 |  |  |  |
| Germany [172] | 2012 | 1-y | 9944 | 41.5 | 59.30 | 17.80 | 13.30 | 2.6 |
| Germany [173] | 2013 | 1-y | 12120 | 49.5 | 62.30 |  |  |  |
| Germany [174] | 1994 | L.t. | 4061 | 51.5 | 71.40 | 27.50 | 13.30 |  |
| Greece [175] | 1994 | 6-m | 449 | 39.0 |  |  |  |  |
| Greece [176] | 1996 | 1-y | 3501 | 45.0 | 29.00 |  |  | 4.5 |
| Greece [177] | 1999 | 1-y | 3509 | 9.5 |  | 6.20 |  |  |
| Hungary [178] | 2000 | 1-y | 813 | 47.5 |  | 9.50 |  |  |
| Hungary [179] | 2013 | 1-y | 7361 | 12.5 |  | 12.50 |  |  |
| Italy [144] | 2003 | 1-y | 1000 | 44.0 |  | 12.00 |  |  |
| Italy [180] | 2008 | N.s. | 1073 | 11.5 | 19.20 | 6.50 |  |  |
| Italy [181] | 2009 | N.s. | 93 | 16.5 |  |  |  |  |
| Italy [182] | 2015 | 1-y | 904 | 51.5 |  |  | 22.90 |  |
| Italy [183] | 2017 | 1-y | 1270 | 51.5 | 47.00 | 21.70 | 19.40 |  |
| Italy [183] | 2017 | L.t. | 1270 | 51.5 | 69.10 |  |  |  |
| Italy [184] | 2018 | L.t. | 1950 | 13.5 | 65.90 |  |  |  |
| Italy [185] | 2001 | 1-y | 833 | 75.0 | 51.00 | 11.00 |  | 4.4 |
| Italy [186] | 2003 | 1-y | 1031 | 75.0 | 21.80 | 4.60 |  |  |
| Italy [187] | 1995 | 1-y | 1445 | 12.5 | 23.90 | 4.50 |  |  |
| Italy [188] | 2009 | L.t. | 574 | 74.5 | 51.70 | 19.30 | 41.90 |  |
| Italy [188] | 2009 | 1-y | 574 | 74.5 |  | 5.70 | 35.80 |  |
| Italy [189] | 2009 | 6-m | 4386 | 13.0 | 40.50 |  |  |  |
| Italy [190] | 2011 | N.s. | 1536 | 17.0 | 62.00 |  |  |  |
| Italy [191] | 2012 | 1-y | 904 | 51.5 |  | 25.90 |  |  |
| Italy [192] | 2014 | 6-m | 649 | 7.0 | 26.30 |  |  |  |
| Italy [193] | 2015 | 1-y | 487 | 41.5 | 74.20 | 40.30 | 28.60 | 7.0 |
| Italy [193] | 2015 | L.t. | 487 | 41.5 | 82.50 |  |  |  |
| Lithuania [194] | 2017 | 1-y | 573 | 41.5 | 76.30 | 20.40 | 41.90 | 8.7 |
| Lithuania [194] | 2017 | 24 h | 573 | 41.5 | 12.20 |  |  |  |
| Lithuania [195] | 2020 | 1-y | 2505 | 12.0 | 73.80 | 20.60 | 24.10 | 2.9 |
| Netherlands [196] | 1999 | 1-y | 6491 | 42.5 |  | 16.30 |  |  |
| Netherlands [196] | 1999 | L.t. | 6491 | 42.5 |  | 23.20 |  |  |
| Norway [197] | 2008 | 1-y | 297 | 52.5 | 74.10 | 19.60 | 54.60 | 5.7 |
| Norway [198] | 2011 | 1-y | 39690 | 51.5 | 37.40 | 17.90 | 16.50 | 2.5 |
| Norway [199] | 2012 | 1-y | 245 | 52.5 |  | 26.30 |  |  |
| Norway [199] | 2012 | Lt | 245 | 52.5 |  | 28.30 |  |  |
| Norway [200] | 2015 | 6-m | 19985 | 16.0 |  |  |  | 4.8 |
| Norway [201] | 2018 | 1-y | 232 | 55.5 | 68.30 | 19.20 | 42.50 | 3.2 |
| Norway [201] | 2018 | L.t. | 232 | 55.5 |  | 30.10 |  |  |
| Norway [202] | 2019 | 1-y | 20486 | 69.5 | 30.70 | 8.50 |  |  |
| Norway [203] | 2020 | 1-y | 41460 | 52.5 | 37.20 | 15.90 | 20.70 | 2.1 |
| Norway [204] | 2004 | 1-y | 8255 | 16.0 | 76.80 | 7.00 | 18.00 | .5 |
| Norway [205] | 2000 | 1-y | 51383 | 52.5 | 37.70 | 11.60 |  | 2.4 |
| Norway [206] | 2008 | L.t. | 21780 | 50.0 | 77.20 | 26.10 |  |  |
| Norway [206] | 2008 | 1-y | 21780 | 50.0 | 26.10 |  |  |  |
| Norway [207] | 2008 | 1-y | 20598 | 37.0 |  |  |  | 2.8 |
| Norway [208] | 2010 | L.t. | 10176 | 52.0 |  |  |  |  |
| Norway [209] | 2015 | 1-y | 488 | 15.0 | 87.70 | 36.00 | 58.00 |  |
| Norway [209] | 2015 | Point | 488 | 15.0 | 38.00 |  |  |  |
| Poland [210] | 1999 | 1-y | 2353 | 12.5 | 75.00 | 8.40 | 28.70 |  |
| Poland [211] | 1999 | 1-y | 2352 | 17.0 |  | 18.20 |  |  |
| Poland [212] | 1999 | 1-y | 2352 | 17.0 |  |  | 57.60 |  |
| Portugal [213] | 1994 | N.s. | 491 | 23.0 |  | 6.10 | 16.00 |  |
| Portugal [214] | 1995 | L.t. | 2008 | 51.0 | 89.9 | 8.5 | 65.9 |  |
| Russia [215] | 2012 | 1-y | 2025 | 41.5 | 63.10 | 20.30 | 30.90 | 10.5 |
| Russia [216] | 2012 | 24 h | 2025 | 41.5 | 14.50 | 3.60 | 3.50 |  |
| Russia [217] | 2016 | 1-y | 3124 | 42.0 |  | 15.90 | 58.80 |  |
| San Marino [218] | 1988 | 1-y | 1144 | 46.0 | 46.00 |  |  |  |
| Serbia [219] | 2007 | N.s. | 1259 | 9.5 | 32.80 | 3.30 | 1.30 |  |
| Slovenia [220] | 2013 | 1-m | 1002 | 50.5 | 27.30 |  |  |  |
| Spain [221] | 1994 | Current | 316 | 40.5 |  | 29.70 |  |  |
| Spain [222] | 1995 | Current | 96 | 24.0 |  | 20.80 |  |  |
| Spain [223] | 1999 | L.t. | 548 | 51.5 |  | 12.60 |  |  |
| Spain [223] | 1999 | 1-y | 548 | 51.5 | 80.00 | 11.30 |  |  |
| Spain [224] | 2003 | 1-y | 21650 | 50.5 |  | 6.50 |  |  |
| Spain [224] | 2006 | 1-y | 29436 | 50.5 |  | 14.70 |  |  |
| Spain [224] | 2009 | 1-y | 22188 | 50.5 |  | 11.10 |  |  |
| Spain [224] | 2011 | 1-y | 20884 | 50.5 |  | 9.70 |  |  |
| Spain [225] | 2013 | 1-y | 993 | 50.5 |  | 29.40 |  |  |
| Spain [225] | 2013 | 1-y | 16079 | 50.5 |  | 12.50 |  |  |
| Spain [226] | 2019 | N.s. | 1619 | 15.0 | 30.50 | 11.40 |  |  |
| Spain [227] | 2002 | 6-m | 1964 | 51.5 | 78.60 |  |  |  |
| Spain [228] | 2011 | 1-y | 5668 | 41.5 |  | 12.60 |  |  |
| Spain [221] | 1994 | L.t. | 2231 | 40.5 | 86.70 | 12.00 |  |  |
| Spain [229] | 1997 | N.s. | 669 | 10.0 |  | 7.00 |  |  |
| Spain [230] | 1999 | 1-y | 2253 | 49.5 |  |  |  | 4.7 |
| Sweden [231] | 2014 | N.s. | 464 | 15.0 | 71.90 | 24.90 | 37.60 |  |
| Sweden [232] | 2001 | 1-y | 1668 | 46.0 | 63.00 | 17.00 |  |  |
| Sweden [233] | 2000 | 1-y | 728 | 57.0 |  |  |  |  |
| Sweden [233] | 2000 | L.t. | 722 | 57.0 |  |  |  |  |
| Sweden [234] | 2004 | 1-y | 1371 | 11.0 | 44.80 | 17.60 | 19.30 |  |
| Sweden [235] | 2007 | 2-3 m | 1908 | 12.0 | 13.00 |  |  |  |
| Sweden [236] | 2006 | 3-m | 43770 | 48.5 | 16.80 | 4.00 |  |  |
| Sweden [237] | 2009 | N.s. | 2991 | 49.5 |  |  |  |  |
| Sweden [238] | 2011 | Point | 44300 | 50.0 |  |  |  | 3.2 |
| Sweden [239] | 2012 | 6-m | 1033 | 17.0 |  |  |  |  |
| Sweden [240] | 1962 | L.t. | 8993 | 11.0 | 58.70 | 3,9 |  |  |
| Switzerland [241] | 2011 | 1-y | 591 | 49.5 | 33.50 | 9.20 | 11.70 |  |
| Switzerland [242] | 2013 | 3-m | 1192 | 42.5 | 57.60 | 18.50 | 47.50 |  |
| Switzerland [243] | 1994 | 1-y | 379 | 29.5 | 57.00 | 24.60 | 15.80 |  |
| Turkey [244] | 2013 | N.s. | 1618 | 22.5 |  | 10.60 |  |  |
| Turkey [245] | 2015 | 6-m | 10584 | 12.0 | 47.80 | 7.10 | 7.70 |  |
| Turkey [246] | 2002 | L.t. | 947 | 46.0 |  | 12.50 |  |  |
| Turkey [247] | 2003 | 1-y | 1146 | 54.5 |  |  |  |  |
| Turkey [248] | 2004 | N.s. | 2490 | 14.5 |  | 8.80 |  |  |
| Turkey [249] | 2005 | L.t | 1835 | 30.0 |  |  |  |  |
| Turkey [250] | 2005 | L.t. | 386 | 50.0 |  | 19.40 |  |  |
| Turkey [251] | 2006 | 1-y | 2387 | 14.5 | 52.20 | 14.50 | 25.90 |  |
| Turkey [252] | 2007 | L.t. | 2384 | 16.0 | 45.70 | 20.50 | 5.10 | 0.4 |
| Turkey [252] | 2007 | 1-y | 2384 | 16.0 |  | 9.60 | 2.30 |  |
| Turkey [253] | 2007 | L.t. | 7721 | 13.0 | 83.40 | 9.70 |  |  |
| Turkey [254] | 2009 | N.s. | 2669 | 8.5 | 46.20 | 3.40 |  |  |
| Turkey [255] | 2010 | 1-y | 1385 | 14.5 | 38.90 | 25.50 | 16.30 |  |
| Turkey [256] | 2012 | 1-y | 5323 | 41.5 | 44.60 | 16.40 | 5.10 | 3.3 |
| Turkey [257] | 2018 | 1-y | 7068 | 11.5 | 73.70 | 7.30 | 12.90 | 3.4 |
| Turkey [258] | 2005 | N.s. | 5777 | 12.0 | 49.20 | 10.40 | 24.70 | 1.5 |
| Two european countries [259](Austria and Turkey) | 2014 | 1-y | 1202 | 11.5 | 89.30 | 39.30 | 37.30 | 4.5 |
| Two european countries [259](Austria and Turkey) | 2014 | 24h | 491 | 11.5 | 29.6 |  |  |  |
| UK [260] | 1961 | L.t. | 1607 | 42.5 |  | 9.20 |  |  |
| UK [261] | 1973 | 1-y | 1718 | 53.5 | 71.00 |  |  |  |
| UK [262] | 1977 | N.s. | 14893 | 36.0 |  | 13.10 |  |  |
| UK [263] | 1992 | Current | 1083 | 7.0 | 55.50 | 4.90 |  |  |
| UK [264] | 1995 | 1-y | 476 | 42.5 |  | 17.40 |  |  |
| UK [144] | 2003 | 1-y | 1000 | 44.0 |  | 7.00 |  |  |
| UK [265] | 2003 | 1-y | 4007 | 40.5 |  | 14.30 |  |  |
| UK [266] | 2003 | L.t. | 1662 | 51.5 | 92.60 |  |  |  |
| UK [266] | 2003 | 3-m | 1662 | 51.5 | 70.30 |  |  |  |
| UK [267] | 2005 | 1-y | 1589 | 54.0 | 76.00 |  |  |  |
| UK [268] | 1977 | L.t. | 727 | 46.5 | 81.30 |  |  |  |
| UK [269] | 1977 | 1-y | 600 | 15.0 | 78.00 |  |  |  |
| UK [270] | 1994 | 1-y | 2165 | 10.0 | 66.00 | 11.20 | .90 |  |
| Canada [271] | 1994 | 1-y | 2922 | 52.0 |  |  |  |  |
| Canada [271] | 1994 | L.t. | 2922 | 52.0 |  | 17.10 |  |  |
| Canada [272] | 2001 | N.s. | 14619 | 48.5 |  | 7.90 |  |  |
| Canada [273] | 2008 | current | 36984 | 50.0 |  | 10.70 |  |  |
| Canada [274] | 2012 | Current | 15254 | 48.5 |  | 7.20 |  |  |
| Canada [275] | 2014 | Current | 22720 | 42.5 |  | 7.80 |  |  |
| Canada [276] | 2019 | L.t. | 43725 | 65.0 |  | 13.50 |  |  |
| Canada [277] | 2010 | N.s. | 1210 | 51.5 |  |  |  |  |
| Canada [278] | 1992 | N.s. | 2737 | 50.0 | 57.00 | 16.20 | 20.40 |  |
| USA [279] | 2019 | L.t. | 9329 | 14.5 | 45.60 | 22.60 |  |  |
| USA [280] | 2019 | 1-y | 10123 | 15.5 | 12.90 | 7.10 |  |  |
| USA [281] | 1991 | current | 6476 | 45.0 |  | 9.70 |  |  |
| USA [282] | 1998 | L.t. | 13343 | 41.5 |  |  |  | 3.9 |
| USA [283] | 2002 | L.t. | 2105 | 65.0 |  | 10.40 |  |  |
| USA [283] | 2002 | Current | 2105 | 65.0 |  |  |  |  |
| USA [144] | 2003 | 1-y | 1000 | 44.0 |  | 11.00 |  |  |
| USA [284] | 2003 | 1-y | 220 | 45.0 |  | 52.20 |  |  |
| USA [284] | 2003 | L.t. | 220 | 45.0 |  | 66.00 |  |  |
| USA [285] | 2011 | N.s. | 24749 | 14.5 |  |  |  | 3.5 |
| USA [286] | 2000 | 1-y | 4591 | 16.0 | 91.00 |  |  |  |
| USA [287] | 2001 | 1-y | 29727 | 49.0 |  | 12.20 |  |  |
| USA [288] | 2002 | 1-y | 4804 | 41.5 |  | 11.60 |  |  |
| USA [289] | 2004 | L.t. | 12750 | 60.5 | 21.20 | 6.80 |  |  |
| USA [290] | 2004 | 1-y | 8579 | 36.5 |  | 29.30 | 14.60 |  |
| USA [291] | 2006 | 1-y | 145335 | 51.5 |  | 15.00 |  |  |
| USA [292] | 2007 | 1-y | 18714 | 15.5 |  | 6.30 |  |  |
| USA [293] | 2007 | 1-y | 257399 | 48.5 | 17.40 | 16.20 |  |  |
| USA [294] | 2010 | 3-m | 208 | 11.5 | 89.50 |  |  |  |
| USA [295] | 2011 | 1-y | 5484 | 58.5 | 13.10 | 6.80 |  |  |
| USA [296] | 2012 | 2-week | 1824 | 58.0 |  |  |  |  |
| USA [297] | 2015 | N.s. | 58418 | 51.5 | 28.70 | 20.80 |  |  |
| USA [298] | 2018 | 3-m | 33672 | 51.5 |  | 15.30 |  |  |
| USA [299] | 1977 | L.t. | 1809 | 40.0 | 83.40 |  |  |  |
| USA [300] | 1989 | L.t. | 500 | 53.5 | 15.80 |  |  |  |
| USA [301] | 1989 | 1-y | 3811 | 75.0 | 45.00 | 9.50 |  |  |
| USA [302] | 1992 | 1-y | 20468 | 45.0 |  | 11.70 |  |  |
| USA [303] | 1993 | Current | 116929 | 45.0 |  | 4.10 |  |  |
| USA [304] | 1994 | L.t. | 653 | 53.0 | 21.20 |  |  |  |
| USA [304] | 1994 | 1-y | 653 | 53.0 | 13.40 | 8.50 |  |  |
| USA [305] | 1996 | 1-y | 12328 | 41.5 |  | 14.10 |  |  |
| USA [306] | 1997 | 1-y | 2572 | 9.0 |  | 8.60 |  |  |
| USA [307] | 1998 | 1-y | 13345 | 41.5 |  |  | 40.30 | 2.2 |
| USA [308] | 1997 | 1-y | 13343 | 41.5 | 59.70 | 13.30 | 31.80 |  |
| Argentina [309] | 2005 | 1-y | 517 | 50.0 |  | 5.00 |  |  |
| Brazil [310] | 1998 | 1-m | 993 | 40.5 | 52.00 | 6.10 |  |  |
| Brazil [311] | 1996 | 1-y | 280 | 24.0 | 47.10 | 41.80 |  |  |
| Brazil [312] | 2000 | N.s. | 408 | 21.0 | 34.80 |  |  |  |
| Brazil [313] | 2000 | L.t. | 1890 | 40.0 |  | 30.40 |  |  |
| Brazil [314] | 2004 | N.s. | 2500 | 41.5 | 50.60 |  |  |  |
| Brazil [315] | 2006 | 1-y | 2715 | 42.0 | 71.30 | 10.10 |  |  |
| Brazil [316] | 2006 | 1-y | 625 | 39.5 | 80.80 | 39.70 | 29.70 | 6.1 |
| Brazil [317] | 2008 | 1-y | 3848 | 48.5 | 62.20 | 13.00 | 35.30 |  |
| Brazil [318] | 2008 | 1-y | 3848 | 48.5 |  |  |  | 7.3 |
| Brazil [319] | 2009 | 1-y | 3848 | 48.5 |  | 41.50 |  |  |
| Brazil [320] | 2010 | 1-y | 1906 | 8.5 |  | 3.80 | 3.90 |  |
| Brazil [321] | 2011 | 1-y | 344 | 23.0 | 87.20 | 48.50 | 42.40 | 5.8 |
| Brazil [322] | 2011 | N.s. | 392 | 35.0 | 40.30 | 26.50 | 9.10 |  |
| Brazil [323] | 2014 | L.t. | 192 | 12.5 | 97.30 | 51.10 | 17.40 | .0 |
| Brazil [324] | 2015 | N.s. | 954 | 16.5 | 80.60 | 19.30 | 17.90 |  |
| Brazil [325] | 2015 | 1-y | 14953 | 54.5 |  | 25.90 |  |  |
| Brazil [326] | 2019 | 1-y | 3848 | 48.5 |  | 29.50 |  |  |
| Brazil [327] | 2020 | N.s. | 539 | 17.0 | 38.20 | 7.80 |  |  |
| Brazil [328] | 2020 | N.s. | 5671 | 8.5 |  | 9.60 | 12.80 |  |
| Brazil [309] | 2005 | 1-y | 857 | 50.0 |  | 12.60 |  |  |
| Brazil [329] | 2002 | 1-y | 1174 | 51.5 | 63.10 |  |  | 7.3 |
| Brazil [329] | 2002 | L.t | 1174 | 51.5 | 73.40 | 16.30 | 66.20 |  |
| Brazil [330] | 2003 | L.t | 1464 | 51.5 | 34.80 |  |  |  |
| Brazil [331] | 2008 | 1-y | 1615 | 75.0 |  | 10.50 | 32.20 |  |
| Brazil [332] | 2009 | 6-m | 102 | 49.5 | 58.40 | 34.50 |  |  |
| Brazil [333] | 2009 | 1-y | 1605 | 51.5 | 65.40 |  |  |  |
| Brazil [334] | 2010 | 1-y | 1605 | 51.5 |  |  |  | 3.6 |
| Brazil [335] | 2010 | 3-m | 1092 | 21.5 | 74.50 | 14.90 | 29.90 |  |
| Brazil [336] | 2011 | 1-y | 383 | 51.5 | 48.30 | 24.30 | 9.10 | 8.1 |
| Brazil [337] | 2015 | 6-m | 5671 | 8.5 | 79.40 |  |  |  |
| Brazil [338] | 1996 | 1-y | 538 | 14.0 | 82.90 | 9.90 | 72.30 |  |
| Brazil [338] | 1996 | L.t. | 538 | 14.0 | 93.30 |  |  |  |
| Brazil [338] | 1996 | 24 h | 538 | 14.0 | 6.00 | 1.30 | 7.60 |  |
| Brazil [339] | 1998 | 1-y | 460 | 12.0 | 90.00 |  |  |  |
| Brazil [339] | 1998 | L.t. | 460 | 12.0 | 93.50 |  |  |  |
| Chile [340] | 1997 | 1-y | 1385 | 50.0 | 36.40 | 7.30 |  |  |
| Chile [341] | 1998 | 1-y | 1385 | 50.0 |  |  | 26.70 | 2.6 |
| Colombia [342] | 2002 | N.s. | 544 | 45.0 |  | 18.30 |  |  |
| Colombia [343] | 2002 | L.t. | 1454 | 45.0 |  | 19.80 |  |  |
| Colombia [344] | 2003 | N.s. | 8910 | 45.0 |  | 6.70 |  |  |
| Colombia [345] | 2006 | L.t. | 787 | 48.0 |  | 25.10 | 43.20 |  |
| Colombia [309] | 2005 | 1-y | 688 | 50.0 |  | 9.30 |  |  |
| Colombia [346] | 2008 | L.t. | 1505 | 41.5 | 52.10 | 11.40 |  | 8.4 |
| Cuba [347] | 2009 | L.t. | 274 | 51.5 | 44.70 | 16.90 | 25.60 | 13.1 |
| Ecuador [309] | 2005 | 1-y | 336 | 50.0 |  | 8.20 |  |  |
| Ecuador [348] | 1985 | N.s. | 1113 | 45.0 | 6.70 | 5.30 |  |  |
| Ecuador [349] | 1995 | N.s. | 2723 | 45.0 | 58.00 | 6.80 |  |  |
| Mexico [309] | 2005 | 1-y | 545 | 50.0 |  | 10.00 |  |  |
| Mexico [350] | 1991 | L.t. | 700 | 47.5 | 9.80 |  |  |  |
| Panama [351] | 1988 | N.s. | 955 | 50.5 |  | 16.30 |  |  |
| Peru [352] | 1991 | N.s. | 1226 | 45.0 | 22.30 | 12.40 |  |  |
| Peru [352] | 1991 | N.s. | 1031 | 45.0 | 14.50 | 3.60 |  |  |
| Peru [353] | 1997 | 1-y | 3246 | 50.0 | 28.70 | 5.30 |  |  |
| Puerto Rico [354] | 2003 | 1-y | 1610 | 45.0 | 33.50 | 11.30 |  |  |
| Venezuela [355] | 2001 | N.s. | 1714 | 15.5 |  | 16.80 |  |  |
| Venezuela [309] | 2005 | 1-y | 634 | 50.0 |  | 8.50 |  |  |
| *A few studies (233, 237, 247249, 277, 296) only gave figures about women, and one study (175) only about men  TTH: Tension-type Headache, N: number of participants in study,H15+: Headache on ≥15 days/month, 1-y: one-year, L.t. : life-time, 24h: 24 hour, 3-,: 3-months, 6-m: 6-months, N.s.: not stated | | | | | | | | |

## References

1. Adoukonou T, Houinato D, Kankouan J, Makoutode M, Paraiso M, Tehindrazanarivelo A, Viader F and Preux PM (2009) Migraine among university students in Cotonou (Benin). Headache 49: 887-93; discussion 894.

2. Houinato D, Adoukonou T, Ntsiba F, Adjien C, Avode DG and Preux PM (2010) Prevalence of migraine in a rural community in south Benin. Cephalalgia 30: 62-7.

3. El Tallawy HN, Farghaly WM, Metwaly NA, Rageh TA, Shehata GA, Elfetoh NA, Hegazy AM, El-Moselhy EA, Rayan I, Al-Fawal BM and Abd Elhamed MA (2010) Door-to-door survey of major neurological disorders in Al Kharga District, New Valley, Egypt: methodological aspects. Neuroepidemiology 35: 185-90.

4. El-Sherbiny NA, Masoud M, Shalaby NM and Shehata HS (2015) Prevalence of primary headache disorders in Fayoum Governorate, Egypt. Journal of Headache and Pain 16: 85.

5. Kandil MR, Hamed SA, Fadel KA, Khalifa HE, Ghanem MK and Mohamed KO (2016) Migraine in Assiut Governorate, Egypt: epidemiology, risk factors, comorbid conditions and predictors of change from episodic to chronic migraine. Neurol Res 38: 232-41.

6. Tekle Haimanot R, Seraw B, Forsgren L, Ekbom K and Ekstedt J (1995) Migraine, chronic tension-type headache, and cluster headache in an Ethiopian rural community. Cephalalgia 15: 482-8.

7. Takele GM, Tekle Haimanot R and Martelletti P (2008) Prevalence and burden of primary headache in Akaki textile mill workers, Ethiopia. Journal of Headache and Pain 9: 119-28.

8. Gelaye B, Peterlin BL, Lemma S, Tesfaye M, Berhane Y and Williams MA (2013) Migraine and psychiatric comorbidities among sub-saharan african adults. Headache 53: 310-21.

9. Mengistu G and Alemayehu S (2013) Prevalence and burden of primary headache disorders among a local community in Addis Ababa, Ethiopia. Journal of Headache and Pain 14: 30.

10. Zebenigus M, Tekle-Haimanot R, Worku DK, Thomas H and Steiner TJ (2016) The prevalence of primary headache disorders in Ethiopia. Journal of Headache and Pain 17: 110.

11. Zewde YZ, Zebenigus M, Demissie H, Tekle-Haimanot R, Uluduz D, Sasmaz T, Bozdag F and Steiner TJ (2020) The prevalence of headache disorders in children and adolescents in Ethiopia: a schools-based study. Journal of Headache and Pain 21: 108.

12. Maiga Y, Soumaila B, N'Drainy Cissoko L, Sangare M, Diallo SH, Diallo S, Camara M, Sogoba Y, Coulibaly YA, Sylla F, Traore FD and Sidibe O (2017) Epidemiology of migraine among students in Mali. eNeurologicalSci 7: 32-36.

13. Ogunyemi AO (1984) Prevalence of headache among Nigerian university students. Headache 24: 127-130.

14. Matuja WB, Mteza IB and Rwiza HT (1995) Headache in a nonclinical population in Dar es Salaam, Tanzania. A community-based study. Headache 35: 273-276.

15. Ojini FI, Okubadejo NU and Danesi MA (2009) Prevalence and clinical characteristics of headache in medical students of the University of Lagos, Nigeria. Cephalalgia 29: 472-7.

16. Wahab KW and Ugheoke AJ (2009) Migraine: prevalence and associated disability among Nigerian undergraduates. Can J Neurol Sci 36: 216-21.

17. Ofovwe GE and Ofili AN (2010) Prevalence and impact of headache and migraine among secondary school students in Nigeria. Headache 50: 1570-5.

18. Ezeala-Adikaibe AB, Stella EO, Ikenna O and Ifeoma U (2012) Frequency and pattern of headache among medical students at Enugu, South East Nigeria. Niger J Med 21: 205-8.

19. Onwuekwe IO, Ezeala Adikaibe B and Ekenze SO (2012) Neurological disease burden in two semi-urban communities in South East Nigeria. Niger J Med 21: 317-9.

20. Ezeala-Adikaibe BA, Onyekonwu C, Okudo G, Onodugo O, Ekenze S, Orjioke C, Chime P, Ezeanosike O, Mbadiwe N, Chikani M, Okwara C, Ulasi I and Ijoma U (2014) Prevalence of primary headaches in an urban slum in Enugu South East Nigeria: a door-to-door survey. Headache 54: 1601-10.

21. Sanya EO, Desalu OO, Aderibigbe SA, Kolo PM, Mustapha AF and Adeyanju OA (2017) Prevalence and clinical characteristics of headaches among undergraduate students in three tertiary institutions in Ilorin, Nigeria. Niger J Clin Pract 20: 1411-1416.

22. Longe AC and Osuntokun BO (1988) Prevalence of neurological disorders in Udo, a rural community in southern Nigeria. Trop. Geogr. Med. 65: 36-40.

23. Osuntokun BO, Adeuja AO, Nottidge VA, Bademosi O, Alumide AO, Ige O, Yaria F, Schoenberg BS and Bolis CL (1992) Prevalence of headache and migrainous headache in Nigerian Africans: a community-based study. East Afr. Med. J. 69: 196-199.

24. Orji GI and Iloeje SO (1997) Childhood migraine in Nigeria--I: A community-based study. West Afr. J. Med. 16: 208-217.

25. Dent W, Spiss H, Helbok R, Matuja W, Scheunemann S and Schmutzhard E (2004) Prevalence of migraine in a rural area in South Tanzania: a door-to-door survey. Cephalalgia 24: 960-966.

26. Winkler A, Stelzhammer B, Kerschbaumsteiner K, Meindl M, Dent W, Kaaya J, Matuja W and Schmutzhard E (2009) The prevalence of headache with emphasis on tension-type headache in rural Tanzania: a community-based study. Cephalalgia 29: 1317-25.

27. Winkler AS, Dent W, Stelzhammer B, Kerschbaumsteiner K, Meindl M, Kaaya J, Matuja WB and Schmutzhard E (2010) Prevalence of migraine headache in a rural area of northern Tanzania: a community-based door-to-door survey. Cephalalgia 30: 582-92.

28. Attia Romdhane N, Ben Hamida M, Mrabet A, Larnaout A, Samoud S, Ben Hamda A, Ben Hamda M and Oueslati S (1993) Prevalence study of neurologic disorders in Kelibia (Tunisia). Neuroepidemiology 12: 285-299.

29. Kaddumukasa M, Mugenyi L, Kaddumukasa MN, Ddumba E, Devereaux M, Furlan A, Sajatovic M and Katabira E (2016) Prevalence and incidence of neurological disorders among adult Ugandans in rural and urban Mukono district; a cross-sectional study. BMC Neurol 16: 227.

30. Mbewe E, Zairemthiama P, Yeh HH, Paul R, Birbeck GL and Steiner TJ (2015) The epidemiology of primary headache disorders in Zambia: a population-based door-to-door survey. Journal of Headache and Pain 16: 515.

31. Quesada-Vazquez AJ and Rodriguez-Santana N (2006) [The prevalence of primary headaches in the working population at a psychiatric hospital in Zimbabwe]. Rev Neurol 43: 129-31.

32. Levy LM (1983) An epidemiological study of headache in an urban population in Zimbabwe. Headache 23: 2-9.

33. Zhao F, Tsay JY, Cheng XM, Wong WJ, Li SC, Yao SX, Chang SM and Schoenberg BS (1988) Epidemiology of migraine: a survey in 21 provinces of the People's Republic of China, 1985. Headache 28: 558-565.

34. Yu S, Liu R, Zhao G, Yang X, Qiao X, Feng J, Fang Y, Cao X, He M and Steiner T (2012) The prevalence and burden of primary headaches in China: a population-based door-to-door survey. Headache 52: 582-91.

35. Huang GB, Yao LT, Hou JX, Zhang ZJ, Xin YT, Wu XY, Lu GY, Chen ZQ and Huang JP (2013) Epidemiology of migraine in the She ethnic minority group in Fujian province, China. Neurol Res 35: 684-92.

36. Wong TW, Wong KS, Yu TS and Kay R (1995) Prevalence of migraine and other headaches in Hong Kong. Neuroepidemiology 14: 82-91.

37. Cheung RT (2000) Prevalence of migraine, tension-type headache, and other headaches in Hong Kong. Headache 40: 473-479.

38. Kong CK, Cheng WW and Wong LY (2001) Epidemiology of headache in Hong Kong primary-level schoolchildren: questionnaire study. Hong Kong Med. J. 7: 29-33.

39. Jin Z, Shi L, Wang YJ, Yang LG, Shi YH, Shen LW and Ren CC (2013) Prevalence of headache among children and adolescents in Shanghai, China. J Clin Neurosci 20: 117-21.

40. Yu S, He M, Liu R, Feng J, Qiao X, Yang X, Cao X, Zhao G, Fang Y and Steiner TJ (2013) Headache yesterday in China: A new approach to estimating the burden of headache, applied in a general-population survey in China. Cephalalgia 33: 1211-1217.

41. Wang X, Xing Y, Sun J, Zhou H, Yu H, Zhao Y and Yan S (2016) Prevalence, Associated Factors, and Impact on Quality of Life of Migraine in a Community in Northeast China. J Oral Facial Pain Headache 30: 139-49.

42. Zhang Y, Shi Z, Hock D, Yue W, Liu S, Zhang Y, Liu S, Zhao L, Lu H, Guan Y, Wang X, Wsiniewski T and Ji Y (2016) Prevalence of primary headache disorders in a population aged 60 years and older in a rural area of Northern China. Journal of Headache and Pain 17: 83.

43. Wang SJ, Liu HC, Fuh JL, Liu CY, Lin KP, Chen HM, Lin CH, Wang PN, Hsu LC, Wang HC and Lin KN (1997) Prevalence of headaches in a Chinese elderly population in Kinmen: age and gender effect and cross-cultural comparisons. Neurology 49: 195-200.

44. Malik AH, Shah PA and Yaseen Y (2012) Prevalence of primary headache disorders in school-going children in Kashmir Valley (North-west India). Ann Indian Acad Neurol 15: S100-3.

45. Menon B and Kinnera N (2013) Prevalence and characteristics of migraine in medical students and its impact on their daily activities. Ann Indian Acad Neurol 16: 221-5.

46. Shivpuri D, Rajesh MS and Jain D (2003) Prevalence and characteristics of migraine among adolescents: a questionnaire survey. Indian Pediatr. 40: 665-669.

47. Gourie-Devi M, Gururaj G, Satishchandra P and Subbakrishna DK (2004) Prevalence of neurological disorders in Bangalore, India: a community-based study with a comparison between urban and rural areas. Neuroepidemiology 23: 261-268.

48. Gupta R, Bhatia MS, Dahiya D, Sharma S, Sapra R, Semalti K and Dua RP (2009) Recurrent headache in Indian adolescents. Indian J Pediatr 76: 733-7.

49. Kulkarni GB, Rao GN, Gururaj G, Stovner LJ and Steiner TJ (2015) Headache disorders and public ill-health in India: prevalence estimates in Karnataka State. Journal of Headache and Pain 16: 67.

50. Steiner TJ, Rao GN, Kulkarni GB, Gururaj G and Stovner LJ (2016) Headache yesterday in Karnataka state, India: prevalence, impact and cost. Journal of Headache and Pain 17: 74.

51. Ray BK, Paul N, Hazra A, Das S, Ghosal MK, Misra AK, Banerjee TK, Chaudhuri A and Das SK (2017) Prevalence, burden, and risk factors of migraine: A community-based study from Eastern India. Neurol India 65: 1280-1288.

52. Hamzehi A, Bahrampour A and Mobasher M (1998) The evaluation of Migraine prevalence and associated demographic factors among government employees' of Kerman city. Journal of Kerman University of Medical Sciences 5: 84-91.

53. Ghorbani A, Abtahi SM, Fereidan-Esfahani M, Abtahi SH, Shemshaki H, Akbari M and Mehrabi-Koushki A (2013) Prevalence and clinical characteristics of headache among medical students, Isfahan, Iran. J Res Med Sci 18: S24-7.

54. Ayatollahi SM, Moradi F and Ayatollahi SA (2002) Prevalences of migraine and tension-type headache in adolescent girls of Shiraz (southern Iran). Headache 42: 287-90.

55. Ayatollahi SM and Khosravi A (2006) Prevalence of migraine and tension-type headache in primary-school children in Shiraz. East Mediterr Health J 12: 809-17.

56. Fallahzadeh H and Alihaydari M (2011) Prevalence of migraine and tension-type headache among school children in Yazd, Iran. J Pediatr Neurosci 6: 106-9.

57. Shahbeigi S, Fereshtehnejad SM, Mohammadi N, Golmakani MM, Tadayyon S, Jalilzadeh G and Pakdaman H (2013) Epidemiology of headaches in Tehran urban area: a population-based cross-sectional study in district 8, year 2010. Neurol Sci 34: 1157-66.

58. Rabiee B, Zeinoddini A, Kordi R, Yunesian M, Mohammadinejad P and Mansournia MA (2016) The Epidemiology of Migraine Headache in General Population of Tehran, Iran. Neuroepidemiology 46: 9-13.

59. Abramson JH, Hopp C and Epstein LM (1980) Migraine and non-migrainous headaches. A community survey in Jerusalem. J. Epidemiol. Community Health 34: 188-193.

60. Ando N, Fujimoto S, Ishikawa T, Teramoto J, Kobayashi S, Hattori A and Togari H (2007) Prevalence and features of migraine in Japanese junior high school students aged 12-15 yr. Brain Dev 29: 482-5.

61. Sakai F and Igarashi H (1997) Prevalence of migraine in Japan: a nationwide survey. Cephalalgia 17: 15-22.

62. Takeshima T, Ishizaki K, Fukuhara Y, Ijiri T, Kusumi M, Wakutani Y, Mori M, Kawashima M, Kowa H, Adachi Y, Urakami K and Nakashima K (2004) Population-based door-to-door survey of migraine in Japan: the Daisen study. Headache 44: 8-19.

63. Goto M, Yokoyama K, Nozaki Y, Itoh K, Kawamata R, Matsumoto S and Yamagata T (2017) Characteristics of headaches in Japanese elementary and junior high school students: A school-based questionnaire survey. Brain Dev 39: 791-798.

64. M AL, Al Qadire M, Aloush S, Tawalbeh L, AlAzzam M, Suliman M, Batiha AM, Alhalaiqa F, Alshakh H and Abd Al-Rahman A (2017) Assessment of Headache Among High School Students in Jordan. J Sch Nurs 1059840517734613.

65. Alzoubi KH, Mhaidat N, Azzam SA, Khader Y, Salem S, Issaifan H and Haddadin R (2009) Prevalence of migraine and tension-type headache among adults in Jordan. Journal of Headache and Pain 10: 265-70.

66. Al-Hashel JY, Ahmed SF, Alroughani R and Goadsby PJ (2014) Migraine among medical students in Kuwait University. Journal of Headache and Pain 15: 26.

67. Alders EE, Hentzen A and Tan CT (1996) A community-based prevalence study on headache in Malaysia. Headache 36: 379-384.

68. Luvsannorov O, Anisbayar T, Davaasuren M, Baatar O, Batmagnai K, Tumurbaatar K, Enkhbaatar S, Uluduz D, Sasmaz T, Solmaz ET and Steiner TJ (2020) The prevalence of headache disorders in children and adolescents in Mongolia: a nationwide schools-based study. Journal of Headache and Pain 21: 107.

69. Manandhar K, Risal A, Steiner TJ, Holen A and Linde M (2015) The prevalence of primary headache disorders in Nepal: a nationwide population-based study. Journal of Headache and Pain 16: 95.

70. Deleu D, Khan MA, Humaidan H, Al Mantheri Z and Al Hashami S (2001) Prevalence and clinical characteristics of headache in medical students in oman. Headache 41: 798-804.

71. Deleu D, Khan MA and Al Shehab TA (2002) Prevalence and clinical characteristics of headache in a rural community in oman. Headache 42: 963-973.

72. Siddiqui SJ, Shamim SM and Hashmi AM (2006) Prevalence and patterns of headache in school children in Karachi. J Pak Med Assoc 56: 215-7.

73. Herekar AA, Ahmad A, Uqaili UL, Ahmed B, Effendi J, Alvi SZ, Shahab MA, Javed U, Herekar AD, Khanani R and Steiner TJ (2017) Primary headache disorders in the adult general population of Pakistan - a cross sectional nationwide prevalence survey. Journal of Headache and Pain 18: 28.

74. Bener A (2006) Frequency of headache and migraine in Qatar. Neuroepidemiology 27: 61-6.

75. Bessisso MS, Bener A, Elsaid MF, Al-Khalaf FA and Huzaima KA (2005) Pattern of headache in school children in the State of Qatar. Saudi Med J 26: 566-70.

76. Song TJ, Cho SJ, Kim WJ, Yang KI, Yun CH and Chu MK (2019) Sex Differences in Prevalence, Symptoms, Impact, and Psychiatric Comorbidities in Migraine and Probable Migraine: A Population-Based Study. Headache 59: 215-223.

77. Al-Tulaihi BA and Al-Jumah MA (2009) Prevalence of migraine and non-migraine headache among high school students at the National Guard Housing in Riyadh, Saudi Arabia. Saudi Med J 30: 120-4.

78. Muayqil T, Al-Jafen BN, Al-Saaran Z, Al-Shammari M, Alkthiry A, Muhammad WS, Murshid R and Alanazy MH (2018) Migraine and Headache Prevalence and Associated Comorbidities in a Large Saudi Sample. Eur Neurol 79: 126-134.

79. Desouky DE, Zaid HA and Taha AA (2019) Migraine, tension-type headache, and depression among Saudi female students in Taif University. J Egypt Public Health Assoc 94: 7.

80. Al Jumah M, Al Khathaami AM, Kojan S, Hussain M, Thomas H and Steiner TJ (2020) The prevalence of primary headache disorders in Saudi Arabia: a cross-sectional population-based study. Journal of Headache and Pain 21: 11.

81. Al Jumah M, Awada A and Al Azzam S (2002) Headache syndromes amongst schoolchildren in Riyadh, Saudi Arabia. Headache 42: 281-6.

82. al Rajeh S, Bademosi O, Ismail H, Awada A, Dawodu A, al-Freihi H, Assuhaimi S, Borollosi M and al-Shammasi S (1993) A community survey of neurological disorders in Saudi Arabia: the Thugbah study. Neuroepidemiology 12: 164-78.

83. Abduljabbar M, Ogunniyi A, al Balla S, Alballaa S and al-Dalaan A (1996) Prevalence of primary headache syndrome in adults in the Qassim region of Saudi Arabia. Headache 36: 385-388.

84. Ho KH, Ong BK and Lee SC (1997) Headache and self-assessed depression scores in Singapore University undergraduates. Headache 37: 26-30.

85. Jeyagurunathan A, Abdin E, Vaingankar JA, Chua BY, Shafie S, Chang SHS, James L, Tan KB, Basu S, Chong SA and Subramaniam M (2020) Prevalence and comorbidity of migraine headache: results from the Singapore Mental Health Study 2016. Soc Psychiatry Psychiatr Epidemiol 55: 33-43.

86. Ho KH and Ong BK (2003) A community-based study of headache diagnosis and prevalence in Singapore. Cephalalgia 23: 6-13.

87. Chong SC, Chan YH, Ong HT, Low PS and Tay SK (2010) Headache diagnosis, disability and co-morbidities in a multi-ethnic, heterogeneous paediatric Asian population. Cephalalgia 30: 953-61.

88. Kim BK, Chung YK, Kim JM, Lee KS and Chu MK (2013) Prevalence, clinical characteristics and disability of migraine and probable migraine: a nationwide population-based survey in Korea. Cephalalgia 33: 1106-16.

89. Kim BK, Chu MK, Lee TG, Kim JM, Chung CS and Lee KS (2012) Prevalence and impact of migraine and tension-type headache in Korea. J Clin Neurol 8: 204-11.

90. Roh JK, Kim JS and Ahn YO (1998) Epidemiologic and clinical characteristics of migraine and tension-type headache in Korea. Headache 38: 356-365.

91. Wang SJ, Fuh JL, Juang KD and Lu SR (2005) Rising prevalence of migraine in Taiwanese adolescents aged 13-15 years. Cephalalgia 25: 433-438.

92. Wang SJ, Fuh JL, Lu SR and Juang KD (2006) Chronic daily headache in adolescents. Prevalence, impact, and medication overuse. Neurology 66: 193-197.

93. Lu SR, Wang SJ and Fuh JL (2006) The practice pattern of migraine management among neurologists in Taiwan. Cephalalgia 26: 310-3.

94. Fuh JL, Wang SJ, Lu SR, Liao YC, Chen SP and Yang CY (2010) Headache disability among adolescents: a student population-based study. Headache 50: 210-8.

95. Wang SJ, Fuh JL, Young YH, Lu SR and Shia BC (2000) Prevalence of migraine in Taipei, Taiwan: a population-based survey. Cephalalgia 20: 566-572.

96. Lu SR, Fuh JL, Juang KD and Wang SJ (2000) Migraine prevalence in adolescents aged 13-15: a student population-based study in Taiwan. Cephalalgia 20: 479-485.

97. Lu SR, Fuh JL, Chen WT, Juang KD and Wang SJ (2001) Chronic daily headache in Taipei, Taiwan: prevalence, follow-up and outcome predictors. Cephalalgia 21: 980-986.

98. Visudtibhan A, Siripornpanich V, Khongkhatithum C, Chiemchanya S, Sirijunpen S, Ruangkanchanasetr S and Visudhiphan P (2007) Migraine in Thai children: prevalence in junior high school students. J Child Neurol 22: 1117-20.

99. Visudtibhan A, Boonsopa C, Thampratankul L, Nuntnarumit P, Okaschareon C, Khongkhatithum C, Chiemchanya S and Visudhiphan P (2010) Headache in junior high school students: types & characteristics in Thai children. J Med Assoc Thai 93: 550-7.

100. Phanthumchinda K and Sithi-Amorn C (1989) Prevalence and clinical features of migraine: a community survey in Bangkok, Thailand. Headache 29: 594-597.

101. Srikiatkhachorn A (1991) Epidemiology of headache in the Thai elderly: a study in the Bangkae Home for the Aged. Headache 31: 677-681.

102. Bener A, Swadi H, Qassimi EMA and Uduman S (1998) Prevalence of headache and migraine in schoolchildren in the United Arab Emirates. Ann. Saudi Med. 18: 522-524.

103. Abdo SA, Amood Al-Kamarany M, Alzoubi KH, Al-Maktari MT and Al-Baidani AH (2014) Primary headache in yemen: prevalence and common medications used. Neurol Res Int 2014: 808126.

104. King NJ and Sharpley CF (1990) Headache activity in children and adolescents. J. Paediatr. Child Health 26: 50-54.

105. Wilkinson IA, Halliday JA, Henry RL, Hankin RG and Hensley MJ (1994) Headache and asthma. J. Paediatr. Child Health 30: 253-256.

106. Mitchell P, Wang JJ, Currie J, Cumming RG and Smith W (1998) Prevalence and vascular associations with migraine in older Australians. Aust N Z J Med 28: 627-632.

107. Waldie KE, Thompson JM, Mia Y, Murphy R, Wall C and Mitchell EA (2014) Risk factors for migraine and tension-type headache in 11 year old children. Journal of Headache and Pain 15: 60.

108. Waldie KE and Poulton R (2002) The burden of illness associated with headache disorders among young adults in a representative cohort study. Headache 42: 612-619.

109. Paulin JM, Waal-Manning HJ, Simpson FO and Knight RG (1985) The prevalence of headache in a small New Zealand town. Headache 25: 147-151.

110. Thomson AN, White GE and West R (1993) The prevalence of bad headaches including migraine in a multiethnic community. N. Z. Med. J. 106: 477-480.

111. Steiner TJ, Stovner LJ, Katsarava Z, Lainez JM, Lampl C, Lanteri-Minet M, Rastenyte D, Ruiz de la Torre E, Tassorelli C, Barre J and Andree C (2014) The impact of headache in Europe: principal results of the Eurolight project. Journal of Headache and Pain 15: 31.

112. Kruja J, Beghi E, Zerbi D, Dobi D, Kuqo A, Zekja I, Mijo S, Kapisyzi M and Messina P (2012) High prevalence of major neurological disorders in two Albanian communities: results of a door-to-door survey. Neuroepidemiology 38: 138-47.

113. Philipp J, Zeiler M, Wober C, Wagner G, Karwautz AFK, Steiner TJ and Wober-Bingol C (2019) Prevalence and burden of headache in children and adolescents in Austria - a nationwide study in a representative sample of pupils aged 10-18 years. Journal of Headache and Pain 20: 101.

114. Lampl C, Buzath A, Baumhackl U and Klingler D (2003) One-year prevalence of migraine in Austria: a nation-wide survey. Cephalalgia 23: 280-286.

115. Moens G, Johannik K, Verbeek C and Bulterys S (2007) The prevalence and characteristics of migraine among the Belgian working population. Acta Neurol Belg 107: 84-90.

116. Gerardy PY, Fumal A and Schoenen J (2008) [Epidemiology and economic repercussion of headache: an inquiery among the administrative and technical personnel of the Liege University]. Rev Med Liege 63: 310-4.

117. Pacheva I, Milanov I, Ivanov I and Stefanov R (2012) Evaluation of diagnostic and prognostic value of clinical characteristics of migraine and tension type headache included in the diagnostic criteria for children and adolescents in International Classification of Headache Disorders--second edition. Int J Clin Pract 66: 1168-77.

118. Galinovic I, Vukovic V, Troselj M, Antic S and Demarin V (2009) Migraine and tension-type headache in medical students: a questionnaire study. Coll Antropol 33: 169-73.

119. Zivadinov R, Willheim K, Jurjevic A, Sepic-Grahovac D, Bucuk M and Zorzon M (2001) Prevalence of migraine in Croatia: a population-based survey. Headache 41: 805-812.

120. Zivadinov R, Willheim K, Sepic-Grahovac D, Jurjevic A, Bucuk M, Brnabic-Razmilic O, Relja G and Zorzon M (2003) Migraine and tension-type headache in Croatia: a population-based survey of precipitating factors. Cephalalgia 23: 336-343.

121. Vukovic-Cvetkovic V, Plavec D and Lovrencic-Huzjan A (2013) Prevalence of chronic headache in Croatia. Biomed Res Int 2013: 837613.

122. Vukovic V, Plavec D, Pavelin S, Janculjak D, Ivankovic M and Demarin V (2010) Prevalence of migraine, probable migraine and tension-type headache in the Croatian population. Neuroepidemiology 35: 59-65.

123. Cvetkovic VV, Plavec D, Lovrencic-Huzjan A, Strineka M, Azman D and Bene R (2014) Prevalence and clinical characteristics of headache in adolescents: a Croatian epidemiological study. Cephalalgia 34: 289-97.

124. Sedlic M, Mahovic D and Kruzliak P (2016) Epidemiology of Primary Headaches Among 1,876 Adolescents: A Cross-Sectional Survey. Pain Med 17: 353-9.

125. Russell MB, Rasmussen BK, Thorvaldsen P and Olesen J (1995) Prevalence and sex-ratio of the subtypes of migraine. Int. J. Epidemiol. 15: 612-618.

126. Russell MB, Ulrich V, Gervil M and Olesen J (2002) Migraine without aura and migraine with aura are distinct disorders. A population-based twin survey. Headache 42: 332-6.

127. Le H, Tfelt-Hansen P, Skytthe A, Kyvik KO and Olesen J (2012) Increase in self-reported migraine prevalence in the Danish adult population: a prospective longitudinal population-based study. BMJ Open 2: .

128. Ashina S, Bendtsen L, Lyngberg AC, Lipton RB, Hajiyeva N and Jensen R (2015) Prevalence of neck pain in migraine and tension-type headache: a population study. Cephalalgia 35: 211-9.

129. Westergaard ML, Lau CJ, Allesoe K, Gjendal ST and Jensen RH (2020) Monitoring chronic headache and medication-overuse headache prevalence in Denmark. Cephalalgia 40: 6-18.

130. Lyngberg AC, Rasmussen BK, Jorgensen T and Jensen R (2005) Has the prevalence of migraine and tension-type headache changed over a 12-year period? A Danish population survey. Eur. J. Epidemiol. 20: 243-249.

131. Westergaard ML, Glumer C, Hansen EH and Jensen RH (2014) Prevalence of chronic headache with and without medication overuse: associations with socioeconomic position and physical and mental health status. Pain 155: 2005-13.

132. Rasmussen BK, Jensen R, Schroll M and Olesen J (1991) Epidemiology of headache in a general population--a prevalence study. J. Clin. Epidemiol. 44: 1147-1157.

133. Toom K, Raidvee A, Allas KH, Floria E, Juhkami K, Klimusev G, Leping M, Liidemann M, Milovidov A, Liivak K, Paiste S, Paju G, Uhs K, Vaikjarv M, Veetousme K, Valja P and Braschinsky M (2019) The prevalence of primary headache disorders in the adult population of Estonia. Cephalalgia 39: 883-891.

134. Sillanpaa M (1976) Prevalence of migraine and other headache in Finnish children starting school. Headache 15: 288-90.

135. Sillanpää M (1983) Changes in the prevalence of migraine and other headaches during the first seven school years. Headache 23: 15-19.

136. Honkasalo ML, Kaprio J, Heikkila K, Sillanpaa M and Koskenvuo M (1993) A population-based survey of headache and migraine in 22,809 adults. Headache 33: 403-412.

137. Sillanpaa M and Anttila P (1996) Increasing prevalence of headache in 7-year-old schoolchildren. Headache 36: 466-470.

138. Virtanen R, Aromaa M, Koskenvuo M, Sillanpaa M, Rose RJ, Metsahonkala L, Helenius H, Anttila P and Kaprio J (2009) Prevalence and incidence of headache in adolescent Finnish twins. Headache 49: 1503-12.

139. Nikiforow R (1981) Headache in a random sample of 200 persons: a clinical study of a population in northern Finland. Cephalalgia 1: 99-107.

140. Sillanpää M (1983) Prevalence of headache in prepuberty. Headache 23: 10-14.

141. Sillanpaa M, Piekkala P and Kero P (1991) Prevalence of headache at preschool age in an unselected child population. Cephalalgia 11: 239-242.

142. Metsähonkala L and Sillanpää M (1994) Migraine in children--an evaluation of the IHS criteria. Cephalalgia 14: 285-290.

143. Munoz M, Dumas M, Boutros-Toni F, Coquelle D, Nicolas A, Ndzanga E, Boa F, Vallat JM and Dartigues JF (1993) Prevalence of headache in a representative sample of the population in a French department (Haute-Vienne-Limousin). Headache 33: 521-523.

144. MacGregor EA, Brandes J and Eikermann A (2003) Migraine prevalence and treatment patterns: the global Migraine and Zolmitriptan Evaluation survey. Headache 43: 19-26.

145. Donnet A, Becker H, Allaf B and Lanteri-Minet M (2010) Migraine and migraines of specialists: perceptions and management. Headache 50: 1115-25.

146. Kurth T, Mohamed S, Maillard P, Zhu YC, Chabriat H, Mazoyer B, Bousser MG, Dufouil C and Tzourio C (2011) Headache, migraine, and structural brain lesions and function: population based Epidemiology of Vascular Ageing-MRI study. BMJ 342: c7357.

147. Henry P, Auray JP, Gaudin AF, Dartigues JF, Duru G, Lanteri-Minet M, Lucas C, Pradalier A, Chazot G and El Hasnaoui A (2002) Prevalence and clinical characteristics of migraine in France. Neurology 59: 232-237.

148. Michel P, Pariente P, Duru G, Dreyfus JP, Chabriat H, Henry P and Dreyfuss JP (1996) MIG ACCESS: a population-based, nationwide, comparative survey of access to care in migraine in France. Cephalalgia 16: 50-55.

149. Lanteri-Minet M, Auray JP, El Hasnaoui A, Dartigues JF, Duru G, Henry P, Lucas C, Pradalier A, Chazot G and Gaudin AF (2003) Prevalence and description of chronic daily headache in the general population in France. Pain 102: 143-9.

150. Lanteri-Minet M, Valade D, Geraud G, Chautard MH and Lucas C (2005) Migraine and probable migraine--results of FRAMIG 3, a French nationwide survey carried out according to the 2004 IHS classification. Cephalalgia 25: 1146-58.

151. Henry P, Michel P, Brochet B, Dartigues JF, Tison S and Salamon R (1992) A nationwide survey of migraine in France: prevalence and clinical features in adults. Cephalalgia 12: 229-237.

152. Katsarava Z, Kukava M, Mirvelashvili E, Tavadze A, Dzagnidze A, Djibuti M and Steiner TJ (2007) A pilot methodological validation study for a population-based survey of the prevalences of migraine, tension-type headache and chronic daily headache in the country of Georgia. Journal of Headache and Pain 8: 77-82.

153. Katsarava Z, Dzagnidze A, Kukava M, Mirvelashvili E, Djibuti M, Janelidze M, Jensen R, Stovner LJ and Steiner TJ (2009) Primary headache disorders in the Republic of Georgia: Prevalence and risk factors. Neurology 73: 1796-1803.

154. Koehler T, Buck-Emden E and Dulz K (1992) Frequency of migraine among an unselected group of employees and variation of prevalence according to different diagnostic criteria. Headache 32: 79-83.

155. Pothmann R, Frankenberg SV, Muller B, Sartory G and Hellmeier W (1994) Epidemiology of headache in children and adolescents: evidence of high prevalence of migraine. Int J Behav Med 1: 76-89.

156. Obermann M, Yoon MS, Dommes P, Kuznetsova J, Maschke M, Weimar C, Limmroth V, Diener HC and Katsarava Z (2007) Prevalence of trigeminal autonomic symptoms in migraine: a population-based study. Cephalalgia 27: 504-9.

157. Gassmann J, Morris L, Heinrich M and Kroner-Herwig B (2008) One-year course of paediatric headache in children and adolescents aged 8-15 years. Cephalalgia 28: 1154-62.

158. Pfaffenrath V, Fendrich K, Vennemann M, Meisinger C, Ladwig KH, Evers S, Straube A, Hoffmann W and Berger K (2009) Regional variations in the prevalence of migraine and tension-type headache applying the new IHS criteria: the German DMKG Headache Study. Cephalalgia 29: 48-57.

159. Ratcliffe GE, Enns MW, Jacobi F, Belik SL and Sareen J (2009) The relationship between migraine and mental disorders in a population-based sample. Gen Hosp Psychiatry 31: 14-9.

160. Milde-Busch A, Boneberger A, Heinrich S, Thomas S, Kuhnlein A, Radon K, Straube A and von Kries R (2010) Higher prevalence of psychopathological symptoms in adolescents with headache. A population-based cross-sectional study. Headache 50: 738-48.

161. Blaschek A, Decke S, Albers L, Schroeder AS, Lehmann S, Straube A, Landgraf MN, Heinen F and von Kries R (2014) Self-reported neck pain is associated with migraine but not with tension-type headache in adolescents. Cephalalgia 34: 895-903.

162. Albers L, Straube A, Landgraf MN, Filippopulos F, Heinen F and von Kries R (2015) Migraine and tension type headache in adolescents at grammar school in Germany - burden of disease and health care utilization. Journal of Headache and Pain 16: 534.

163. Nieswand V, Richter M, Berner R, von der Hagen M, Klimova A, Roeder I, Koch T, Sabatowski R and Gossrau G (2019) The prevalence of headache in German pupils of different ages and school types. Cephalalgia 39: 1030-1040.

164. A R-I, U T, Hh R, H S, x000F, ven and P S (2004) Reports of pain among German children and adolescents: an epidemiological study. Acta Paediatrica 93: 258-263.

165. Fendrich K, Vennemann M, Pfaffenrath V, Evers S, May A, Berger K and Hoffmann W (2007) Headache prevalence among adolescents--the German DMKG headache study. Cephalalgia 27: 347-54.

166. Kroner-Herwig B, Heinrich M and Morris L (2007) Headache in German children and adolescents: a population-based epidemiological study. Cephalalgia 27: 519-27.

167. Radtke A and Neuhauser H (2009) Prevalence and burden of headache and migraine in Germany. Headache 49: 79-89.

168. Heinrich M, Morris L and Kroner-Herwig B (2009) Self-report of headache in children and adolescents in Germany: possibilities and confines of questionnaire data for headache classification. Cephalalgia 29: 864-72.

169. Straube A, Pfaffenrath V, Ladwig KH, Meisinger C, Hoffmann W, Fendrich K, Vennemann M and Berger K (2010) Prevalence of chronic migraine and medication overuse headache in Germany--the German DMKG headache study. Cephalalgia 30: 207-13.

170. Milde-Busch A, Blaschek A, Heinen F, Borggrafe I, Koerte I, Straube A, Schankin C and von Kries R (2011) Associations between stress and migraine and tension-type headache: results from a school-based study in adolescents from grammar schools in Germany. Cephalalgia 31: 774-85.

171. Du Y, Knopf H, Zhuang W and Ellert U (2011) Pain perceived in a national community sample of German children and adolescents. Eur J Pain 15: 649-57.

172. Yoon MS, Katsarava Z, Obermann M, Fritsche G, Oezyurt M, Kaesewinkel K, Katsarova A, Santowski I, Diener H and Moebus S (2012) Prevalence of primary headaches in Germany: results of the German Headache Consortium Study. Journal of Headache and Pain 13: 215-23.

173. Straube A, Aicher B, Forderreuther S, Eggert T, Koppel J, Moller S, Schneider R and Haag G (2013) Period prevalence of self-reported headache in the general population in Germany from 1995-2005 and 2009: results from annual nationwide population-based cross-sectional surveys. Journal of Headache and Pain 14: 11.

174. Gobel H, Petersen-Braun M and Soyka D (1994) The epidemiology of headache in Germany: a nationwide survey of a representative sample on the basis of the headache classification of the International Headache Society. Cephalalgia 14: 97-106.

175. Mitsikostas DD, Thomas A, Gatzonis S, Ilias A and Papageorgiou C (1994) An epidemiological study of headache among the Monks of Athos (Greece). Headache 34: 539-41.

176. Mitsikostas DD, Tsaklakidou D, Athanasiadis N and Thomas A (1996) The prevalence of headache in Greece: correlations to latitude and climatological factors. Headache 36: 168-173.

177. Mavromichalis I, Anagnostopoulos D, Metaxas N and Papanastassiou E (1999) Prevalence of migraine in schoolchildren and some clinical comparisons between migraine with and without aura. Headache 39: 728-736.

178. Bank J and Marton S (2000) Hungarian migraine epidemiology. Headache 40: 164-169.

179. Kobor J, Nyari T, Benedek G and Turi S (2013) Age-related prevalence and features of migraine headache in Hungarian schoolchildren and adolescents. Eur J Paediatr Neurol 17: 600-7.

180. Bruni O, Russo PM, Ferri R, Novelli L, Galli F and Guidetti V (2008) Relationships between headache and sleep in a non-clinical population of children and adolescents. Sleep Med 9: 542-8.

181. Valentinis L, Valent F, Mucchiut M, Barbone F, Bergonzi P and Zanchin G (2009) Migraine in adolescents: validation of a screening questionnaire. Headache 49: 202-11.

182. Ferrante T, Manzoni GC, Russo M, Taga A, Camarda C, Veronesi L, Pasquarella C, Sansebastiano G and Torelli P (2015) The PACE study: past-year prevalence of tension-type headache and its subtypes in Parma's adult general population. Neurol Sci 36: 35-42.

183. Taga A, Russo M, Manzoni GC and Torelli P (2017) The PACE study: lifetime and past-year prevalence of headache in Parma's adult general population. Neurol Sci 38: 789-795.

184. Foiadelli T, Piccorossi A, Sacchi L, De Amici M, Tucci M, Brambilla I, Marseglia GL, Savasta S and Verrotti A (2018) Clinical characteristics of headache in Italian adolescents aged 11-16 years: a cross-sectional questionnaire school-based study. Ital J Pediatr 44: 44.

185. Prencipe M, Casini AR, Ferretti C, Santini M, Pezzella F, Scaldaferri N and Culasso F (2001) Prevalence of headache in an elderly population: attack frequency, disability, and use of medication. J. Neurol. Neurosurg. Psychiatry 70: 377-381.

186. Camarda R and Monastero R (2003) Prevalence of primary headaches in Italian elderly: preliminary data from the Zabut Aging Project. Neurolog. Sci. 24 Suppl 2: S122-S124.

187. Raieli V, Raimondo D, Cammalleri R and Camarda R (1995) Migraine headaches in adolescents: a student population-based study in Monreale. Cephalalgia 15: 5-12.

188. Schwaiger J, Kiechl S, Seppi K, Sawires M, Stockner H, Erlacher T, Mairhofer ML, Niederkofler H, Rungger G, Gasperi A, Poewe W and Willeit J (2009) Prevalence of primary headaches and cranial neuralgias in men and women aged 55-94 years (Bruneck Study). Cephalalgia 29: 179-87.

189. Santinello M, Vieno A and De Vogli R (2009) Primary headache in Italian early adolescents: the role of perceived teacher unfairness. Headache 49: 366-74.

190. Pogliani L, Spiri D, Penagini F, Nello FD, Duca P and Zuccotti GV (2011) Headache in children and adolescents aged 6-18 years in northern Italy: prevalence and risk factors. Eur J Paediatr Neurol 15: 234-40.

191. Ferrante T, Castellini P, Abrignani G, Latte L, Russo M, Camarda C, Veronesi L, Pasquarella C, Manzoni GC and Torelli P (2012) The PACE study: past-year prevalence of migraine in Parma's adult general population. Cephalalgia 32: 358-65.

192. Cavestro C, Montrucchio F, Benci P, Pompilio D, Mandrino S, Cencio PG, Frigeri MC and Di Pietrantonj C (2014) Headache prevalence and related symptoms, family history, and treatment habits in a representative population of children in Alba, Italy. Pediatr Neurol 51: 348-53.

193. Allena M, Steiner TJ, Sances G, Carugno B, Balsamo F, Nappi G, Andree C and Tassorelli C (2015) Impact of headache disorders in Italy and the public-health and policy implications: a population-based study within the Eurolight Project. Journal of Headache and Pain 16: 100.

194. Rastenyte D, Mickeviciene D, Stovner LJ, Thomas H, Andree C and Steiner TJ (2017) Prevalence and burden of headache disorders in Lithuania and their public-health and policy implications: a population-based study within the Eurolight Project. Journal of Headache and Pain 18: 53.

195. Genc D, Vaiciene-Magistris N, Zaborskis A, Sasmaz T, Tunc AY, Uluduz D and Steiner TJ (2020) The prevalence of headache disorders in children and adolescents in Lithuania: a schools-based study. Journal of Headache and Pain 21: 73.

196. Launer LJ, Terwindt GM and Ferrari MD (1999) The prevalence and characteristics of migraine in a population-based cohort: the GEM study. Neurology 53: 537-542.

197. Hagen K, Zwart JA, Aamodt AH, Nilsen KB, Brathen G, Helde G, Stjern M, Tronvik EA and Stovner LJ (2008) A face-to-face interview of participants in HUNT 3: the impact of the screening question on headache prevalence. Journal of Headache and Pain 9: 289-94.

198. Linde M, Stovner LJ, Zwart JA and Hagen K (2011) Time trends in the prevalence of headache disorders. The Nord-Trondelag Health Studies (HUNT 2 and HUNT 3). Cephalalgia 31: 585-96.

199. Alstadhaug KB, Hernandez A, Naess H and Stovner LJ (2012) Migraine among Norwegian neurologists. Headache 52: 1369-76.

200. Hartberg S, Clench-Aas J, Raanaas RK and Lundqvist C (2015) Coping strategies among adolescents with chronic headache and mental health problems: a cross-sectional population-based study. Springerplus 4: 801.

201. Hagen K, Asberg AN, Uhlig BL, Tronvik E, Brenner E, Stjern M, Helde G, Gravdahl GB and Sand T (2018) The epidemiology of headache disorders: a face-to-face interview of participants in HUNT4. Journal of Headache and Pain 19: 25.

202. Hagen K, Hopstock LA, Elise Eggen A, Mathiesen EB and Nilsen KB (2019) Does insomnia modify the association between C-reactive protein and migraine? The Tromso Study 2015-2016. Cephalalgia 39: 1022-1029.

203. Hagen K, Stovner LJ and Zwart JA (2020) Time trends of major headache diagnoses and predictive factors. Data from three Nord-Trondelag health surveys. Journal of Headache and Pain 21: 24.

204. Zwart JA, Dyb G, Holmen TL, Stovner LJ and Sand T (2004) The prevalence of migraine and tension-type headaches among adolescents in Norway. The Nord-Trondelag Health Study (Head-HUNT-Youth), a large population-based epidemiological study. Cephalalgia 24: 373-379.

205. Hagen K, Zwart JA, Vatten L, Stovner LJ and Bovim G (2000) Prevalence of migraine and non-migrainous headache--head-HUNT, a large population-based study. Cephalalgia 20: 900-906.

206. Russell MB, Kristiansen HA, Saltyte-Benth J and Kvaerner KJ (2008) A cross-sectional population-based survey of migraine and headache in 21,177 Norwegians: the Akershus sleep apnea project. Journal of Headache and Pain 9: 339-47.

207. Grande RB, Aaseth K, Gulbrandsen P, Lundqvist C and Russell MB (2008) Prevalence of primary chronic headache in a population-based sample of 30- to 44-year-old persons. The Akershus study of chronic headache. Neuroepidemiology 30: 76-83.

208. Vetvik KG, MacGregor EA, Lundqvist C and Russell MB (2010) Self-reported menstrual migraine in the general population. Journal of Headache and Pain 11: 87-92.

209. Krogh AB, Larsson B and Linde M (2015) Prevalence and disability of headache among Norwegian adolescents: A cross-sectional school-based study. Cephalalgia 35: 1181-91.

210. Krasnik A (1999) [Headaches in the population of school children in Poznan]. Neurol Neurochir Pol 33 Suppl 5: 111-25.

211. Split W and Neuman W (1999) Epidemiology of migraine among students from randomly selected secondary schools in Lodz. Headache 39: 494-501.

212. Neuman W and Split W (1999) [Spontaneous headaches among secondary school students]. Neurol Neurochir Pol 33 Suppl 5: 97-109.

213. Monteiro JM, Matos E and Calheiros JM (1994) Headaches in medical school students. Neuroepidemiology 13: 103-7.

214. Pereira Monteiro J (1995) Cefaleias: Estudo Epidemiologico e clinico da uma populacao urbana (Thesis). 306.

215. Ayzenberg I, Katsarava Z, Sborowski A, Chernysh M, Osipova V, Tabeeva G, Yakhno N, Steiner TJ and Lifting the B (2012) The prevalence of primary headache disorders in Russia: a countrywide survey. Cephalalgia 32: 373-81.

216. Ayzenberg I, Katsarava Z, Sborowski A, Obermann M, Chernysh M, Osipova V, Tabeeva G and Steiner TJ (2014) Headache yesterday in Russia: its prevalence and impact, and their application in estimating the national burden attributable to headache disorders. Journal of Headache and Pain 15: 7.

217. Lebedeva ER, Kobzeva NR, Gilev D and Olesen J (2016) Prevalence of primary headache disorders diagnosed according to ICHD-3 beta in three different social groups. Cephalalgia 36: 579-88.

218. D'Alessandro R, Benassi G, Lenzi PL, Gamberini G, Sacquegna T, De Carolis P and Lugaresi E (1988) Epidemiology of headache in the Republic of San Marino. J Neurol Neurosurg Psychiatry 51: 21-27.

219. Milovanovic M, Jarebinski M and Martinovic Z (2007) Prevalence of primary headaches in children from Belgrade, Serbia. Eur J Paediatr Neurol 11: 136-41.

220. Klemenc-Ketis Z, Krizmaric M and Kersnik J (2013) Age- and gender-specific prevalence of self-reported symptoms in adults. Cent Eur J Public Health 21: 160-4.

221. Laínez MJA, Vioque J, Hernández-Aguado I and Titus F (1994) Prevalence of migraine in Spain. An assessment of the questionnaire's validity by clinical interview. In: (editors). Frontiers in Headache Research: Headache Classification and Epidemiology.^New York:pp 221-225..

222. Muniz R, Macia C, Montiel I, Gonzalez O, Martin R, Asensio M and Matias-Guiu J (1995) [Prevalence of migraine in the medical student population as determined by means of the 'Alcoi 1992' questionnaire]. Rev Neurol 23: 870-3.

223. Sanchez-Perez R, Asensio M, Melchor A, Montiel I, Falip R, Molto JM and Matias-Guiu J (1999) [A descriptive study of migraine in a rural population of Area del Comtat]. Rev Neurol 28: 373-6.

224. Fernandez-de-las-Penas C, Palacios-Cena D, Salom-Moreno J, Lopez-de-Andres A, Hernandez-Barrera V, Jimenez-Trujillo I, Jimenez-Garcia R, Gallardo-Pino C, Garcia-Gomez-de-las-Heras MS and Carrasco-Garrido P (2014) Has the prevalence of migraine changed over the last decade (2003-2012)? A Spanish population-based survey. PLoS One 9: e110530.

225. Jimenez-Sanchez S, Fernandez-de-las-Penas C, Jimenez-Garcia R, Hernandez-Barrera V, Alonso-Blanco C, Palacios-Cena D and Carrasco-Garrido P (2013) Prevalence of migraine headaches in the Romany population in Spain: sociodemographic factors, lifestyle and co-morbidity. J Transcult Nurs 24: 6-13.

226. Torres-Ferrus M, Vila-Sala C, Quintana M, Ajanovic S, Gallardo VJ, Gomez JB, Alvarez-Sabin J, Macaya A and Pozo-Rosich P (2019) Headache, comorbidities and lifestyle in an adolescent population (The TEENs Study). Cephalalgia 39: 91-99.

227. Bassols Farres A, Bosch-Llonch F, Campillo-Grau M and Banos-Diez JE (2002) [An epidemiologic study of headache and its treatment in the general population of Catalonia]. Rev Neurol 34: 901-8.

228. Matias-Guiu J, Porta-Etessam J, Mateos V, Diaz-Insa S, Lopez-Gil A, Fernandez C and Scientific Committee of the PP (2011) One-year prevalence of migraine in Spain: a nationwide population-based survey. Cephalalgia 31: 463-70.

229. Palencia R and Sinovas MI (1997) [Prevalence of migraine in a sample population of school children]. Rev Neurol 25: 1879-82.

230. Castillo J, Munoz P, Guitera V and Pascual J (1999) Epidemiology of Chronic Daily Headache in the General Population. Headache 39: 190-196.

231. Larsson B and Fichtel A (2014) Headache prevalence and characteristics among adolescents in the general population: a comparison between retrospect questionnaire and prospective paper diary data. Journal of Headache and Pain 15: 80.

232. Dahlof C and Linde M (2001) One-year prevalence of migraine in Sweden: a population-based study in adults. Cephalalgia 21: 664-671.

233. Mattsson P, Svardsudd K, Lundberg PO and Westerberg CE (2000) The prevalence of migraine in women aged 40-74 years: a population-based study. Cephalalgia 20: 893-899.

234. Laurell K, Larsson B and Eeg-Olofsson O (2004) Prevalence of headache in Swedish schoolchildren, with a focus on tension-type headache. Cephalalgia 24: 380-388.

235. Brun Sundblad GM, Saartok T and Engstrom LM (2007) Prevalence and co-occurrence of self-rated pain and perceived health in school-children: Age and gender differences. Eur J Pain 11: 171-80.

236. Molarius A and Tegelberg A (2006) Recurrent headache and migraine as a public health problem--a population-based study in Sweden. Headache 46: 73-81.

237. Bardel A, Wallander MA, Wedel H and Svardsudd K (2009) Age-specific symptom prevalence in women 35-64 years old: a population-based study. BMC Public Health 9: 37.

238. Jonsson P, Hedenrud T and Linde M (2011) Epidemiology of medication overuse headache in the general Swedish population. Cephalalgia 31: 1015-22.

239. Wiklund M, Malmgren-Olsson EB, Ohman A, Bergstrom E and Fjellman-Wiklund A (2012) Subjective health complaints in older adolescents are related to perceived stress, anxiety and gender - a cross-sectional school study in Northern Sweden. BMC Public Health 12: 993.

240. Bille BS (1962) Migraine in school children. A study of the incidence and short-term prognosis, and a clinical, psychological and electroencephalographic comparison between children with migraine and matched controls. Acta Paediatr Suppl 136: 1-151.

241. Merikangas KR, Cui L, Richardson AK, Isler H, Khoromi S, Nakamura E, Lamers F, Rossler W, Ajdacic-Gross V, Gamma A and Angst J (2011) Magnitude, impact, and stability of primary headache subtypes: 30 year prospective Swiss cohort study. BMJ 343: d5076.

242. Sokolovic E, Riederer F, Szucs T, Agosti R and Sandor PS (2013) Self-reported headache among the employees of a Swiss university hospital: prevalence, disability, current treatment, and economic impact. Journal of Headache and Pain 14: 29.

243. Merikangas KR, Whitaker AE, Isler H and Angst J (1994) The Zurich Study: XXIII. Epidemiology of headache syndromes in the Zurich cohort study of young adults. Eur. Arch. Psychiatry Clin. Neurosci. 244: 145-152.

244. Semiz M, Senturk IA, Balaban H, Yagiz AK and Kavakci O (2013) Prevalence of migraine and co-morbid psychiatric disorders among students of Cumhuriyet University. Journal of Headache and Pain 14: 34.

245. Poyrazoglu HG, Kumandas S, Canpolat M, Gumus H, Elmali F, Kara A and Per H (2015) The prevalence of migraine and tension-type headache among schoolchildren in Kayseri, Turkey: an evaluation of sensitivity and specificity using multivariate analysis. J Child Neurol 30: 889-95.

246. Kececi H and Dener S (2002) Epidemiological and clinical characteristics of migraine in Sivas, Turkey. Headache 42: 275-280.

247. Koseoglu E, Nacar M, Talaslioglu A and Cetinkaya F (2003) Epidemiological and clinical characteristics of migraine and tension type headache in 1146 females in Kayseri, Turkey. Cephalalgia 23: 381-388.

248. Zencir M, Ergin H, Sahiner T, Kilic I, Alkis E, Ozdel L, Gurses D and Ergin A (2004) Epidemiology and symptomatology of migraine among school children: Denizli urban area in Turkey. Headache 44: 780-785.

249. Boru UT, Kocer A, Luleci A, Sur H, Tutkan H and Atli H (2005) Prevalence and characteristics of migraine in women of reproductive age in Istanbul, Turkey: a population based survey. Tohoku J. Exp. Med. 206: 51-59.

250. Celik Y, Ekuklu G, Tokuc B and Utku U (2005) Migraine prevalence and some related factors in Turkey. Headache 45: 32-36.

251. Karli N, Akis N, Zarifoglu M, Akgoz S, Irgil E, Ayvacioglu U, Calisir N, Haran N and Akdogan O (2006) Headache prevalence in adolescents aged 12 to 17: a student-based epidemiological study in Bursa. Headache 46: 649-55.

252. Unalp A, Dirik E and Kurul S (2007) Prevalence and clinical findings of migraine and tension-type headache in adolescents. Pediatr Int 49: 943-9.

253. Akyol A, Kiylioglu N, Aydin I, Erturk A, Kaya E, Telli E and Akyildiz U (2007) Epidemiology and clinical characteristics of migraine among school children in the Menderes region. Cephalalgia 27: 781-7.

254. Isik U, Topuzoglu A, Ay P, Ersu RH, Arman AR, Onsuz MF, Karavus M and Dagli E (2009) The prevalence of headache and its association with socioeconomic status among schoolchildren in istanbul, Turkey. Headache 49: 697-703.

255. Alp R, Alp SI, Palanci Y, Sur H, Boru UT, Ozge A and Yapici Z (2010) Use of the International Classification of Headache Disorders, Second Edition, criteria in the diagnosis of primary headache in schoolchildren: epidemiology study from eastern Turkey. Cephalalgia 30: 868-77.

256. Ertas M, Baykan B, Orhan EK, Zarifoglu M, Karli N, Saip S, Onal AE and Siva A (2012) One-year prevalence and the impact of migraine and tension-type headache in Turkey: a nationwide home-based study in adults. Journal of Headache and Pain 13: 147-57.

257. Wober C, Wober-Bingol C, Uluduz D, Aslan TS, Uygunoglu U, Tufekci A, Alp SI, Duman T, Surgun F, Emir GK, Demir CF, Balgetir F, Ozdemir YB, Auer T, Siva A and Steiner TJ (2018) Undifferentiated headache: broadening the approach to headache in children and adolescents, with supporting evidence from a nationwide school-based cross-sectional survey in Turkey. Journal of Headache and Pain 19: 18.

258. Bugdayci R, Ozge A, Sasmaz T, Kurt AO, Kaleagasi H, Karakelle A, Tezcan H and Siva A (2005) Prevalence and factors affecting headache in Turkish schoolchildren. Pediatr. Int. 47: 316-322.

259. Wober-Bingol C, Wober C, Uluduz D, Uygunoglu U, Aslan TS, Kernmayer M, Zesch HE, Gerges NT, Wagner G, Siva A and Steiner TJ (2014) The global burden of headache in children and adolescents - developing a questionnaire and methodology for a global study. Journal of Headache and Pain 15: 86.

260. Childs AJ and Sweetnam MT (1961) A study of 104 cases of migraine. Br J Ind Med 18: 234-6.

261. Waters WE (1973) The epidemiological enigma of migraine. Int. J. Epidemiol. 2: 189-194.

262. Green JE (1977) A survey of migraine in England 1975-1976. Headache 17: 67-68.

263. Mortimer MJ, Kay J and Jaron A (1992) Epidemiology of headache and childhood migraine in an urban general practice using Ad Hoc, Vahlquist and IHS criteria. Dev Med Child Neurol 34: 1095-1101.

264. Mounstephen AH and Harrison RK (1995) A study of migraine and its effects in a working population. Occup Med (Lond) 45: 311-7.

265. Steiner TJ, Scher AI, Stewart WF, Kolodner K, Liberman J and Lipton RB (2003) The prevalence and disability burden of adult migraine in England and their relationships to age, gender and ethnicity. Cephalalgia 23: 519-527.

266. Boardman HF, Thomas E, Croft PR and Millson DS (2003) Epidemiology of headache in an English district. Cephalalgia 23: 129-137.

267. Boardman HF, Thomas E, Millson DS and Croft PR (2005) One-year follow-up of headache in an adult general population. Headache 45: 337-345.

268. Crisp AH, Kalucy RS, McGuinness B, Ralph PC and Harris G (1977) Some clinical, social and psychological characteristics of migraine subjects in the general population. Postgrad. Med. J. 53: 691-697.

269. Deubner DC (1977) An epidemiologic study of migraine and headache in 10-20 year olds. Headache 17: 173-180.

270. Abu-Arefeh I and Russell G (1994) Prevalence of headache and migraine in schoolchildren. BMJ 309: 765-769.

271. O'Brien B, Goeree R and Streiner D (1994) Prevalence of migraine headache in Canada: a population-based survey. Int. J. Epidemiol. 23: 1020-1026.

272. Gilmour H and Wilkins K (2001) Migraine. Health Rep 12: 23-40.

273. Jette N, Patten S, Williams J, Becker W and Wiebe S (2008) Comorbidity of migraine and psychiatric disorders--a national population-based study. Headache 48: 501-16.

274. Modgill G, Jette N, Wang JL, Becker WJ and Patten SB (2012) A population-based longitudinal community study of major depression and migraine. Headache 52: 422-32.

275. Ramage-Morin PL and Gilmour H (2014) Prevalence of migraine in the Canadian household population. Health Rep 25: 10-6.

276. Hammond NG and Stinchcombe A (2019) Health Behaviors and Social Determinants of Migraine in a Canadian Population-Based Sample of Adults Aged 45-85 Years: Findings From the CLSA. Headache 59: 1547-1564.

277. Cooke LJ and Becker WJ (2010) Migraine prevalence, treatment and impact: the canadian women and migraine study. Can J Neurol Sci 37: 580-7.

278. Pryse-Phillips W, Findlay H, Tugwell P, Edmeads J, Murray TJ and Nelson RF (1992) A Canadian population survey on the clinical, epidemiologic and societal impact of migraine and tension-type headache. Can. J. Neurol. Sci. 19: 333-339.

279. Lateef T, He JP, Nelson K, Calkins ME, Gur R, Gur R and Merikangas KR (2019) Physical-Mental Comorbidity of Pediatric Migraine in the Philadelphia Neurodevelopmental Cohort. J Pediatr 205: 210-217.

280. Lateef T, Witonsky K, He J and Ries Merikangas K (2019) Headaches and sleep problems in US adolescents: Findings from the National Comorbidity Survey - Adolescent Supplement (NCS-A). Cephalalgia 39: 1226-1235.

281. Stang PE, Yanagihara T, Swanson JW, Beard CM and Melton LJ (1991) A population-based study of migraine headaches in Olmsted County, Minnesota. Case ascertainment and classification. Neuroepidemiology 10: 297-307.

282. Scher AI, Stewart WF, Liberman J and Lipton RB (1998) Prevalence of frequent headache in a population sample. Headache 38: 497-506.

283. Molgaard CA, Rothrock J, Stang PE and Golbeck AL (2002) Prevalence of migraine among Mexican Americans in San Diego, California: survey 1. Headache 42: 878-82.

284. Evans RW, Lipton RB and Silberstein SD (2003) The prevalence of migraine in neurologists. Neurology 61: 1271-1272.

285. Lipton RB, Manack A, Ricci JA, Chee E, Turkel CC and Winner P (2011) Prevalence and burden of chronic migraine in adolescents: results of the chronic daily headache in adolescents study (C-dAS). Headache 51: 693-706.

286. Rhee H (2000) Prevalence and predictors of headaches in US adolescents. Headache 40: 528-538.

287. Lipton RB, Stewart WF, Diamond S, Diamond ML and Reed M (2001) Prevalence and burden of migraine in the United States: data from the American Migraine Study II. Headache 41: 646-657.

288. Lipton RB, Scher AI, Kolodner K, Liberman J, Steiner TJ and Stewart WF (2002) Migraine in the United States: epidemiology and patterns of health care use. Neurology 58: 885-894.

289. Carson AL, Rose KM, Sanford CP, Ephross SA, Stang PE, Hunt KJ, Brown CA and Szklo M (2004) Lifetime prevalence of migraine and other headaches lasting 4 or more hours: the Atherosclerosis Risk in Communities (ARIC) study. Headache 44: 20-28.

290. Patel NV, Bigal ME, Kolodner KB, Leotta C, Lafata JE and Lipton RB (2004) Prevalence and impact of migraine and probable migraine in a health plan. Neurology 63: 1432-1438.

291. Bigal ME, Liberman JN and Lipton RB (2006) Age-dependent prevalence and clinical features of migraine. Neurology 67: 246-51.

292. Bigal ME, Lipton RB, Winner P, Reed ML, Diamond S and Stewart WF (2007) Migraine in adolescents: association with socioeconomic status and family history. Neurology 69: 16-25.

293. Lipton RB, Bigal ME, Diamond M, Freitag F, Reed ML and Stewart WF (2007) Migraine prevalence, disease burden, and the need for preventive therapy. Neurology 68: 343-9.

294. Nyame YA, Ambrosy AP, Saps M, Adams PN, Dhroove GN and Suresh S (2010) Recurrent headaches in children: an epidemiological survey of two middle schools in inner city Chicago. Pain Pract 10: 214-21.

295. Lateef T, Swanson S, Cui L, Nelson K, Nakamura E and Merikangas K (2011) Headaches and sleep problems among adults in the United States: findings from the National Comorbidity Survey-Replication study. Cephalalgia 31: 648-53.

296. Reza A, Sievert LL, Rahberg N, Morrison LA and Brown DE (2012) Prevalence and determinants of headaches in Hawaii: the Hilo Women's Health Study. Ann Hum Biol 39: 305-14.

297. Adams AM, Serrano D, Buse DC, Reed ML, Marske V, Fanning KM and Lipton RB (2015) The impact of chronic migraine: The Chronic Migraine Epidemiology and Outcomes (CaMEO) Study methods and baseline results. Cephalalgia 35: 563-78.

298. Burch R, Rizzoli P and Loder E (2018) The Prevalence and Impact of Migraine and Severe Headache in the United States: Figures and Trends From Government Health Studies. Headache 58: 496-505.

299. Ziegler DK, Hassanein RS and Couch JR (1977) Characteristics of life headache histories in a nonclinic population. Neurology 27: 265-269.

300. Duckro PN, Tait RC and Margolis RB (1989) Prevalence of very severe headache in a large US metropolitan area. Cephalalgia 15: 199-205.

301. Cook NR, Evans DA, Funkenstein HH, Scherr PA, Ostfeld AM, Taylor JO and Hennekens CH (1989) Correlates of headache in a population-based cohort of elderly. Arch. Neurol. 46: 1338-1344.

302. Stewart WF, Lipton RB, Celentano DD and Reed ML (1992) Prevalence of migraine headache in the United States. Relation to age, income, race, and other sociodemographic factors. JAMA 267: 64-69.

303. Stang PE and Osterhaus JT (1993) Impact of migraine in the United States: data from the National Health Interview Survey. Headache 33: 29-35.

304. Kryst S and Scherl E (1994) A population-based survey of the social and personal impact of headache. Headache 34: 344-350.

305. Stewart WF, Lipton RB and Liberman J (1996) Variation in migraine prevalence by race. Neurology 47: 52-59.

306. Lee LH and Olness KN (1997) Clinical and demographic characteristics of migraine in urban children. Headache 37: 269-276.

307. Schwartz BS, Stewart WF, Simon D and Lipton RB (1998) Epidemiology of tension-type headache. JAMA 279: 381-383.

308. Schwartz BS, Stewart WF and Lipton RB (1997) Lost workdays and decreased work effectiveness associated with headache in the workplace. J. Occup. Environ. Med. 39: 320-327.

309. Morillo LE, Alarcon F, Aranaga N, Aulet S, Chapman E, Conterno L, Estevez E, Garcia-Pedroza F, Garrido J, Macias-Islas M, Monzillo P, Nunez L, Plascencia N, Rodriguez C and Takeuchi Y (2005) Prevalence of migraine in Latin America. Headache 45: 106-117.

310. Vincent M, Rodrigues Ade J, De Oliveira GV, De Souza KF, Doi LM, Rocha MB, Saporta MA, Orleans RB, Kotecki R, Estrela VV, De Medeiros VA and Borges WI (1998) [Prevalence and indirect costs of headache in a Brazilian Company]. Arq Neuropsiquiatr 56: 734-43.

311. Sanvito WL, Monzillo PH, Peres MF, Martinelli MO, Fera MP, Gouveia DA, Murachovsky J, Salomao WR and Leme RJ (1996) The epidemiology of migraine in medical students. Headache 36: 316-9.

312. da Costa MZ, Soares CB, Heinisch LM and Heinisch RH (2000) Frequency of headache in the medical students of Santa Catarina's Federal University. Headache 40: 740-4.

313. Bigal ME, Fernandes LC, Moraes FA, Bordini CA and Speciali JG (2000) [Migraine prevalence and impact in employees of the clinical hospital of the medical school of Ribeirao Preto-USP]. Arq Neuropsiquiatr 58: 431-6.

314. Domingues RB, Kuster GW, Dutra LA and Santos JG (2004) Headache epidemiology in Vitoria, Espirito Santo. Arq. Neuropsiquiatr. 62: 588-591.

315. Pahim LS, Menezes AM and Lima R (2006) [Prevalence and factors associated to migraine in adult population, Southern Brazil]. Rev Saude Publica 40: 692-8.

316. Queiroz LP, Barea LM and Blank N (2006) An epidemiological study of headache in Florianopolis, Brazil. Cephalalgia 26: 122-7.

317. Queiroz LP, Peres MF, Piovesan EJ, Kowacs F, Ciciarelli MC, Souza JA and Zukerman E (2008) A Nationwide Population-Based Study of Tension-Type Headache in Brazil. Headache.

318. Queiroz LP, Peres MF, Kowacs F, Piovesan EJ, Ciciarelli MC, Souza JA and Zukerman E (2008) Chronic daily headache in Brazil: a nationwide population-based study. Cephalalgia 28: 1264-9.

319. Queiroz LP, Peres MF, Piovesan EJ, Kowacs F, Ciciarelli MC, Souza JA and Zukerman E (2009) A nationwide population-based study of migraine in Brazil. Cephalalgia 29: 642-9.

320. Arruda MA, Guidetti V, Galli F, Albuquerque RC and Bigal ME (2010) Primary headaches in childhood--a population-based study. Cephalalgia 30: 1056-64.

321. Souza-e-Silva HR and Rocha-Filho PA (2011) Headaches and academic performance in university students: a cross-sectional study. Headache 51: 1493-502.

322. Stuginski-Barbosa J and Speciali JG (2011) Frequency of headache among the employees of a rubber

company in the state of São Paulo, Brazil. Sao Paulo Med J 129: 66-72.

323. Rocha-Filho PA and Santos PV (2014) Headaches, quality of life, and academic performance in schoolchildren and adolescents. Headache 54: 1194-202.

324. Xavier MK, Pitangui AC, Silva GR, Oliveira VM, Beltrao NB and Araujo RC (2015) Prevalence of headache in adolescents and association with use of computer and videogames. Cien Saude Colet 20: 3477-86.

325. Goulart AC, Santos IS, Lotufo PA and Bensenor IM (2015) Gender aspects of the relationship between migraine and cardiovascular risk factors: A cross-sectional evaluation in the Brazilian Longitudinal Study of Adult Health (ELSA-Brasil). Cephalalgia 35: 1103-14.

326. Peres MFP, Queiroz LP, Rocha PS, Sarmento EM, Katsarava Z and Steiner TJ (2019) Migraine: a major debilitating chronic non-communicable disease in Brazil, evidence from two national surveys. Journal of Headache and Pain 20: .

327. Okamura MN, Goldbaum M, Madeira W and Cesar CLG (2020) Prevalence of headache and associated factors among adolescents: results of a population-based study. Rev Bras Epidemiol 23: e200067.

328. Arruda MA, Arruda R, Guidetti V and Bigal ME (2020) ADHD Is Comorbid to Migraine in Childhood: A Population-Based Study. J Atten Disord 24: 990-1001.

329. Wiehe M, Fuchs SC, Moreira LB, Moraes RS and Fuchs FD (2002) Migraine is more frequent in individuals with optimal and normal blood pressure: a population-based study. J. Hypertens. 20: 1303-1306.

330. Bensenor IM, Tofoli LF and Andrade L (2003) Headache complaints associated with psychiatric comorbidity in a population-based sample. Braz. J. Med. Biol. Res. 36: 1425-1432.

331. Bensenor IM, Lotufo PA, Goulart AC, Menezes PR and Scazufca M (2008) The prevalence of headache among elderly in a low-income area of Sao Paulo, Brazil. Cephalalgia 28: 329-33.

332. Domingues RB, Cezar PB, Schmidt Filho J, de Moraes Filho MN, Pinheiro MN, Marchiori JG, Brito F and Domingues SA (2009) Prevalence and impact of headache and migraine among Brazilian Tupiniquim natives. Arq Neuropsiquiatr 67: 413-5.

333. Junior AS, Krymchantowski A, Moreira P, Vasconcelos L, Gomez R and Teixeira A (2009) Prevalence of headache in the entire population of a small city in Brazil. Headache 49: 895-9.

334. da Silva A, Jr., Costa EC, Gomes JB, Leite FM, Gomez RS, Vasconcelos LP, Krymchantowski A, Moreira P and Teixeira AL (2010) Chronic headache and comorbidities: a two-phase, population-based, cross-sectional study. Headache 50: 1306-12.

335. Falavigna A, Teles AR, Velho MC, Vedana VM, Silva RC, Mazzocchin T, Basso M and Braga GL (2010) Prevalence and impact of headache in undergraduate students in Southern Brazil. Arq Neuropsiquiatr 68: 873-7.

336. Lucchetti G and Peres MF (2011) The prevalence of migraine and probable migraine in a Brazilian favela: results of a community survey. Headache 51: 971-9.

337. Arruda MA, Arruda R, Guidetti V and Bigal ME (2015) Psychosocial adjustment of children with migraine and tension-type headache - a nationwide study. Headache 55 Suppl 1: 39-50.

338. Barea LM, Tannhauser M and Rotta NT (1996) An epidemiologic study of headache among children and adolescents of southern Brazil. Cephalalgia 16: 545-549.

339. Antoniuk S, Kozak MF, Michelon L and Montemor Netto MR (1998) Prevalence of headache in children of a school from Curitiba, Brazil, comparing data obtained from children and parents. Arq Neuropsiquiatr 56: 726-33.

340. Lavados PM and Tenhamm E (1997) Epidemiology of migraine headache in Santiago, Chile: a prevalence study. Cephalalgia 17: 770-777.

341. Lavados PM and Tenhamm E (1998) Epidemiology of tension-type headache in Santiago, Chile: a prevalence study. Cephalalgia 18: 552-558.

342. Pradilla Ardila G, Vesga Angarita BE and León Sarmiento FE (2002) Estudio neuroepidemiológico en Aratoca, un área rural del oriente colombiano. Rev. med. Chile 130: 191-199.

343. Pradilla G, Vesga BE, Leon-Sarmiento FE, Bautista LE, Nunez LC, Vesga E, Gamboa NR and Grupo G (2002) [Neuroepidemiology in the eastern region of Colombia]. Rev Neurol 34: 1035-43.

344. Pradilla Ardila G, Vesga BA and Leon-Sarmiento FE (2003) Estudio neuroepidemiológico nacional (EPINEURO) colombiano. Rev Panam Salud Publica/Pan Am J Public Health 14: 104-111.

345. Diaz-Cabezas R, Ruano-Restrepo MI, Chacon-Cardona JA and Vera-Gonzalez A (2006) [Neuroepidemiology profile of the central zone of the department of Caldas (Colombia), years 2004-2005]. Rev Neurol 43: 646-52.

346. Rueda-Sanchez M and Diaz-Martinez LA (2008) Prevalence and associated factors for episodic and chronic daily headache in the Colombian population. Cephalalgia 28: 216-25.

347. Quesada-Vazquez AJ, Contreras-Maure LJ, Alvarez-Aliaga A and Traba-Tamayo ER (2009) [Prevalence of primary headaches in a rural population in Cuba]. Rev Neurol 49: 131-5.

348. Sachs H, Sevilla F, Barberis P, Bolis L, Schoenberg B and Cruz M (1985) Headache in the rural village of Quiroga, Ecuador. Headache 25: 190-193.

349. Cruz ME, Cruz I, Preux PM, Schantz P and Dumas M (1995) Headache and cysticercosis in Ecuador, South America. Headache 35: 93-97.

350. Garcia-Pedroza F, Chandra V, Ziegler DK and Schoenberg B (1991) Prevalence survey of headache in a rural Mexican village. Neuroepidemiology 10: 86-92.

351. Gracia FJ, Bayard V, Triana E, Castillo LC, Benzadon A, Larreategui M, Cerrud R and Politis S (1988) [Prevalence of neurologic diseases in Belisario Porras municipality, District of San Miguelito, Panama, 1986]. Rev Med Panama 13: 40-5.

352. Arregui A, Cabrera J, Leon-Velarde F, Paredes S, Viscarra D and Arbaiza D (1991) High prevalence of migraine in a high-altitude population. Neurology 41: 1668-9.

353. Jaillard AS, Mazetti P and Kala E (1997) Prevalence of migraine and headache in a high-altitude town of Peru: a population-based study. Headache 37: 95-101.

354. Miranda H, Ortiz G, Figueroa S, Pena D and Guzman J (2003) Prevalence of headache in Puerto Rico. Headache 43: 774-778.

355. Rondon J, Padron-Freytez A and Rada R (2001) [Prevalence of migraine among primary- and secondary-school students in Merida, Venezuela]. Rev Panam Salud Publica 9: 73-7.
