# Supplementary figures and images for: The global prevalence of headache: an update, with analysis of the influences of methodological factors on prevalence estimates
Source: J Headache Pain. 2022 Apr 12;23(1):34. doi: 10.1186/s10194-022-01402-2 (PMC9004186; doi:10.1186/s10194-022-01402-2)

# Appendix 2


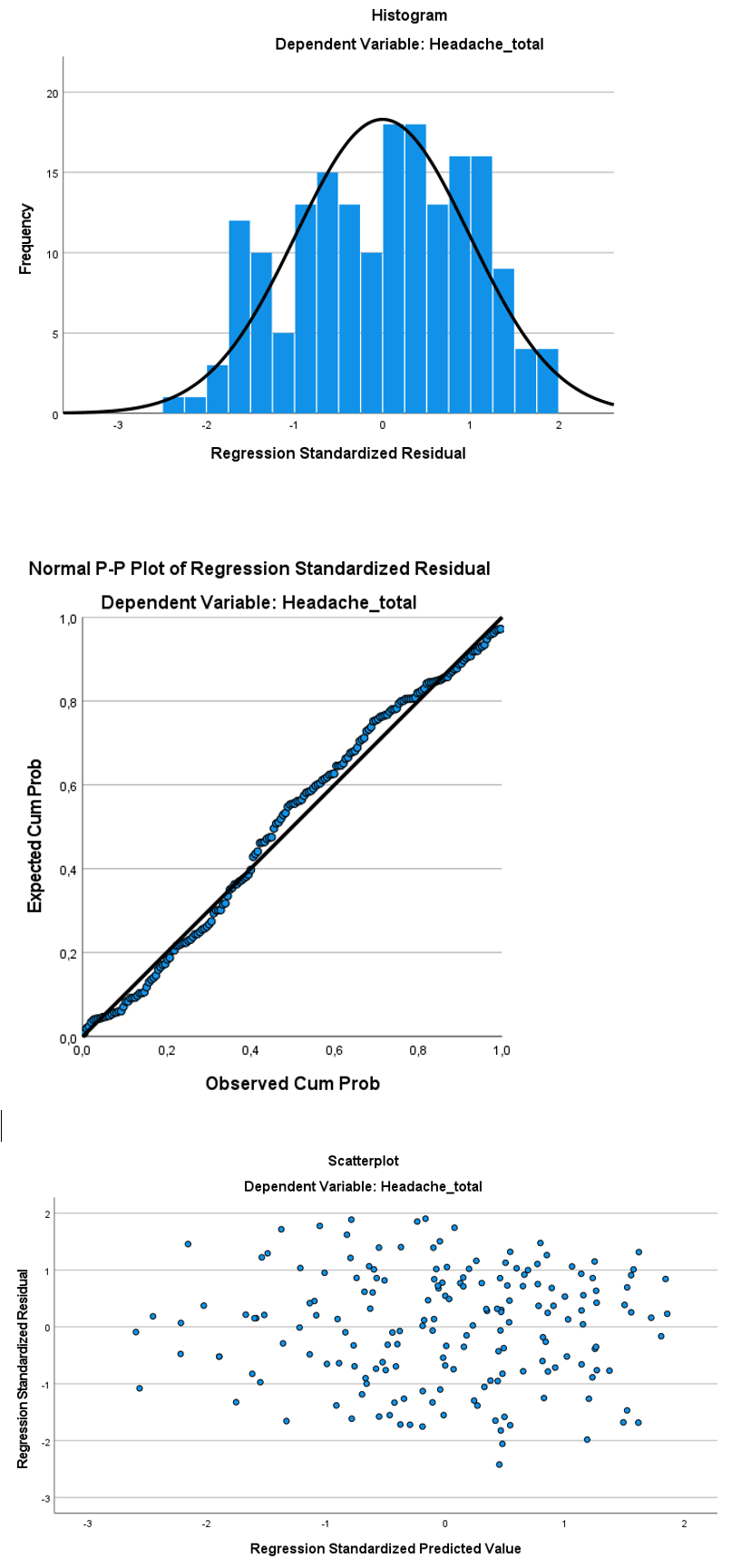


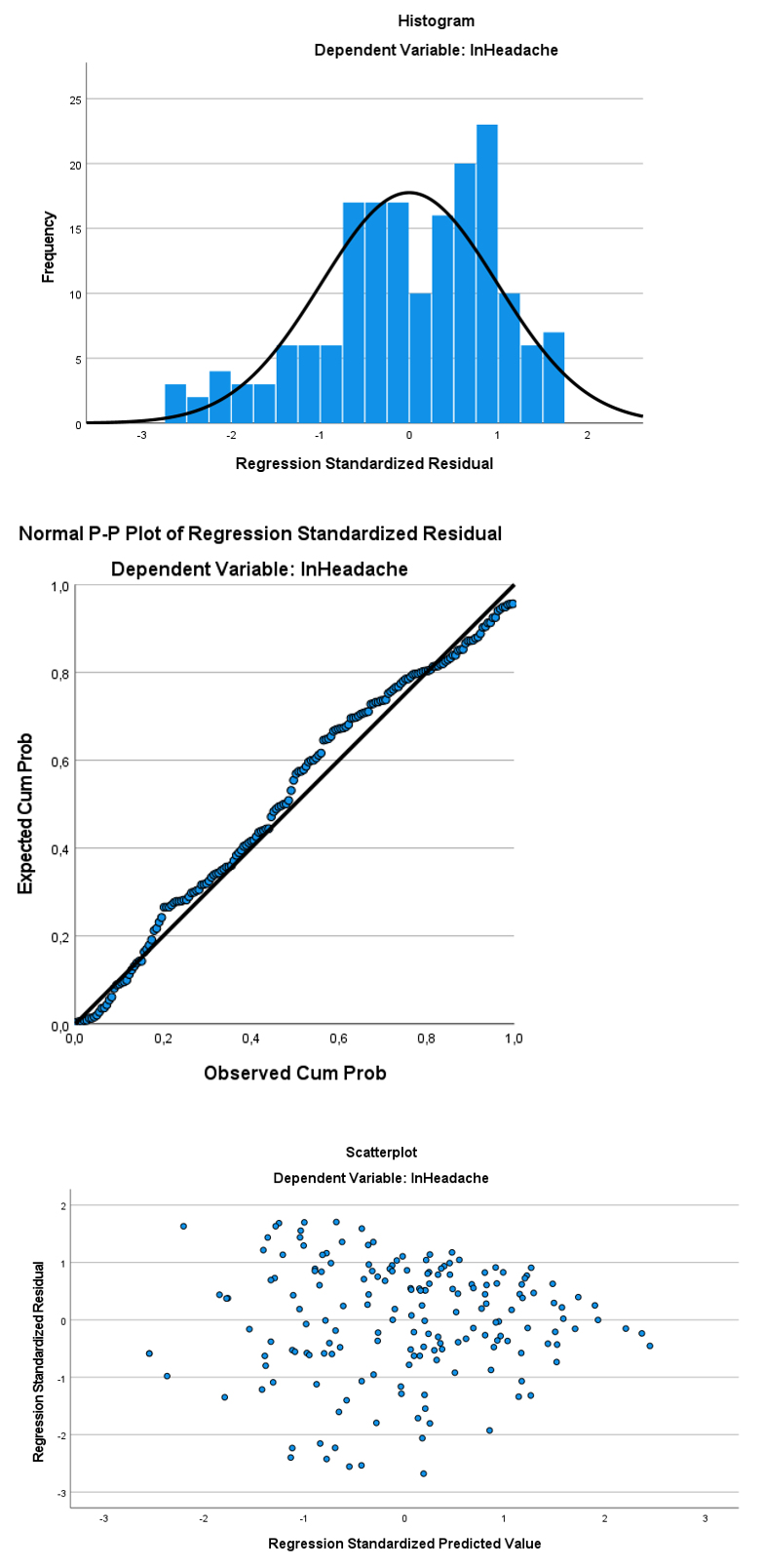


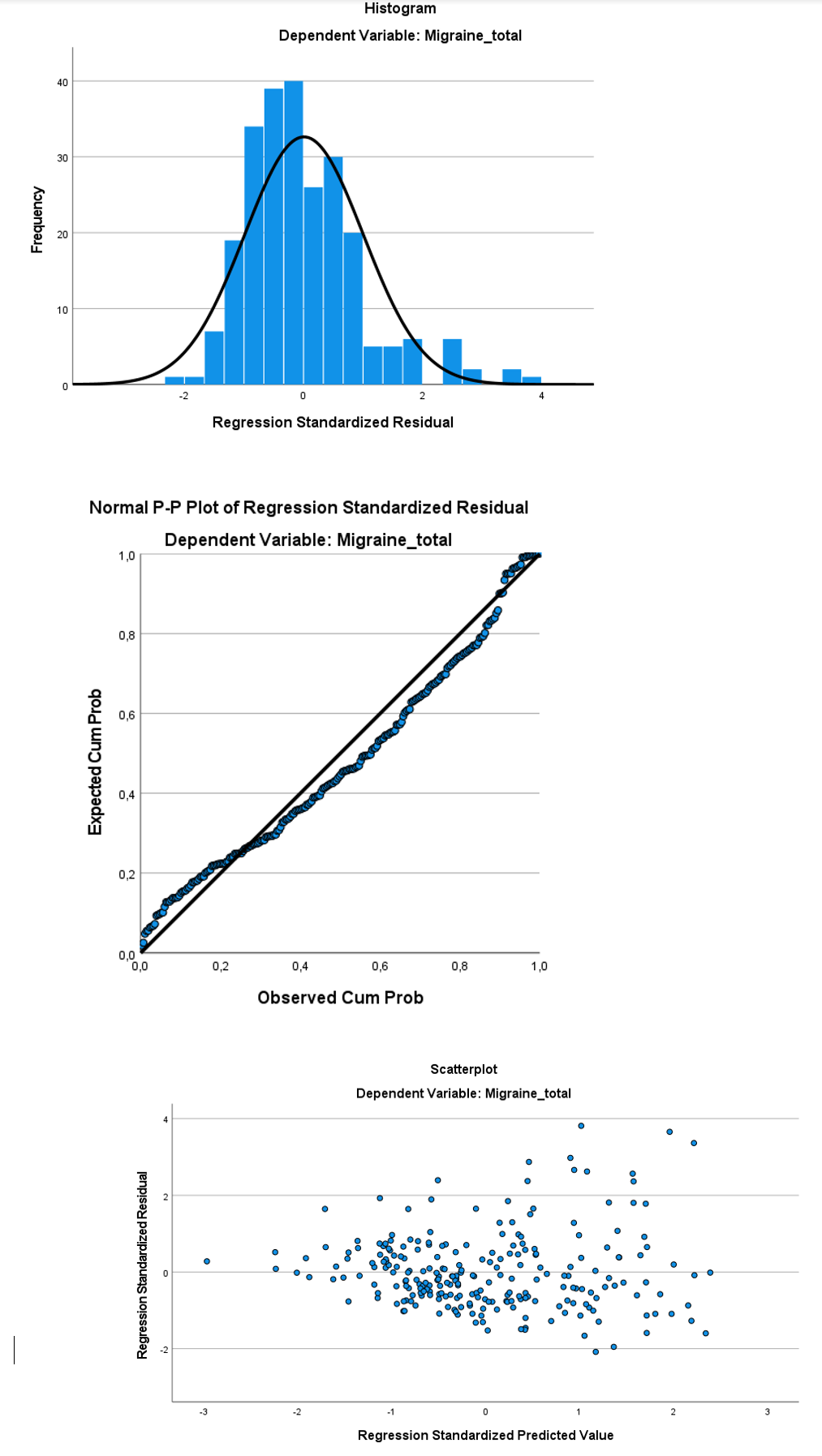


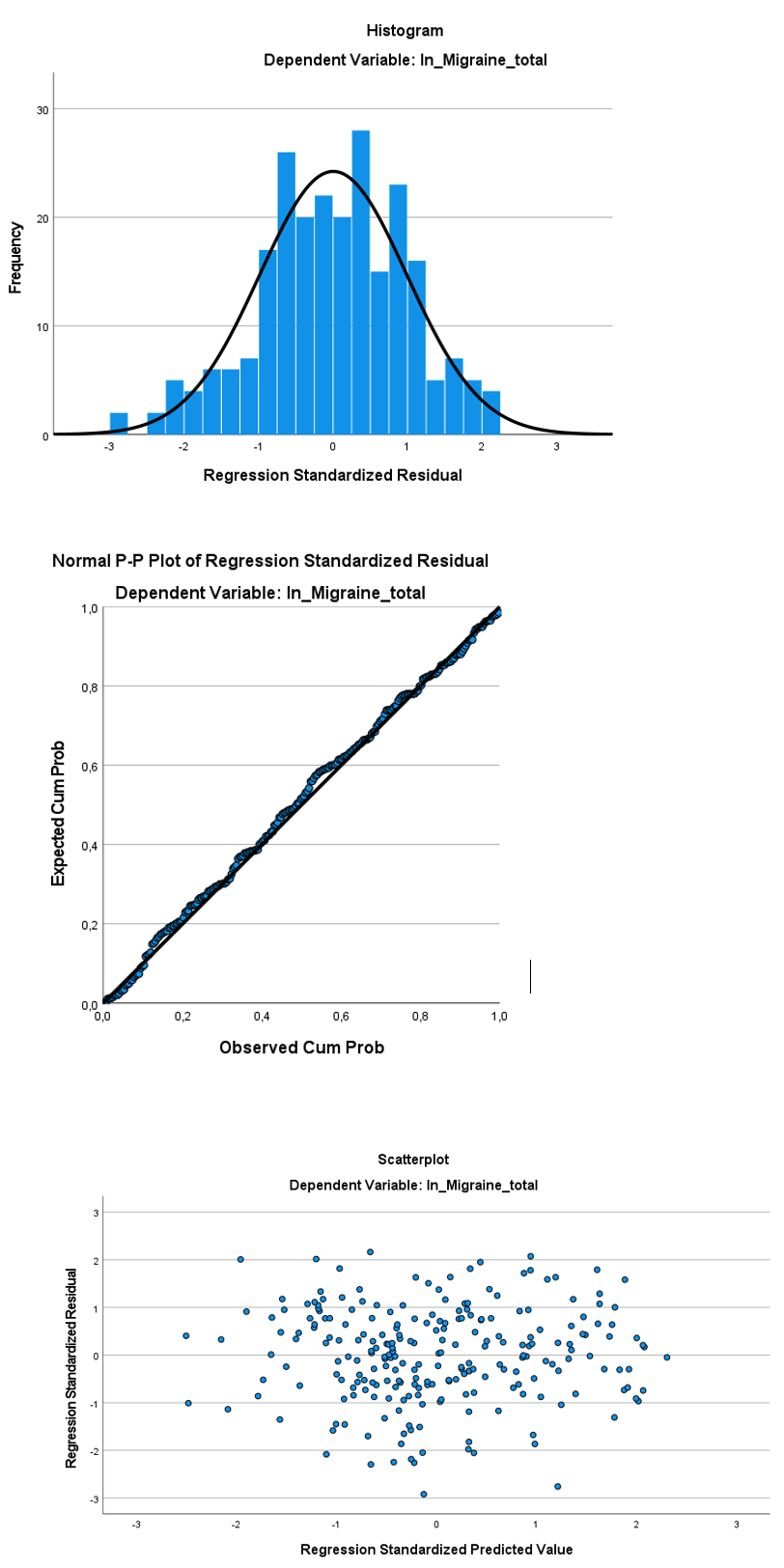


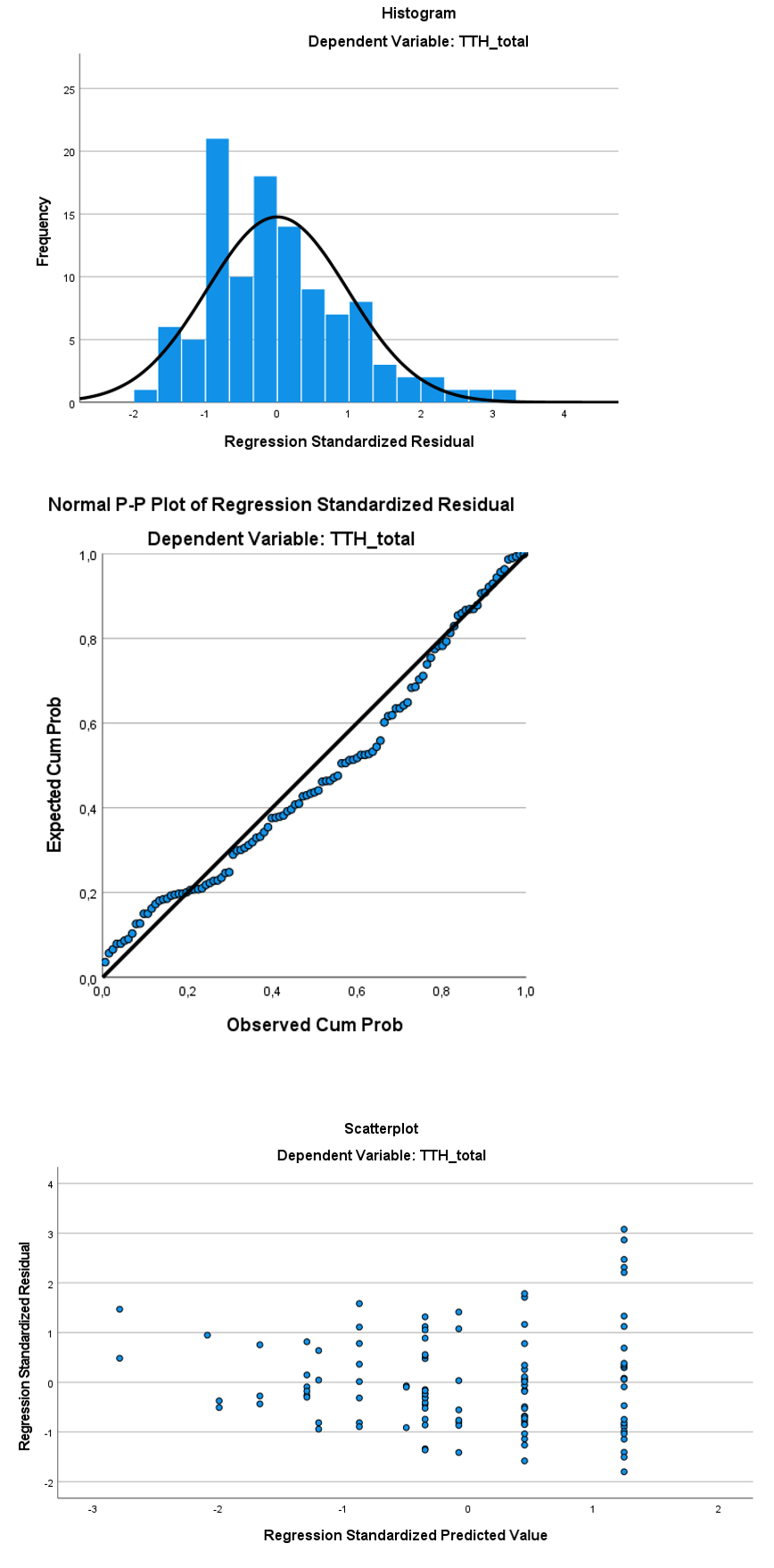


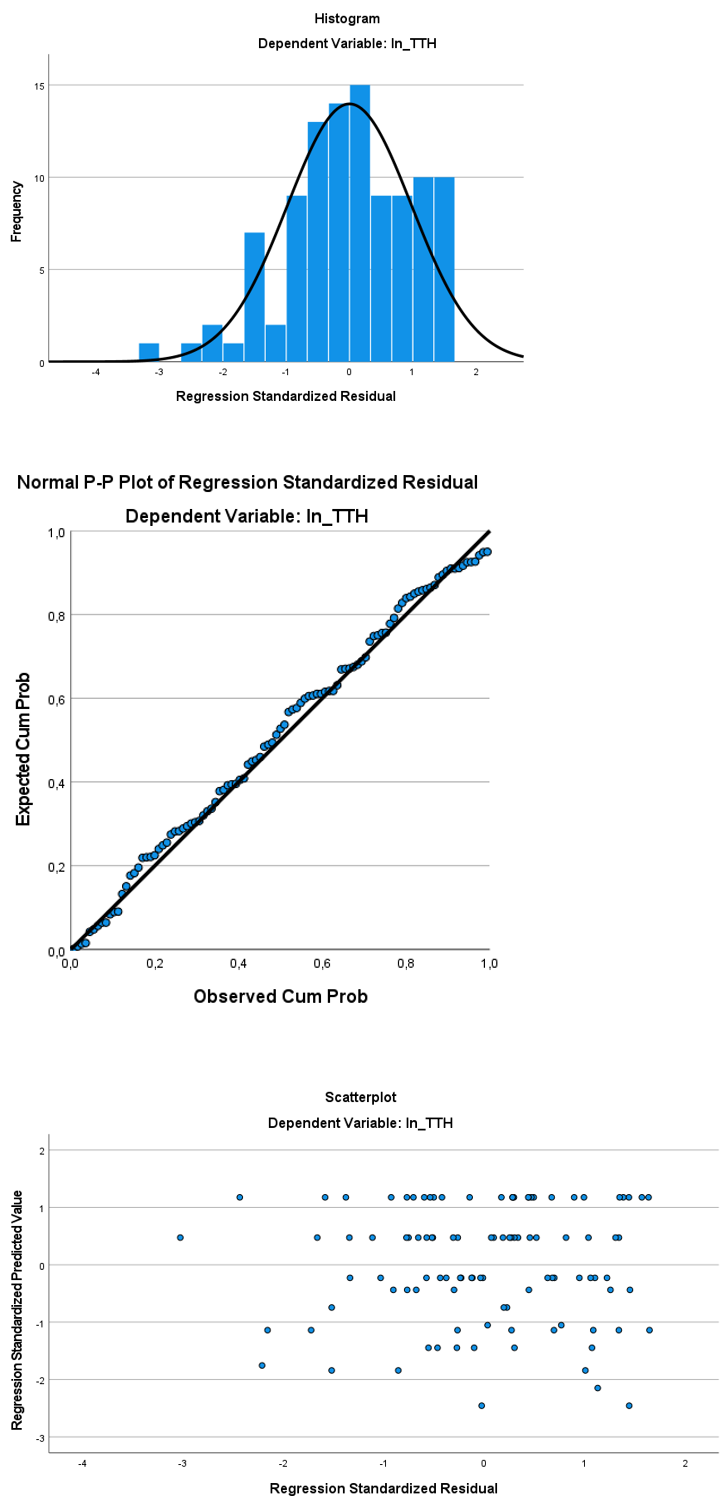


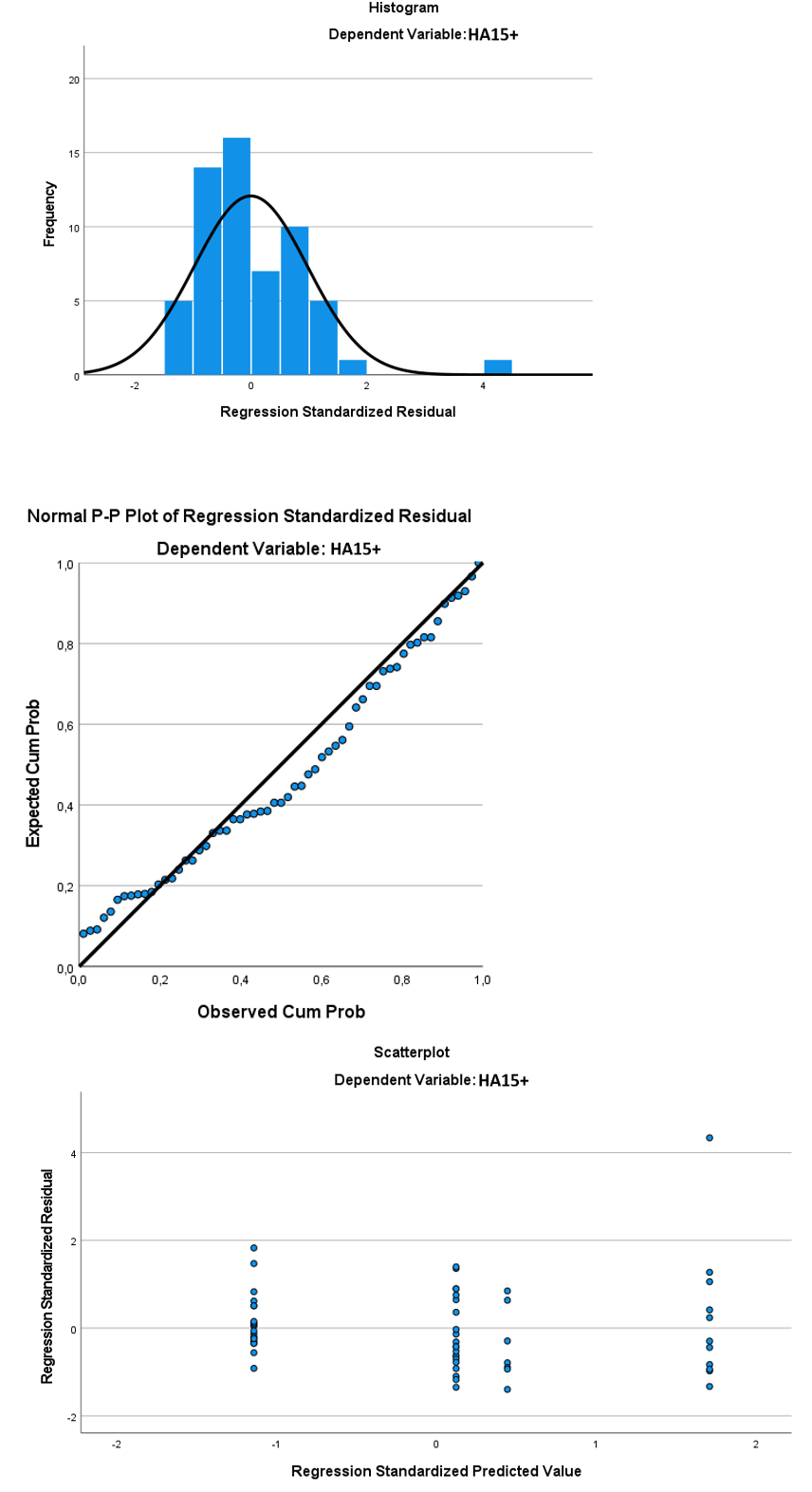


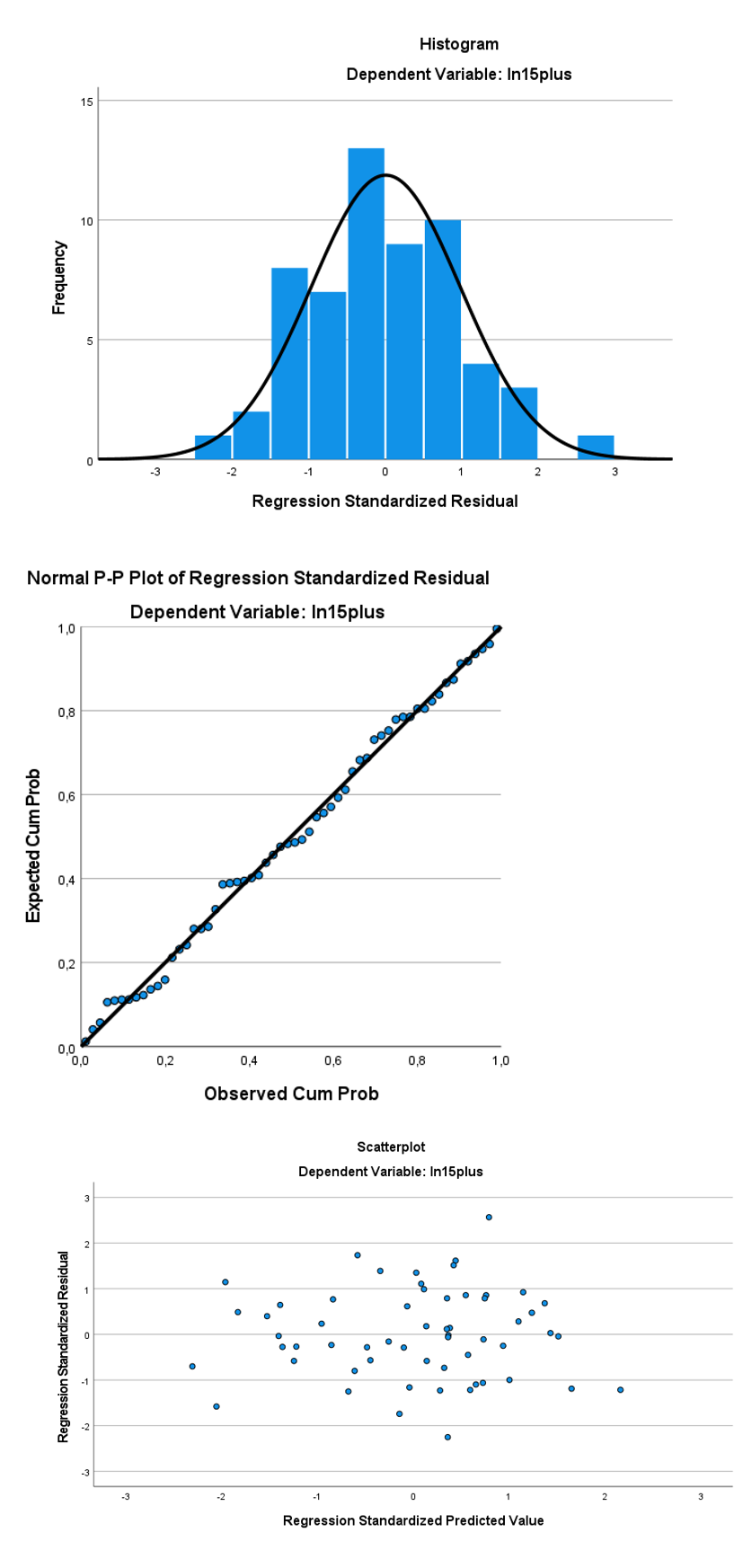

Supplement: Supplementary file 2 — Additional file 2. [file 10194_2022_1402_MOESM2_ESM.docx]
